# Impact of extreme precipitation events on facility-based births in 21 sub-Saharan African countries

**DOI:** 10.1038/s41467-026-72547-w

**Published:** 2026-05-04

**Authors:** Oumar Aly Ba, Fleur Hierink, Cameron Taylor, Peter M. Macharia, Lenka Beňová, Jérémy Laurent-Lucchetti, Nicolas Ray

**Affiliations:** 1 GeoHealth group, Institute of Global Health, Faculty of Medicine, University of Geneva, Geneva, Switzerland; 2 Institute for Environmental Sciences, University of Geneva, Geneva, Switzerland; 3 Department of Public Health, Institute of Tropical Medicine, Antwerp, Belgium; 4 The DHS Program, ICF, Rockville, MD USA; 5 Medicine and Health Sciences, University of Antwerp, Antwerp, Belgium; 6 Population & Health Impact Surveillance Group, Kenya Medical Research Institute-Wellcome Trust Research Programme, Nairobi, Kenya; 7 Institute of Economics and Econometrics, GSEM, University of Geneva, Geneva, Switzerland

**Keywords:** Health care economics, Health policy, Environmental health, Developing world, Environmental impact

## Abstract

Climate change impacts on health outcomes are increasingly recognized, yet the effects of Extreme Precipitation Events (EPEs) on geographical access to and timely use of health facilities for childbirth remain underexplored. We assess how EPEs influence facility-based births in 21 sub-Saharan African countries by combining Demographic and Health Survey data (2015–2021) with gridded daily precipitation data at 5 × 5 km resolution. Using a linear probability model with a three-day exposure window preceding each date of birth, we analyze 256,101 live births from 12,948 locations and define EPEs as daily rainfall exceeding the 85th percentile of the local historical distribution. We find that each additional day of EPE exposure within the three-day window reduces facility-based births by −10.8 per 1000 live births (95% CI −18.3 to −3.2), representing a 1.9% decrease from baseline. This reduction remains consistent across varying EPE definitions (50th–95th percentile) and becomes less pronounced with longer exposure windows, indicating that events closer to the birth date have greater impact. Sustained moderate to heavy rainfall over multiple days also lowers facility use, indicating that barriers can arise both suddenly and cumulatively. We estimate approximately 29,084 additional non-facility births (95% CI 8660 to 49,508) are attributable to EPEs in 2015. Our findings provide evidence that EPE exposure increases non-facility births that may lack the safety net of skilled attendance and emergency obstetric care.

## Abstract (web summary)

Across 21 sub-Saharan African countries, heavy rainfall around childbirth was linked to fewer deliveries in health facilities, indicating that extreme precipitation may hinder geographical access to delivery care.

## Introduction

Considerable progress has been made in improving maternal health worldwide, notably with a 38% decline in global maternal mortality between 2000 and 2017^1^. This success reflects a significant step towards safer maternal and newborn health (MNH) outcomes, largely attributed to increased skilled birth attendance and improved health service delivery^2^. Despite these gains, progress has stalled or even reversed in several countries since 2017^3^. The daily loss of 810 women globally due to pregnancy and childbirth complications underscores the ongoing challenge and the urgent need to step up efforts towards achieving Sustainable Development Goal (SDG) 3.1. This goal ambitiously aims to reduce the global maternal mortality ratio (MMR) to less than 70 per 100,000 live births by 2030^1^.

However, the growing challenge of Extreme Precipitation Events (EPEs) due to anthropogenic global warming and their geographical shift in Africa may pose a serious threat to the MNH progress made so far^4–9^. EPEs can affect the provision, utilization, and quality of MNH care in several ways; one of the most important being geographical access to care. Geographical accessibility to healthcare, encompassing the availability and condition of roads and transportation, has been recognized as an important determinant of health service utilization and provision, and remains a major barrier^10–13^. Infrastructure disruptions caused by EPEs—such as flooding, damage to roads and health facilities—can severely limit timely access to essential MNH services, in both urban and rural settings^14–21^. Additionally, even usual rainy seasons can generate these accessibility issues as seasonal and inter-annual variations in precipitation, coupled with the El Niño Southern Oscillation, play a crucial role in modulating flood frequencies in various regions of Africa^16,17,19,22–26^. This compromised accessibility of MNH services may influence the decision-making process regarding birth location and potentially increase the proportion of non-facility-based births, which lack the safety net of skilled birth attendance and adequate medical resources in case of an obstetric or newborn emergency^2,19,27,28^.

While direct links between EPEs and facility-based births are limited, some research highlights the influence of seasonal rainfall on geographical accessibility to healthcare facilities^22,23,25,29^. These small-scale studies have mainly concentrated in flood-prone areas and often dichotomize seasons into “rainy” versus “dry” a coarse classification that masks event severity. Another study documented the disruptive potential of specific events, such as the 2019 Cyclone Idai in Mozambique, on healthcare system^14^. Additional studies provided suggestive evidence of the detrimental impact of incidental floods on MNH service utilization^19,20^. This understanding is further complemented by research employing accessibility modeling techniques to simulate the reduced accessibility caused by floods and daily records of precipitation^15,17,24^. However, few studies comprehensively assess how extreme weather events broadly affect geographical access to healthcare and consequently influence maternal health outcomes^30–33^.

In this study, we aim to bridge this gap by examining the relationship between EPEs and the use of health facilities for childbirth in sub-Saharan Africa (SSA). We hypothesize that exposure to EPEs—not exclusively related to floods—negatively impacts geographical accessibility of health facilities and thus reduces the likelihood of facility-based births. By focusing on a critical window of three days (from the day of birth to 2 days before), we provide new insights into the immediate impact of EPEs on maternal health-seeking behavior, providing valuable information for policy formulation and strategic planning to safeguard and continue the gains made in maternal and newborn survival and well-being.

## Results

### Population characteristics

The resulting dataset contained data on 256,101 live births from 21 SSA countries (Supplementary Tables 1–3). Of these births, 168,581 (65.83%; 57.69% in the weighted sample) occurred in health facilities, with South Africa, Rwanda, and Malawi having the highest coverage of facility-based births (at least 92%), while Madagascar, Ethiopia, Nigeria, and Angola had the lowest (at most 41%) (Supplementary Table 4). 25,500 live births (9.95%) were exposed to at least one day of EPE, with Uganda, Burundi and Liberia being the countries with the highest share of exposed live births (Supplementary Table 5). The share and the standard deviation of facility-based births and EPE by DHS cluster are shown in the Supplementary Information, along with the distribution of the 85 percentile cutoff value used to define exposure to EPE in our DHS sample (Supplementary Figs. 1–3).

### Impact of EPEs on facility-based births

**Fig. 1:**
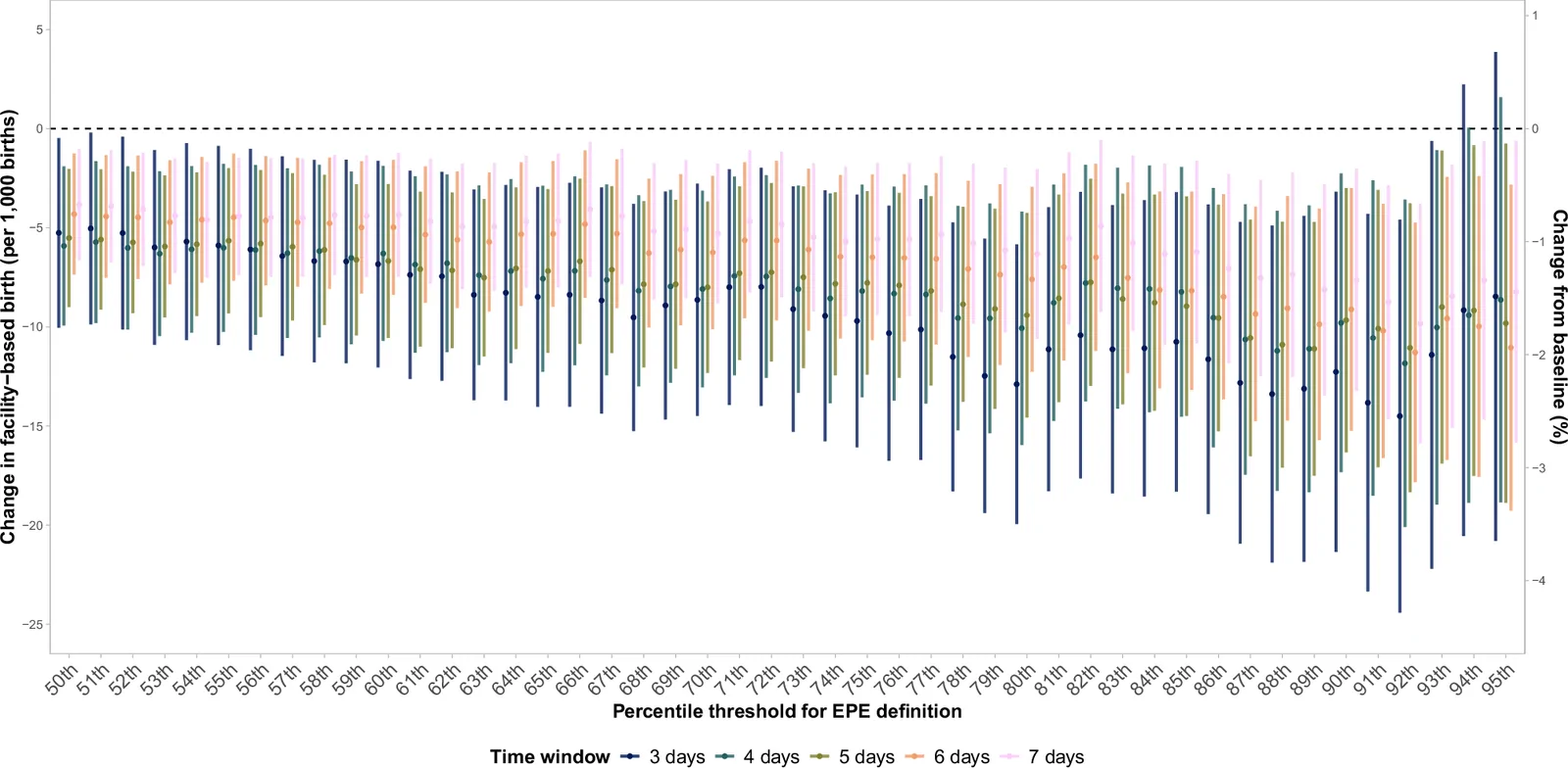
Effect of extreme precipitation events on facility-based birth across percentile thresholds and time windows (3–7 days). Point estimates represent the change in facility-based birth rate (per 1000 births) for an additional day with extreme precipitation. An extreme precipitation event (EPE) is defined as a daily rainfall realization over a percentile threshold of the local rainfall distribution. All specifications include maternal and household covariates and fixed effects for Demographic and Health Surveys (DHS) clusters and country-day-of-birth. Standard errors are clustered at the DHS cluster level. Error bars indicate 95% confidence intervals around the point estimates. For results covering extended exposure windows up to 14 days, see Supplementary Fig. 5.

We found that EPEs significantly reduced the probability of giving birth in a facility (Table 1). In our main model (column (4) of Table 1), an additional day of extreme precipitation in the three-day window leading up to and including the day of birth was associated with a decrease of −10.753 facility-based births per 1000 live births (95% CI −18.304 to −3.202). This decrease represents 1.886% of the weighted sample mean (570.027 per 1000 live births). We found that lower thresholds for EPE decreased the size of the effect (Fig. 1 and Supplementary Fig. 4). On the contrary, the effect was more pronounced when considering a more extreme definition of EPEs, with a reduction of −14.500 facility-based births per 1000 live births (95% CI −24.412 to −4.588) when considering a 92nd percentile threshold of precipitation. When considering longer time window exposures, the effect consistently diminished across all definitions of EPEs, indicating that events occurring closer to the day of birth have a greater impact than those further back in time (Fig. 1 and Supplementary Fig. 5).

**Table 1:** Effects of extreme precipitation events on facility-based birth

	Facility-based birth (per 1000 births)			
	(1)	(2)	(3)	(4)
*N* of days over 85 percentiles	−17.257***	−10.867***	−11.106***	−10.753***
	(4.079)	(3.331)	(3.156)	(3.852)
DHS cluster FE		Yes	Yes	Yes
Day of birth FE			Yes	
Country-day of birth FE				Yes
Maternal and household controls	Yes	Yes	Yes	Yes
Sample mean	576.998	576.090	576.064	570.027
Observations	256,101	255,999	255,960	252,607
*R*^2^	0.241	0.537	0.558	0.637

The estimates represent the change in the probability of facility-based birth (per 1000 births) for live births exposed to an additional day with an extreme precipitation event (EPE), compared with live births born in the same area with no or fewer extreme precipitation days. The analysis focuses on events occurring within a three-day time window from the day of birth and the two preceding days. The exposure thus ranges from 0 (no EPE) to 3 days (EPEs occurring on all three days). An extreme precipitation event is defined as a daily rainfall realization over the 85th percentile of the local rainfall distribution. Columns (1)–(4) present estimates from progressively adjusted model specifications, with column (4) corresponding to the fully adjusted main model discussed in the text. All specifications include maternal and household covariates. FE denotes fixed effects, and DHS refers to the Demographic and Health Surveys. Standard errors are clustered at the DHS cluster level. All tests are two-sided. No adjustment for multiple comparisons was made. Significance codes: ***P < 0.01, **P < 0.05, *P < 0.1.

### Sensitivity analysis

Our results are robust to multiple robustness checks shown in Supplementary Information Section 3. First, our results are robust to the inclusion of the full DHS sample that includes 4582 live births with missing precipitation matched to the nearest pixel with recorded precipitation information (Supplementary Table 6). Second, we obtained similar estimates when using a probit regression model instead of a linear probability model (Supplementary Table 7). Third, the distributed lag linear model showed that only EPE exposure in the three days immediately preceding birth led to a significant decline in facility-based births, with no evidence of spillover or anticipation effects. This reinforces the plausibility of an acute, short-term impact and supports the assumption that the timing of both birth and EPE occurrence is quasi-random (Supplementary Fig. 6). Fourth, our placebo test, which involved 10,000 random permutations of EPE timing within ±14 days of each birth date, confirmed that the observed average effect is specific to actual EPE timing rather than unobserved local seasonal patterns. Our baseline estimate (−10.753 facility-based births per 1000 live births) falls at the 0.29th percentile of the placebo distribution, with only 29 out of 10,000 placebo estimates more extreme (Supplementary Fig. 7). This result underscores the robustness of our findings regarding the causal impact of EPEs on facility-based births. Fifth, we restricted our sample to the most recent live birth reported by each woman in the 5 years preceding the survey. This reduced our sample size by 32.54%, but we still obtained similar and significant estimates, albeit with greater uncertainty (Supplementary Table 8). Sixth, our main results were consistent across alternative precipitation indices based on ETCCDI conventions: the same fading out pattern of estimates emerged when we varied absolute precipitation thresholds and time window exposure (Supplementary Fig. 8). We also found evidence of non-linearity when considering percentile-based and absolute-threshold precipitation exceedance totals, with a decreasing marginal effect (Supplementary Tables 9 and 10). Finally, our results remained qualitatively similar across various fixed effect specifications, although some estimates were less precise and not statistically distinguishable from zero (Supplementary Table 11).

### Extended analysis with the sustained rainfall exposure model

We conducted an extended analysis using a sustained (accumulation-window) rainfall exposure model, in which exposure is defined as a binary indicator for rolling rainfall totals over 3–14 day windows that exceed location-specific percentile thresholds (50th–95th) of the historical distribution of these rolling sums, constructed using only wet windows (total precipitation ≥ 1 mm). This definition captures multi-day events and complements the daily EPE counts in our baseline acute (time-window) rainfall exposure model (Supplementary Information Section 4.1 and Supplementary Fig. 9). Results indicate that persistent moderate-to-heavy rainfall significantly reduces facility-based births, highlighting two complementary risk mechanisms: acute daily shocks and sustained multi-day disruptions (Supplementary Information Section 4.1 and Supplementary Fig. 10). Across percentile thresholds, the marginal effect of daily EPEs is strongest for short time windows (3 days), whereas the effect of sustained rainfall peaks for 4–6 day windows, indicating that these shorter accumulation periods are the most critical for access disruption.

### Extended analysis by type of health facility and care at birth

In extended analyses, we disaggregated the facility-based birth outcome by type of health facility and also examined skilled attendance at birth as a separate outcome. The direction of the EPE effect remained qualitatively consistent across nearly all the analyses: EPEs reduced the likelihood of facility-based birth and skilled birth care. We observed a shift from public hospitals and non-public facilities (all levels) toward non-facility births (Supplementary Table 12), and from skilled to non-skilled care (Supplementary Table 13). However, evidence regarding a shift toward ‘no care’ remained inconclusive (Supplementary Table 13).

### Heterogeneity of EPE impacts

**Fig. 2:**
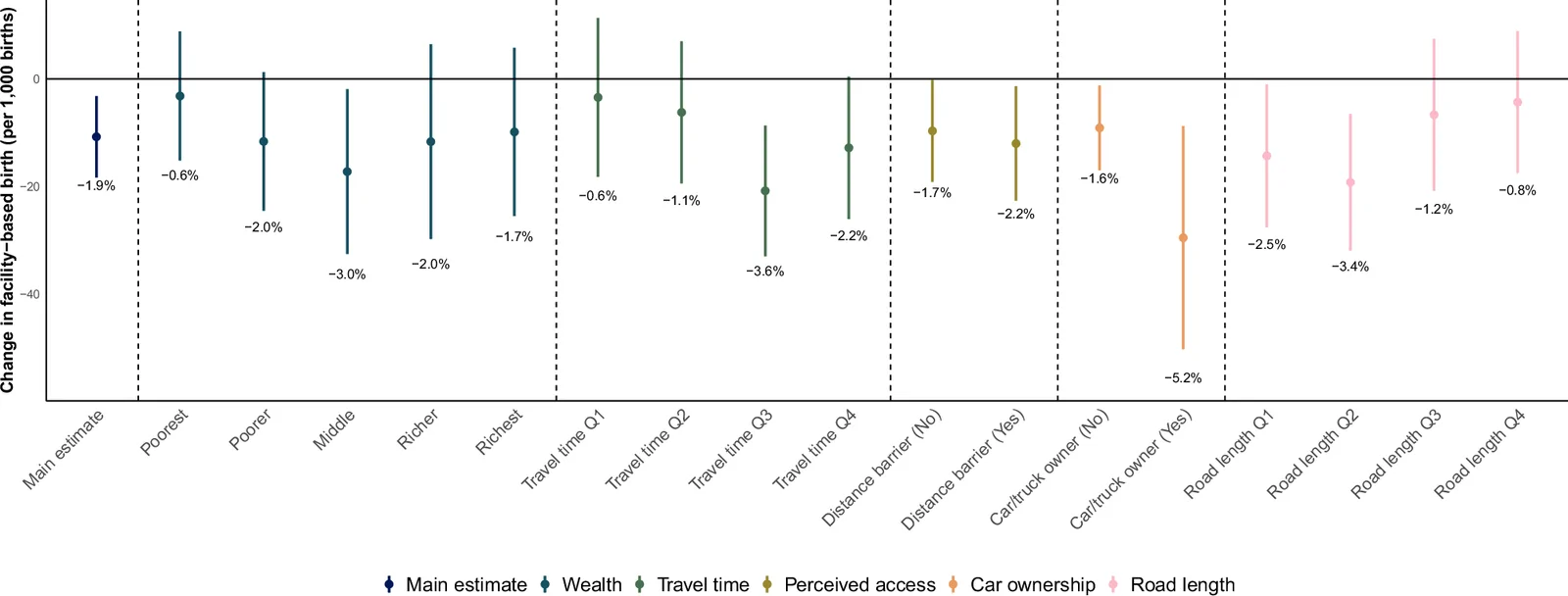
Heterogeneity in the effect of extreme precipitation events. Point estimates represent the change in the probability of facility-based birth (per 1000 births) for live births exposed to an additional day with an extreme precipitation event (EPE). The labels represent the change from the average prevalence of facility-based birth in the entire sample. The analysis focuses on events occurring within a three-day time window from the day of birth and the two preceding days. The exposure thus ranges from 0 (no EPE) to 3 days (EPEs occurring on all 3 days). An extreme precipitation event is defined as a daily rainfall realization over the 85th percentile of the local rainfall distribution. Each block of estimates (wealth, travel time, perceived access, car ownership, road length) is obtained from a separate multivariable model for the full sample, in which the EPE term is interacted with the corresponding group variable. All specifications include maternal and household covariates and fixed effects for Demographic and Health Surveys (DHS) clusters and country-day-of-birth. Standard errors are clustered at the DHS cluster level. Error bars indicate 95% confidence intervals around the point estimates. Wealth quintiles and travel time and road length quartiles are ordered from lowest to highest values (Q1 = lowest). The corresponding regression tables are presented in Supplementary Tables 14–17.

We explore heterogeneity of the effects by incorporating interaction terms (Fig. 2 and Supplementary Tables 14–17). The effect of EPEs was more pronounced among households in the third and second wealth quintiles. Specifically, women from households in the third wealth quintile experienced a significant reduction in facility births (−17.212 per 1000 live births, 95% CI −32.510 to −1.913, −3.019% decrease from the sample mean). While this effect was not significantly different from the estimate for the lowest quintile (*p* = 0.1366), it represents an erosion of about a 27% of the third wealth quintile’s baseline advantage in facility-based delivery relative to the lowest wealth quintile in the absence of EPE exposure, calculated as –17.2/63.9 (Supplementary Table 14). Rescaling across all wealth quintiles reveals a consistent gradient: EPEs erode about 35% of the facility-use advantage for households in the second wealth quintile, 27% for those in the third wealth quintile, but only 11% and 7% for the fourth and highest wealth quintiles, respectively (Supplementary Table 14). Although these differences are not statistically significant, the pattern suggests that gains in maternal health access among lower- and middle-wealth households may be more fragile and vulnerable to climate-related shocks. Women living in clusters with longer travel times to health facilities had more pronounced effects of EPEs, although the interactions do not significantly differ from each other (quartile 1 versus quartile 3: *p* = 0.0585; quartile 2 versus quartile 3: *p* = 0.0978; Supplementary Table 15). Women in areas within the third travel time quartile (e.g., the third group with the longest national travel times) had a significant decrease of −20.788 facility-based births per 1000 live births (95% CI −32.929 to −8.645). However, when considering the median travel time for interaction instead of quartiles, interaction terms were significantly different with a greater effect for women in areas above the national median travel time (*p* = 0.0465, Supplementary Table 15). Perceived distance to a health facility as a barrier, self-reported on the DHS, showed a significant reduction in facility births during EPEs for both groups, while the interaction terms were not significantly different. Car-owning households displayed a more pronounced response to EPEs, with a decrease of −29.492 per 1000 live births (95% CI −50.195 to −8.787, −5.173% decrease from the sample mean). Although the estimated difference in EPE response relative to non-car-owning households was not statistically significant (*p* = 0.0638), the point estimates suggest that EPE-related disruptions may offset the baseline advantage associated with car ownership (18.038 per 1000 live births, 95% CI 5.937 to 30.138). Road infrastructure, a public good complementary to motorized vehicles, seemed to play a role, with evidence suggesting that shorter lengths of major roads were associated with a significant decrease in facility-based births during EPEs (quartile 1: −14.287 per 1000 live births, 95% CI − 27.551 to − 1.023; quartile 2: −19.193 per 1000 live births, 95% CI −31.881 to −6.505). In the exploratory analysis by climate zone (Supplementary Table 18), point estimates were largest in tropical zones for both the acute (time-window) and sustained (accumulation-window) rainfall exposure models, though between-zone differences were generally not statistically significant, and results should be interpreted with caution given the large standard errors of the temperate and arid zones.

### Non-facility births attributed to EPEs in 2015

**Fig. 3:**
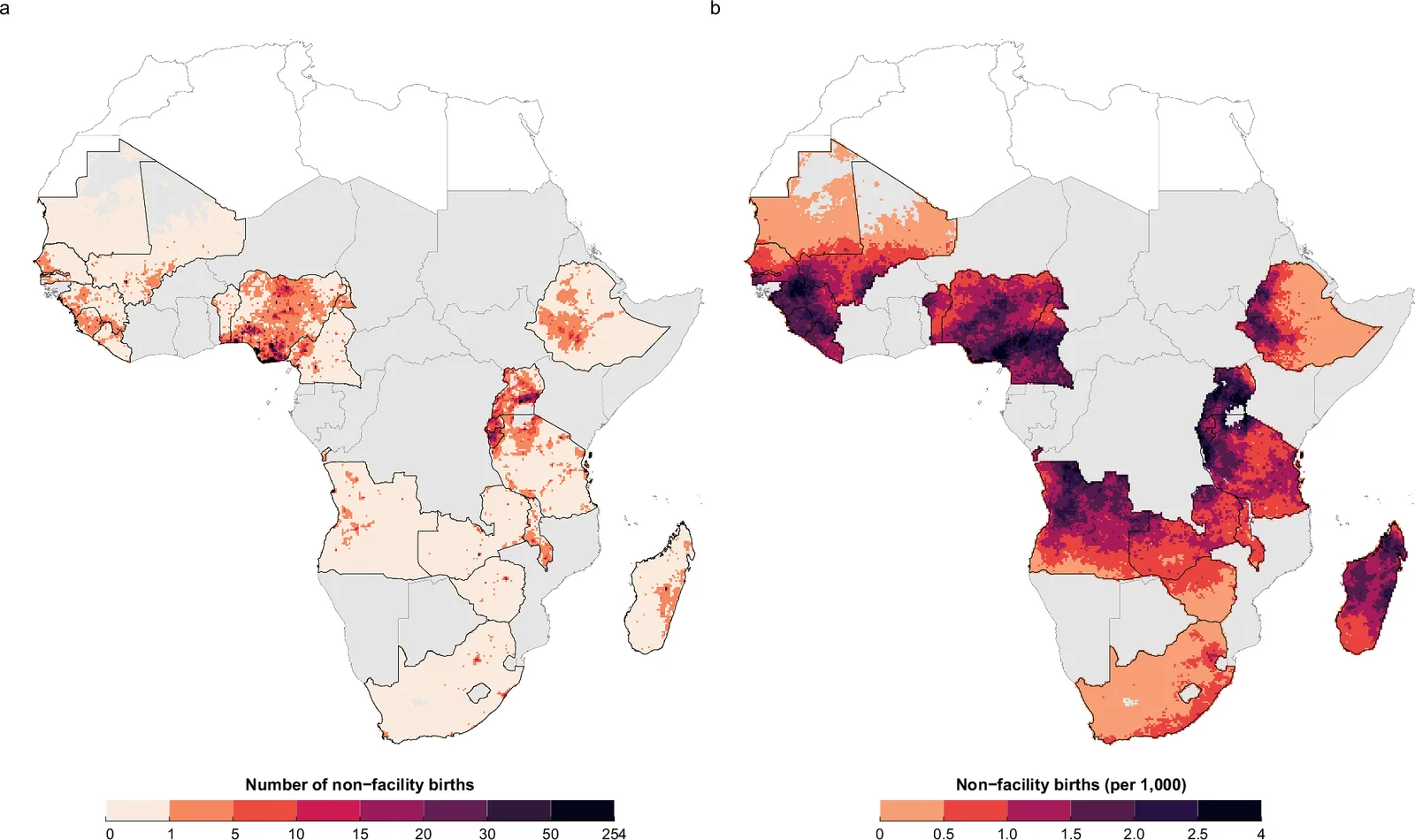
Non-facility birth and extreme precipitation events in 2015. **a** Number of non-facility births attributable to extreme precipitation events (EPEs) in 2015 per 20 × 20 km^2^ cell. **b** Number of non-facility births per 1000 live births attributable to EPEs in 2015 per 20 × 20 km^2^ cell. Sub-Saharan Africa is shown in gray and North Africa in white, with study countries outlined in black. Administrative boundaries were derived from the Database of Global Administrative Areas (GADM)^69^.

We estimated that in 2015, 29,084 non-facility births (95% CI 8,660 to 49,508) were attributable to EPEs in the 21 countries (Fig. 3a and Supplementary Fig. 11). The regions most affected were Nigeria and Uganda, which collectively accounted for 47.5% of all additional non-facility births. The number of attributable non-facility births per 1000 live births was higher around Lake Victoria, Guinea, Sierra Leone, south-east Nigeria, northern Cameroon, western Ethiopia, and northern Angola (Fig. 3b).

Finally, we explored the stability of the attribution of additional non-facility births using country-specific coefficients (Supplementary Figs. 12 and 13). Liberia, Nigeria, and Mali showed the greatest increase in the likelihood of non-facility births, with country-specific coefficients implying a change of 4.1%, 6.2%, and 3.1% relative to their national average facility-based births. In contrast, Madagascar—the only country in our study frequently exposed to cyclones—shows a positive country-specific coefficient (27.082 per 1000 live births, 95% CI − 1.635 to 55.799, *p* = 0.0645), which implies an increase in facility-based births attributable to EPEs in the attribution exercise, although the estimate is imprecise and not statistically distinguishable from zero. This pattern may reflect context-specific preparedness or behavioral responses associated with cyclone-related rainfall.

## Discussion

This study provides robust evidence of the adverse impact of EPEs on facility-based birth in SSA. We find that acute EPEs significantly reduce the likelihood of births in health facilities, a key step for providing high-quality care during labor and childbirth. Prolonged moderate-to-heavy rainfall, accumulated over multiple days, also exerts a strong impact, highlighting a second mechanism of sustained multi-day disruptions. We estimate that approximately 29,084 non-facility births (95% CI 8660 to 49,508) are attributable to EPEs in 2015. We also find that EPEs incentivize a shift from skilled to non-skilled birth care attendance.

Our findings align with and extend the five-phase mortality transition model proposed by Boerma and colleagues^2^. This model suggests that transitioning out of high-mortality phases (characterized by maternal mortality, stillbirths, and neonatal mortality) involves increased facility-based births, initially in lower-level facilities and later in hospitals, with wealth-related inequalities in facility-based births coverage diminishing in later phases. Our study complements this framework by showing that EPEs can disrupt the utilization of these potential higher-quality childbirth care services. This disruption may also particularly affect households in the third wealth quintile, which are likely leading the transition to such newly adopted health-seeking behaviors, albeit still fragile. A plausible explanation is that these households may be more vulnerable to climate-related access barriers because, although more likely to own motorized vehicles, they still rely on road infrastructure that is fragile and highly susceptible to extreme rainfall. EPE disruptions may therefore impede advancements in MNH outcomes, and thereby be one of the barriers to achieving the 2030 Sustainable Development Goal 3.1.

Our results suggest potential vulnerability among car-owning households: their health-seeking behaviors deviated more from baseline than those of non-car-owning households, although the difference between groups was not statistically significant (*p* = 0.064) and should be interpreted cautiously. While imprecise, this pattern is consistent with the possibility that car ownership does not necessarily ensure greater mobility during EPEs, because geographic access depends on day-to-day road passability and a well-functioning road network. Under EPEs, car-owning households may experience increased mobility costs—in time, risk, or expense—that erode their usual mobility advantage. This vulnerability may be specific to EPEs: during more modest or seasonal rainfall, car-owning households may retain their baseline advantage, while mobility constraints remain concentrated among households without motorized transport. Future research could further explore how moderate rainfall events interact with transport ownership and road conditions, particularly in settings with fragile infrastructure.

Access to high-quality services is crucial for safeguarding MNH outcomes. The quality of care provided and its geographical accessibility play a crucial role as the environment of childbirth critically influences health outcomes^10,13,27,34–36^. Unpredictable complications during labor and childbirth, such as obstructed labor or hemorrhage, can quickly become life-threatening for both woman and child, making timely access to care critical, regardless of extreme weather events^19,34,37^. International guidelines stress the importance of ensuring that women have access to a health facility within a 2-h travel time to effectively manage such emergencies and recent evidence shows that every minute counts^38,39^. Consequently, adaptive measures that ensure the access and utilization of high-quality MNH services, particularly in settings where EPEs may impede access and reduce the incentives for seeking care, are likely an effective strategy for minimizing risks associated with childbirth. Evidence at the city level suggests that higher-income cities suffer fewer adverse impacts from floods, likely due to superior infrastructure and preparedness^40,41^. These observations align with our findings on accessibility during EPEs, where road infrastructures and travel times likely mitigate the negative effects on healthcare utilization, and car owners are potentially more vulnerable as their ability to move heavily depends on a well-functioning road network.

Adaptation and preparedness strategies can influence the incentive for opting for facility-based births. Local time-varying factors, such as anticipatory behaviors prompted by local news or governmental interventions during EPEs, could modify expected health-seeking behavior. Notably, Madagascar presents a unique case in our country-specific analysis: the coefficient is positive (27.082 per 1000 live births, 95% CI − 1.635 to 55.799, *p* = 0.0645; Supplementary Fig. 12). While this estimate is not statistically significant and should be interpreted cautiously, it could reflect cyclone-related EPEs prompting different modifications in health-seeking behaviors. As the only country in our study frequently exposed to cyclones, Madagascar may have developed effective warning strategies^42^. Indeed, countries exposed to greater tropical cyclone climates display relatively smaller losses when exposed to an actual tropical cyclone event, indicating that adaptation to this climatological risk occurs^43^.

Beyond the primary findings, our work makes fundamental contributions to the literature in several ways. First, our analysis leverages a quasi-experimental design, where the identified variations within DHS clusters stem from the random timing of EPEs and births, conditional on a set of covariates and fixed effects. This approach allows for a nuanced understanding of how such weather events undermine the incentive for seeking care during critical times, such as childbirth. Second, our use of high-resolution daily precipitation data, as opposed to monthly and aggregated seasonal indicators^22,23,25,26^, highlights the fluctuating nature of accessibility on a day-to-day basis^17^. In this regard, we are the first to provide causal evidence at a daily temporal resolution and to quantify the potential decrease in facility-based births, along with spatial information on the distribution of these impacts across and within the 21 countries. Third, our use of DHS cluster fixed effects addresses issues of non-random residential sorting and treatment selection. Non-random residential sorting occurs when households and healthcare facilities strategically select locations based on the proximity of health services or market accessibility, respectively. This sorting could potentially correlate with unmeasured supply-side and demand-side determinants of birth location, such as quality of care, health status, health-seeking preferences, or income. Indeed, areas closer to the coast—correlated with higher household wealth levels and urbanization^44^—and flood-prone areas—likely inhabited by population with specific characteristics^20,45^—may be composed of population exhibiting distinct health-seeking behaviors and healthcare utilization. Selection into treatment occurs when there is a systematic residential sorting of households with specific attributes, thereby exposing themselves to different health risks, including increased susceptibility to EPEs, particularly in coastal areas. By employing DHS cluster fixed effects, we tackle these issues by leveraging within-cluster variation in EPEs and facility-based births over time.

Our study has some limitations. First, the data on the place of birth were collected only for live births and for women who survived childbirth. This characteristic of the DHS data could potentially bias our estimated exposure–response downward, as maternal mortality, stillbirths, and neonatal mortality are closely associated with facility-based births^2^.

Second, we use spatial data, which inherently carries errors that may introduce inaccuracies into our analysis. The methodology employed in the DHS includes the random displacement of cluster coordinates—up to 2 km in urban areas and up to 5 km in rural areas—to ensure respondent confidentiality. This displacement could lead to measurement errors when assessing exposure to EPEs, potentially biasing our results towards the null hypothesis^46^. Consequently, the effect sizes reported in our findings are likely underestimated.

Third, our analysis focuses on the reduced geographical accessibility induced by EPEs. We acknowledge several mechanisms, each affecting health-seeking incentives differently. Our primary focus is on local heavy rainfall that directly impairs local transportation and infrastructure (e.g., pluvial flooding)^21^. However, our approach does not fully capture fluvial floods, which result from upstream rainfall or rainfall over an entire river basin, causing rivers to overflow and affect broader regions^24,47^. We also do not account for cyclone impacts, which can induce both fluvial and coastal flooding through intense rainfall and storm surges, disrupting healthcare access over large areas^15^. This discrepancy between the location of EPEs and the location of reduced accessibility illustrates the benefit of using remote sensing technologies to accurately outline the extents of flooded/affected regions.

Fourth, we acknowledge the potential for omitted variable bias due to cluster-specific seasonality or shocks affecting both EPEs and health-seeking behaviors. Seasonal variations in workload and income, especially in agricultural settings, can alter incentives for opting for facility-based birth. During peak agricultural periods, like planting or harvesting, the demand for labor increases, raising the opportunity cost of seeking healthcare and discouraging travel to facilities. Additionally, seasonal income fluctuations may affect households’ ability to afford healthcare. These local seasonal mechanisms might correlate with the seasonal likelihood of EPEs and influence the decision to opt for non-facility births^29^. However, our placebo exercises largely address this concern, as our baseline estimate falls substantially outside the distribution of coefficients estimated from 10,000 placebo samples that randomly shuffled EPEs while preserving any unobserved local seasonal patterns around birth date (Supplementary Fig. 7).

Fifth, travel times were modeled using geolocated health facility data collected between 2012 and 2018^48^ and road network data extracted from OpenStreetMap in 2021. However, the DHS birth data span from 2010 to 2021 (Supplementary Table 17), introducing a temporal mismatch between the accessibility model and the timing of some live births, particularly those occurring in the earlier years of the study period. As a result, travel times may be underestimated for earlier live births, especially in local settings where new roads were developed after 2010. For the health facility dataset, the direction of potential error is less predictable—travel time could be either overestimated or underestimated depending on whether facilities opened or closed after the date of a given birth. In addition, the modeled surface includes only public and private not-for-profit facilities, whereas DHS respondents may have delivered in other types of facilities. Travel times in urban areas may also be underestimated due to the absence of congestion in the modeling assumptions. Separately, the road length variable used to assess the moderating role of local major road options was constructed using OSM data extracted in January 2024. The recency of this dataset may lead to upward bias in infrastructure estimates for earlier births, resulting in road length quartiles that overstate route access for some clusters during the earlier years of the study period. These limitations may affect the accuracy of both the travel time and road length measures, and introduce uncertainty into analyses involving spatial heterogeneity.

Sixth, we estimate the number of non-facility births related to EPEs assuming the same response function for all countries, which might introduce bias in our attribution analysis (Fig. 3). Our country-level analysis suggests some heterogeneous effects across countries (Supplementary Figs. 12 and 13), with pronounced vulnerability in Nigeria, Liberia, and Mali. These country-specific variations prompt the need for targeted research to better understand context-specific vulnerabilities to EPEs and their implications for MNH.

Seventh, our precipitation datasets likely underestimate the occurrences of EPEs^49,50^, and precipitation extremes show larger inter-product biases^51^. Potential biases can be addressed using multiple precipitation datasets (e.g., IMERG and MSWEP) to ensure the robustness of our findings.

Finally, future studies should investigate if the impact of EPEs extends beyond immediate diminished physical accessibility and affects healthcare delivery capacity through shortage of healthcare workers, damaged health infrastructures and lower qualities of care due to overburdened facilities and ruptured supply chains/access to blood products and referral systems. Such evidence would complement our findings on geographical accessibility and inform comprehensive climate-resilient health system planning.

Our study highlights the adverse impact of EPEs on the utilization of health facilities for childbirth in SSA, underscoring the need for targeted policy measures in vulnerable locations. The findings underscore the interplay between climate change and the incentives driving maternal health-seeking behaviors, emphasizing the importance of climate-resilient infrastructure to ensure continued access to facility-based births, and thereby safeguard MNH outcomes.

River flooding is the primary hazard in SSA, followed by surface flooding caused by extreme local rainfall, both of which threaten transport infrastructure essential for healthcare access^42^. Low- and middle-income countries are particularly vulnerable due to limited resources to plan for and maintain resilient infrastructure. This vulnerability underscores the need for targeted policy measures to prevent a widening gap between healthcare needs and utilization, particularly in the context of increasing frequency of EPEs^8,21,42^.

To address these challenges effectively, it is vital to identify women and babies at increased risk due to the lack of access to facility-based births triggered by EPE disruptions. Our study identifies the profile of regions where MNH outcomes are potentially at risk due to non-facility births caused by EPEs. In these high-risk areas, we emphasize the importance of strategic preventive and intervention measures. Recommended preventive measures include developing climate-resilient road networks (one of the key targets of SDG 9), enhancing public awareness about the risks associated with non-facility births during EPEs, and ensuring the availability of robust emergency services, early warning systems, and maternity waiting homes. Additionally, the intervention of mobile health units with advanced care in areas disrupted by EPEs could maintain skilled birth attendance when traditional access is compromised. Collectively, these strategies may contribute to a resilient healthcare ecosystem that remains accessible during EPEs, ultimately safeguarding the health and survival of MNH in SSA. By implementing these interventions, we can help ensure that the progress towards the SDG 3.1 target to significantly reduce the global maternal mortality ratio by 2030 remains on track despite the challenges posed by climate change.

## Methods

### Overview

This analysis proceeds in four steps. We first estimate the impact of EPEs on the probability of facility-based births. Second, we conduct a series of robustness checks that include alternative model specifications, tests for potential anticipation/spillover effects, placebo regression analysis, tests for recall bias and alternative definitions of EPE. In the third stage, we present additional results on the heterogeneity of effects. Finally, we use these findings to predict the number of additional non-facility births attributable to EPEs in 2015.

### Facility-based birth

We use Demographic and Health Surveys (DHS) from SSA countries to analyze facility-based births. These cross-sectional household surveys are nationally representative and have been conducted in low- and middle-income countries since the early 1980s. They provide detailed information on the health-seeking behavior and reproductive history of women of reproductive age (15–49 years). The surveys include the geographical coordinates of each cluster location (village(s) or neighborhood comprising about 25 households). To ensure participant confidentiality, the cluster coordinates are randomly displaced within the same subnational administrative region by a maximum of 2 km in urban and 5 km in rural areas, with 1% of randomly selected rural clusters displaced by up to 10 km^52^. We use the *rdhs* package in R to identify and download DHS surveys from phase 7 and onward (i.e., surveys conducted from 2013 onward) from all SSA countries, as these surveys now not only record information on children’s month and year of birth but also the day of birth, along with the geographical coordinates of the cluster^53^. We include all live births within the five years prior to each survey’s year, as the survey records place of birth information only for this recall period.

Following these inclusion criteria, the resulting dataset contained 24 surveys conducted in 21 SSA countries between 2015 and 2021, containing data on 12,948 cluster locations for 256,101 live births that occurred between 2010 and 2021 (Supplementary Table 1). The 21 countries included in our analysis are: Angola, Benin, Burundi, Cameroon, Ethiopia, Gambia, Guinea, Liberia, Madagascar, Malawi, Mali, Mauritania, Nigeria, Rwanda, Senegal, Sierra Leone, South Africa, Tanzania, Uganda, Zambia, Zimbabwe. Our primary outcome, facility-based birth, is a binary variable that takes the value of 1 if a live birth took place in a health facility (public or private hospitals, clinics, health centers, or health posts) and the value 0 if it takes place at the respondent’s home, another person’s home or another location. This information, linked to the geospatial data of DHS clusters where the woman’s household is located, forms the basis of our investigation into the influence of EPEs on facility-based births.

### Extreme precipitation events

To quantify EPEs, we use the Climate Hazards Group InfraRed Precipitation with Station (CHIRPS) dataset. CHIRPS combines satellite information and weather station data to provide greater precision for sparsely gauged locations, offering daily gridded precipitation data at a spatial resolution of 0.05 × 0.05° (approximately 5 × 5 km^2^ at the equator), extending from 1981 to the present day^54^. This dataset, preferred for its combination of both satellite and in situ measurements, offers superior ability for estimating EPEs, especially when compared to reanalysis products, which may have more limitations in accurately capturing extreme weather phenomena and convective rainfalls^55–57^.

We spatially link the DHS data with the CHIRPS precipitation data using the coordinates of the DHS clusters. We consider a three-day exposure window, spanning from two days before birth to the day of birth (inclusive), since various factors can shift the recorded day of birth and the subsequent time window in which pregnant women are presumably en route to a health facility. This time window accounts for births that happen shortly after midnight, prolonged labor durations shifting the day of birth, and longer travel times to health facilities. Additionally, EPEs can significantly alter local infrastructure and transportation costs, affecting travel conditions for extended days. Thus, the three-day exposure window allows us to capture local time-varying travel conditions caused by EPEs while pinpointing the period during which women likely began labor and initiated travel to access obstetric care.

Given the substantial variability in daily rainfall patterns across climatic regions, we anticipate that the impacts of heavy rainfall are context-specific. Populations accustomed to frequent heavy rainfall may have adapted their infrastructure and behavior accordingly, responding not to absolute amounts but to deviations from expected local rainfall amounts^47,58^. To account for this, we define EPEs using a localized approach in which we categorize them based on daily precipitation measurements exceeding the 85th percentile of the local historical distribution. This percentile threshold was selected a priori to balance identifying sufficiently extreme and relatively rare precipitation events with ensuring enough occurrences for robust statistical analysis, conditional on our comprehensive set of fixed effects. We compute the thresholds at the grid cell level, using 30 years of historical daily precipitation data (1981-2011) and including only “wet” days (≥1 mm/day) in our percentile calculations (Supplementary Fig. 3). To ensure the consistency of our findings, we also consider alternative EPE definitions using percentile thresholds ranging from the 50th to the 95th percentile (Supplementary Fig. 4) and exposure windows of 3–14 days.

### Statistical analysis

Our empirical approach exploits the data available in each DHS cluster, focusing on within-cluster variations over time rather than cross-sectional differences. By comparing live births within the same DHS cluster locations, each born at different times and exposed to varying numbers of EPE days, we effectively control for all stable, cross-sectional differences between DHS clusters. We employ the following linear probability model to estimate the effect of EPEs on facility-based births:

1 $${{{\rm{Y}}}}_{{{\rm{ivct}}}}=\,\beta {{{\rm{EPE}}}}_{{vct}}^{85{th}}+{{{\rm{\gamma }}}{{\bf{X}}}}_{{{\rm{i}}}}+{\mu }_{{{\rm{v}}}}+{\alpha }_{{{\rm{ct}}}}+{\varepsilon }_{{{\rm{ivct}}}}$$

Where Y is the binary outcome variable of child *i* born from a mother surveyed in cluster *v* in country *c* at day *t*. Our variable of interest, ${{\mathrm{EPE}}}_{{\mathrm{vct}}}^{85{\mathrm{th}}}$ is the number of days with an extreme precipitation event—that is over the 85th precipitation percentile threshold—from the day of birth to 2 days prior. ${{{\bf{X}}}}_{{{\rm{i}}}}$ is a vector of the maternal and household level covariates that might be relevant drivers of facility-based birth. The vector includes mother’s age at time of birth and highest education attainment, household wealth, parity at birth and multiples/twins^27,59–61^.

To mitigate potential biases from cross-sectional comparisons, we incorporate a rich set of fixed effects. We leverage on within-cluster variation in EPEs and facility-based births over time by adding DHS cluster fixed effects (${\mu }_{{{\rm{v}}}}$), thereby controlling for time-invariant cross-sectional differences between clusters (e.g., cultural beliefs and practices related to childbirth, altitude, proximity to the coast, local wealth) as well as for non-random residential sorting and selection into treatment induced by residential sorting^44^. Country-day-of-birth fixed effects (${\alpha }_{{{\rm{ct}}}}$) account for unobserved country-level shocks and seasonality that affect all births at a particular time (e.g., specific national holidays, Sundays, national health policies, political events, conflicts, and country-specific time trends in facility-based births over the study period), capturing both linear and non-linear patterns. This approach allows us to isolate variation in facility-based births due to exposure to EPEs from other local time-invariant and national confounders. Our key identifying assumption relies on the exogeneity of EPE timing and its effect on facility-based birth, conditional on our set of covariates and fixed effects. That is, there are no unobserved time-varying confounders that may concurrently be correlated with the likelihood of EPEs within the 3-day time window of exposure, the onset of birth, and the decision or ability to give birth in a health facility. In other words, EPEs within the three-day window are not influenced by any potential confounders related to facility-based birth. More importantly, our identification strategy exploits the quasi-random nature of both the timing of birth and EPE occurrence. Thus, variation in facility-based births can be credibly attributed to variations in EPE exposure. We use a total of 12,846 DHS cluster fixed effects and 37,767 country-day-of-birth fixed effects. The standard errors are clustered at the DHS cluster level. We weight observations using the DHS sample weights with two adjustments following previous publications^62,63^. First, we reweight the DHS sample weights by the country’s population in the year the survey was conducted to make results representative of the 21 countries in the analysis. Second, we divide the new weight by the number of survey rounds per country to account for varying numbers of surveys across countries. These adjustments ensure our estimates are representative while accounting for different survey coverage across countries.

### Sensitivity analysis

We perform multiple sensitivity checks. First, we assess the robustness of our results to the inclusion of 4582 live births with missing precipitation data, where we match them to the nearest pixel with recorded precipitation information (Supplementary Information Section 3.1). We evaluate whether our results are similar when using a (nonlinear) probit regression model. We test for anticipation and spillover effects using a distributed lag linear model, computing the number of EPE days within nine consecutive, non-overlapping 3-day intervals spanning from 14 days before to 12 days after the birth date (Supplementary Information Section 3.3). We implement a window-based randomization inference approach for placebo testing, as previously described^64^. For each birth, we randomly reassigned the sequence of EPE and non-EPE days within a ±14-day window around the birth date, preserving the total number of extreme events within each window. We repeated this permutation procedure 10,000 times, estimating equation (1) for each randomized sample to generate a distribution of placebo coefficients. This permutation preserves the seasonal average time-series structure of EPE incidence at the DHS cluster level that could be correlated with any unobserved local seasonal patterns. This allows us to test whether the timing of EPEs specifically—rather than cluster-specific seasonal patterns around birth—drives facility-based birth decisions. We test for recall bias by restricting the sample to the most recent live birth. We use alternative indices of extreme precipitation based on the Expert Team on Climate Change Detection and Indices (ETCCDI) conventions to assess the robustness of our results and to test for non-linearity^5,65^. Specifically, we consider three sets of indices: absolute-threshold precipitation indices; percentile-based precipitation exceedance totals; and absolute-threshold precipitation exceedance totals. Finally, we test the robustness of our results to the use of various sets of fixed effects.

### Extended analysis with the sustained rainfall exposure model

In addition to our primary acute (time-window) rainfall exposure model—which counts the number of EPE days within pre-birth exposure windows of 3–14 days—we conduct an extended analysis with a sustained (accumulation-window) rainfall exposure model. For each window length (3–14 days), we compute the rolling total rainfall and derive location-specific percentile thresholds (50th–95th) from the historical distribution of these rolling totals, using only wet windows (windows with total precipitation ≥1 mm). We define exposure as a binary indicator equal to 1 when the rolling total rainfall for a given window exceeds its corresponding percentile threshold (Supplementary Information Section 4.1 and Supplementary Fig. 9), and 0 otherwise. This approach offers two advantages. First, it captures the impact of multi-day events, which can cause flooding and sustained disruptions to transportation and health facility access. Second, it provides a complementary perspective to the acute (time-window) rainfall exposure model (equation (1)) by examining persistent moderate-to-heavy rainfall rather than isolated high-intensity events. We estimate the accumulation model using the same linear probability framework and fixed effects as the baseline model.

### Extended analysis by type of health facility and care at birth

Given differences in facility level, ownership, and capability, we further explore how EPEs affect the use of distinct types of delivery settings, as not all facilities are equipped to manage childbirth or complications at the level of a hospital. Specifically, we disaggregate the facility-based birth outcome by facility type—public hospitals, public lower-level facilities, and non-public facilities (all levels)—and compare each to non-facility-based births. We also examine skilled birth attendance as a separate outcome, recoded based on the highest level of medical attendant present at birth, distinguishing between skilled providers, unskilled providers, or no one.

### Heterogeneity of EPE impacts

We test for heterogeneity in the estimated average treatment effect to assess whether certain groups or places might be more vulnerable to EPEs. We do so by interacting the EPE exposure measure with relevant covariates. We examine effect heterogeneity by household wealth quintile (DHS wealth index; lowest to highest quintile), travel time to the nearest health facility and perceived access. Perceived access information is provided by the DHS survey and informs about the reason why the mother has serious problems in accessing health care. Travel time—used as a proxy for geographical accessibility to health services—was modeled by Hierink et al.^66^ using data on the locations of public and private not-for-profit health facilities collected between 2012 and 2018. To minimize attenuation bias resulting from the random displacement of DHS clusters’ coordinates, we calculated the population-weighted median travel time within each DHS buffer, spanning 2 km and 5 km for urban and rural clusters (Supplementary Information Section 7.1). We also investigated the moderating role of household car ownership and the total length of major roads within a consistent 5 km buffer around each DHS cluster (Supplementary Information Section 7.2). Major road length serves as a proxy for the availability of major road options, which may influence the degree to which EPEs disrupt access to health facilities. This fixed spatial window ensured comparability across cluster types and prevented mechanical inflation of road length measures in rural areas due to larger buffer sizes. Road network data were downloaded from OpenStreetMap on 31 January 2024. Finally, we explored heterogeneity in effects by climate zone. DHS clusters were classified as tropical, arid, or temperate using Köppen–Geiger climate zones of the period 1991-2020 (Supplementary Information Section 7.3).

### Non-facility births attributed to EPEs in 2015

Finally, we use our regression results to estimate the excess number of live births outside of health facilities (non-facility live births) attributable to EPEs and their geographical distribution for the year 2015, for which birth population estimates are available^67^. First, we determine the number of EPEs occurring from day T to T-2 for each day of 2015 within each 5 × 5 km^2^ grid cell. Next, we multiply our estimated coefficient by the exposure data to calculate the change in the probability of non-facility births per 1000 live births due to EPEs. This increase is then applied to a raster layer indicating the distribution of births on each day. For each pixel, we assume a uniform distribution of the number of births across the days of the year 2015. We then aggregate the estimated number of non-facility births to a resolution of 20 km (see Supplementary Information Section 6 for methodological details). To conclude, we investigate the cross-country heterogeneity in the excess of additional non-facility births induced by EPEs in 2015. We reproduce the same latter procedure with country-specific coefficients obtained by estimating our baseline regression separately for each country.

### Ethical approval

The study was conducted using secondary data from the DHS program, which is available on request, and thus no ethical approval was needed. The DHS received government permission and followed ethical practices, including informed consent and assurance of confidentiality. Details of the ethical review process of DHS are available on the program’s website https://dhsprogram.com/Methodology/Protecting-the-Privacy-of-DHS-Survey-Respondents.cfm.

### Reporting summary

Further information on research design is available in the Nature Portfolio Reporting Summary linked to this article.

## Supplementary information


Supplementary Information
Reporting Summary
Transparent Peer Review File


## Data Availability

The data used in this study were obtained from multiple sources. Demographic and Health Surveys (DHS) data were accessed through the DHS Program (https://dhsprogram.com/) upon registration and approval. We included all DHS surveys conducted in sub-Saharan Africa that provided (i) cluster-level geocoordinates, (ii) information on the place of delivery, and (iii) the full date of birth (day, month, and year), which were required for spatial and temporal linkage with environmental exposures. We used the Kids Recode (KR) files, which contain child-level data on birth outcomes and delivery settings, as well as linked maternal and household characteristics. Cluster geocoordinates were obtained from the corresponding DHS Geospatial Covariates files. A full list of surveys used is provided in Supplementary Table 1. Daily precipitation data were obtained from the Climate Hazards Group InfraRed Precipitation with Station (CHIRPS) dataset, version 2.0, available at https://data.chc.ucsb.edu/products/CHIRPS-2.0/. Travel time to the nearest health facility was obtained from Fleur Hierink; requests for access should be directed to the original data provider. These travel time estimates were weighted using the UN adjusted constrained population surface for 2020, available from WorldPop (https://www.worldpop.org/). Road network data were extracted from OpenStreetMap (https://www.openstreetmap.org; data accessed 31 January 2024). Gridded estimates of births for the year 2015 were obtained from WorldPop (https://www.worldpop.org/). Administrative boundary data used for map visualizations were obtained from the Database of Global Administrative Areas (GADM), version 4.1.0. Data sources and processing steps are described in the Methods and Supplementary Information.

## Code availability

All statistical analyses were conducted in R (version 4.2.2). A full list of R packages and their versions is provided in the README file accompanying the code repository. The code used for the statistical analyses and for generating the figures and tables is publicly available in the following GitHub repository: https://github.com/oumaralyba/EPEs_and_FBs and archived in Zenodo at https://doi.org/10.5281/zenodo.19257584^68^.
