# Publisher Correction: Impact of extreme precipitation events on facility-based births in 21 sub-Saharan African countries

**DOI:** 10.1038/s41467-026-76645-7

**Published:** 2026-08-26

**Authors:** Oumar Aly Ba, Fleur Hierink, Cameron Taylor, Peter M. Macharia, Lenka Beňová, Jérémy Laurent-Lucchetti, Nicolas Ray

**Affiliations:** 1 GeoHealth group, Institute of Global Health, Faculty of Medicine, University of Geneva, Geneva, Switzerland; 2 Institute for Environmental Sciences, University of Geneva, Geneva, Switzerland; 3 Department of Public Health, Institute of Tropical Medicine, Antwerp, Belgium; 4 The DHS Program, ICF, Rockville, MD USA; 5 Medicine and Health Sciences, University of Antwerp, Antwerp, Belgium; 6 Population & Health Impact Surveillance Group, Kenya Medical Research Institute-Wellcome Trust Research Programme, Nairobi, Kenya; 7 Institute of Economics and Econometrics, GSEM, University of Geneva, Geneva, Switzerland

**Keywords:** Health care economics, Health policy, Environmental health, Developing world, Environmental impact

Correction to: *Nature Communications* 10.1038/s41467-026-72547-w, published online 4 May 2026

In the version of this article initially published, the titles of Figs. 1 and 2 were interchanged, while in the Data availability section, the “Kids Recode (KR) files” was originally described as the “Children’s Recode (KR) files.” The changes are made in the HTML and PDF versions of the article.

