# Supplementary material for: Impact of extreme precipitation events on facility-based births in 21 sub-Saharan African countries
Source: Nat Commun. 2026 May 4;17:6196. doi: 10.1038/s41467-026-72547-w (PMC13369172; doi:10.1038/s41467-026-72547-w)
Supplement: Supplementary file 1 — Supplementary Information [file 41467_2026_72547_MOESM1_ESM.pdf]

Supplementary Information

Impact of extreme precipitation events on facility-based births in 21 sub-Saharan African countries

Oumar Aly Ba[1,2,3,*], Fleur Hierink[1,2], Cameron Taylor[4,5], Peter M. Macharia[3,6], Lenka Beňová[3], Jérémy Laurent-Lucchetti[2,7], and Nicolas Ray[1,2]

[1] GeoHealth group, Institute of Global Health, Faculty of Medicine, University of Geneva, Geneva, Switzerland.
[2] Institute for Environmental Sciences, University of Geneva, Geneva, Switzerland.
[3] Department of Public Health, Institute of Tropical Medicine, Antwerp, Belgium.
[4] The DHS Program, ICF, Rockville, USA.
[5] Medicine and Health Sciences, University of Antwerp, Antwerp, Belgium.
[6] Population & Health Impact Surveillance Group, Kenya Medical Research Institute-Wellcome Trust Research Programme, Nairobi, Kenya.
[7] Institute of Economics and Econometrics, GSEM, University of Geneva, Geneva, Switzerland.

*Corresponding author: Oumar Aly Ba, oumar.ba@unige.ch

Contents

1 Data and descriptive statistics **1**
1.1 DHS survey waves . . . 1
1.2 Summary statistics . . . 2
1.3 Extreme precipitation events definition . . . 9

2 Extended time-window exposure **10**

3 Sensitivity analysis **11**
3.1 Inclusion of live births with missing precipitation records . . . 11
3.2 Probit regression . . . 12
3.3 Anticipation and spillover effects . . . 13
3.4 Placebo . . . 14
3.5 Robustness to recall and selection bias . . . 15
3.6 Robustness to different extreme precipitation indices . . . 15
3.7 Robustness to different levels of fixed effects . . . 17

4 Extended analysis **18**
4.1 Sustained (accumulation-window) rainfall exposure model . . . 18
4.2 Effect of EPEs across facility types and on skilled birth . . . 22

5 Heterogeneity of EPE impacts **23**
5.1 Heterogeneity in effect of EPEs: wealth . . . 23
5.2 Heterogeneity in effect of EPEs: travel time . . . 24
5.3 Heterogeneity in effect of EPEs: perceived access, motorized vehicle . . . 25
5.4 Heterogeneity in effect of EPEs: road length . . . 26
5.5 Heterogeneity in effect of EPEs: climate zone . . . 27

6 Non-facility births attributed to extreme precipitation events in 2015 **28**
6.1 Cross-country heterogeneity in non-facility births related to EPEs . . . 30

7 Extra information and data **32**
7.1 Travel time . . . 32
7.2 Road length . . . 36
7.3 Climate zone classification of DHS clusters . . . 39

List of Figures

1 Facility-based birth share and within-cluster standard deviation . . . 6
2 85th-percentile thresholds for EPEs and within-cluster standard deviation . . . 7
3 Distribution of 85th-percentile thresholds for EPEs . . . 8
4 Extreme precipitation events definition . . . 9
5 Effect of EPEs on facility-based birth across percentile thresholds and time windows (3–14 days) . . . 10
6 EPEs and facility-based birth - anticipation and spillover effects . . . 13
7 EPEs and facility-based birth - placebo . . . 14
8 EPEs and facility-based birth - absolute-threshold precipitation indices . . . 15
9 Precipitation thresholds for acute (daily) and sustained (3–14 day) rainfall exposure models . 19
10 Estimated changes in facility-based births under acute (time-window) and sustained (accumulation-window) rainfall exposure models . . . 21

11 Estimated number of non-facility births related to EPEs in 2015 29
12 EPEs and facility-based birth - country-specific coefficients 30
13 Non-facility birth and extreme precipitation events in 2015 - country-specific coefficients . . . 31
14 Travel time distribution 34
15 Road length distribution 37
16 Climate zone classification of DHS clusters 39

List of Tables

1 Survey waves of the Demographic and Health Surveys (DHS) 1
2 Summary statistics 2
3 Summary statistics by place of delivery 3
4 Summary statistics of place of delivery by country 4
5 Summary statistics of exposure by country 5
6 EPEs and facility-based birth - including matched precipitation records 11
7 EPEs and facility-based birth - LPM and probit 12
8 EPEs and facility-based birth - recent birth 15
9 EPEs and facility-based birth - percentile-based precipitation exceedance indices and nonlinear relationship 16
10 EPEs and facility-based birth - absolute-threshold precipitation exceedance indices and nonlinear relationship 16
11 EPEs and facility-based birth - alternative set of fixed effects 17
12 EPEs and facility-based birth - across facility level 22
13 EPEs and skilled birth 22
14 EPEs and facility-based birth - wealth 23
15 EPEs and facility-based birth - travel time 24
16 EPEs and facility-based birth - perceived access and motorized vehicle 25
17 EPEs and facility-based birth - road length 26
18 EPEs and facility-based birth - climate zones 27
19 Travel time quartiles 35
20 Road length quartiles 38

1 Data and descriptive statistics

1.1 DHS survey waves

Supplementary Table 1: Survey waves of the Demographic and Health Surveys (DHS)

Country Name	Survey Year	Number of Births	Birth Range
Angola	2015-16	13,665	2010-10-24 2016-02-29
Benin	2017-18	13,232	2012-11-09 2018-02-14
Burundi	2016-17	13,135	2011-10-18 2017-02-26
Cameroon	2018	9,664	2013-07-04 2018-11-30
Ethiopia	2016	10,221	2011-01-30 2016-06-17
Ethiopia	2019	5,753	2014-03-22 2019-06-03
Gambia	2019-20	7,520	2014-12-06 2020-03-17
Guinea	2018	7,739	2013-04-02 2018-05-27
Liberia	2019-20	5,350	2014-10-28 2020-01-25
Madagascar	2021	12,265	2016-03-08 2021-07-10
Malawi	2015-16	17,286	2010-10-21 2016-02-05
Mali	2018	9,546	2013-08-12 2018-10-28
Mauritania	2019-21	11,411	2014-12-05 2021-03-13
Nigeria	2018	33,759	2013-08-15 2018-12-25
Rwanda	2019-20	8,092	2014-11-16 2020-07-13
Senegal	2018	6,487	2013-05-25 2018-11-29
Senegal	2019	5,888	2014-05-13 2019-11-21
Senegal	2017	11,631	2012-04-29 2017-12-12
Sierra Leone	2019	8,986	2014-06-02 2019-08-24
South Africa	2016	3,545	2011-07-13 2016-10-09
Tanzania	2015-16	9,788	2010-09-02 2016-02-07
Uganda	2016	15,276	2011-06-26 2016-11-21
Zambia	2018	9,730	2013-07-26 2019-01-12
Zimbabwe	2015	6,132	2010-07-09 2015-12-13

Notes: Summary statistics without Demographic and Health Surveys (DHS) sample weights.

1.2 Summary statistics

Supplementary Table 2: Summary statistics

Statistic	N	Mean	St. Dev.	Min	Pctl(25)	Pctl(75)	Max
Facility-based birth	256,101	0.6583	0.4743	0	0	1	1
N of days over 85 percentiles	256,101	0.1156	0.3702	0	0	0	3
N of days over 20 mm/day	256,101	0.1061	0.3658	0	0	0	3
Wealth	256,101	2.7065	1.3860	1	1	4	5
Education	256,101	1.8864	0.8643	1	1	3	6
Parity at birth	256,101	1.9863	0.6491	1	2	2	3
Twins	256,101	0.0350	0.1838	0	0	0	1
Age at birth	256,101	27.1421	6.8678	11.6667	21.6667	32.0000	49.5833
Perceived distance to a health facility as barrier access	248,579	0.3989	0.4897	0	0	1	1
Car/truck-owning households	256,101	0.0729	0.2600	0	0	0	1

Notes: Summary statistics without Demographic and Health Surveys (DHS) sample weights.

Supplementary Table 3: Summary statistics by place of delivery

	**Overall** 256,101 (100.00%)	**Non-facility births** 87,520 (34.17%)	**Facility-based births** 168,581 (65.83%)
N of days over 85 percentiles			
0	230,601 (100.00%)	78,779 (34.16%)	151,822 (65.84%)
1	21,727 (100.00%)	7,466 (34.36%)	14,261 (65.64%)
2	3,432 (100.00%)	1,166 (33.97%)	2,266 (66.03%)
3	341 (100.00%)	109 (31.96%)	232 (68.04%)
N of days over 20 mm/day			
0	233,427 (100.00%)	79,997 (34.27%)	153,430 (65.73%)
1	18,692 (100.00%)	6,174 (33.03%)	12,518 (66.97%)
2	3,478 (100.00%)	1,176 (33.81%)	2,302 (66.19%)
3	504 (100.00%)	173 (34.33%)	331 (65.67%)
Wealth (quintile bins)			
Quintile 1 (lowest)	66,729 (100.00%)	35,535 (53.25%)	31,194 (46.75%)
Quintile 2	57,934 (100.00%)	24,217 (41.80%)	33,717 (58.20%)
Quintile 3	51,675 (100.00%)	15,783 (30.54%)	35,892 (69.46%)
Quintile 4	43,292 (100.00%)	8,621 (19.91%)	34,671 (80.09%)
Quintile 5 (highest)	36,471 (100.00%)	3,364 (9.22%)	33,107 (90.78%)
Education			
no education	103,410 (100.00%)	51,013 (49.33%)	52,397 (50.67%)
primary	86,811 (100.00%)	25,969 (29.91%)	60,842 (70.09%)
secondary	57,449 (100.00%)	10,027 (17.45%)	47,422 (82.55%)
higher	8,426 (100.00%)	508 (6.03%)	7,918 (93.97%)
others	2 (100.00%)	1 (50.00%)	1 (50.00%)
missing	3 (100.00%)	2 (66.67%)	1 (33.33%)
Parity at birth			
No previous birth	55,723 (100.00%)	13,033 (23.39%)	42,690 (76.61%)
1 to 4 previous birth	148,164 (100.00%)	50,262 (33.92%)	97,902 (66.08%)
5 to 17 births	52,214 (100.00%)	24,225 (46.40%)	27,989 (53.60%)
Twins			
0	247,137 (100.00%)	85,065 (34.42%)	162,072 (65.58%)
1	8,964 (100.00%)	2,455 (27.39%)	6,509 (72.61%)
Travel time to the nearest health facility (quartile bins)			
Quartile 1 (lowest)	62,312 (100.00%)	11,665 (18.72%)	50,647 (81.28%)
Quartile 2	63,709 (100.00%)	17,625 (27.66%)	46,084 (72.34%)
Quartile 3	64,688 (100.00%)	25,622 (39.61%)	39,066 (60.39%)
Quartile 4 (highest)	65,261 (100.00%)	32,558 (49.89%)	32,703 (50.11%)
Perceived distance to a health facility as barrier access			
not a big problem	149,428 (100.00%)	41,012 (27.45%)	108,416 (72.55%)
big problem	99,151 (100.00%)	43,478 (43.85%)	55,673 (56.15%)
Car/truck-owning households			
no	237,428 (100.00%)	84,094 (35.42%)	153,334 (64.58%)
yes	18,673 (100.00%)	3,426 (18.35%)	15,247 (81.65%)
Road length (quartile bins)			
Quartile 1 (lowest)	63,618 (100.00%)	29,885 (46.98%)	33,733 (53.02%)
Quartile 2	64,446 (100.00%)	24,841 (38.55%)	39,605 (61.45%)
Quartile 3	64,111 (100.00%)	19,860 (30.98%)	44,251 (69.02%)
Quartile 4 (highest)	63,926 (100.00%)	12,934 (20.23%)	50,992 (79.77%)

Notes: Travel time and road length values are based on country-specific quartile groupings. Because these distributions vary across countries, the numerical values represent relative rankings within each country's distribution and do not correspond to fixed, cross-country values. For interpretation: (a) travel time quartile 1 refers to households located closest to the nearest health facility within their country, while quartile 4 includes those furthest away; (b) road length quartile 1 refers to households in neighborhoods with the lowest total length of major roads, and quartile 4 to those in areas with the highest. For country-specific distributions and threshold values used to define quartiles, see Supplementary Figs. 14 and 15 and Supplementary Tables 19 and 20. Summary statistics are reported without Demographic and Health Surveys (DHS) sample weights.

Supplementary Table 4: Summary statistics of place of delivery by country

	**Overall** 256,101 (100.00%)	**Non-facility births** 87,520 (34.17%)	**Facility-based births** 168,581 (65.83%)
Country			
Angola	13,665 (100.00%)	8,167 (59.77%)	5,498 (40.23%)
Benin	13,232 (100.00%)	2,219 (16.77%)	11,013 (83.23%)
Burundi	13,135 (100.00%)	2,121 (16.15%)	11,014 (83.85%)
Cameroon	9,664 (100.00%)	3,005 (31.09%)	6,659 (68.91%)
Ethiopia	15,974 (100.00%)	9,864 (61.75%)	6,110 (38.25%)
Gambia	7,520 (100.00%)	1,655 (22.01%)	5,865 (77.99%)
Guinea	7,739 (100.00%)	3,928 (50.76%)	3,811 (49.24%)
Liberia	5,350 (100.00%)	1,191 (22.26%)	4,159 (77.74%)
Madagascar	12,265 (100.00%)	7,755 (63.23%)	4,510 (36.77%)
Malawi	17,286 (100.00%)	1,354 (7.83%)	15,932 (92.17%)
Mali	9,546 (100.00%)	3,332 (34.90%)	6,214 (65.10%)
Mauritania	11,411 (100.00%)	3,273 (28.68%)	8,138 (71.32%)
Nigeria	33,759 (100.00%)	20,376 (60.36%)	13,383 (39.64%)
Rwanda	8,092 (100.00%)	560 (6.92%)	7,532 (93.08%)
Senegal	24,006 (100.00%)	6,150 (25.62%)	17,856 (74.38%)
Sierra Leone	8,986 (100.00%)	1,503 (16.73%)	7,483 (83.27%)
South Africa	3,545 (100.00%)	151 (4.26%)	3,394 (95.74%)
Tanzania	9,788 (100.00%)	3,739 (38.20%)	6,049 (61.80%)
Uganda	15,276 (100.00%)	4,252 (27.83%)	11,024 (72.17%)
Zambia	9,730 (100.00%)	1,682 (17.29%)	8,048 (82.71%)
Zimbabwe	6,132 (100.00%)	1,243 (20.27%)	4,889 (79.73%)

Notes: This table reports the distribution of live births by country and place of delivery. Percentages in parentheses are row percentages. Summary statistics are reported without Demographic and Health Surveys (DHS) sample weights.

Supplementary Table 5: Summary statistics of exposure by country

	Day wit EPE				
	**Overall** 256,101 (100.00%)	**0 day** 230,601 (90.04%)	**1 day** 21,727 (8.48%)	**2 days** 3,432 (1.34%)	**3 days** 341 (0.13%)
Country					
Angola	13,665 (100.00%)	12,265 (89.75%)	1,179 (8.63%)	205 (1.50%)	16 (0.12%)
Benin	13,232 (100.00%)	11,903 (89.96%)	1,204 (9.10%)	120 (0.91%)	5 (0.04%)
Burundi	13,135 (100.00%)	11,209 (85.34%)	1,620 (12.33%)	289 (2.20%)	17 (0.13%)
Cameroon	9,664 (100.00%)	8,330 (86.20%)	1,154 (11.94%)	170 (1.76%)	10 (0.10%)
Ethiopia	15,974 (100.00%)	14,883 (93.17%)	1,003 (6.28%)	84 (0.53%)	4 (0.03%)
Gambia	7,520 (100.00%)	7,011 (93.23%)	446 (5.93%)	60 (0.80%)	3 (0.04%)
Guinea	7,739 (100.00%)	7,083 (91.52%)	524 (6.77%)	113 (1.46%)	19 (0.25%)
Liberia	5,350 (100.00%)	4,571 (85.44%)	652 (12.19%)	119 (2.22%)	8 (0.15%)
Madagascar	12,265 (100.00%)	11,217 (91.46%)	822 (6.70%)	183 (1.49%)	43 (0.35%)
Malawi	17,286 (100.00%)	15,886 (91.90%)	1,133 (6.55%)	227 (1.31%)	40 (0.23%)
Mali	9,546 (100.00%)	8,717 (91.32%)	735 (7.70%)	88 (0.92%)	6 (0.06%)
Mauritania	11,411 (100.00%)	11,093 (97.21%)	304 (2.66%)	14 (0.12%)	0 (0.00%)
Nigeria	33,759 (100.00%)	29,555 (87.55%)	3,616 (10.71%)	541 (1.60%)	47 (0.14%)
Rwanda	8,092 (100.00%)	6,968 (86.11%)	942 (11.64%)	168 (2.08%)	14 (0.17%)
Senegal	24,006 (100.00%)	22,543 (93.91%)	1,262 (5.26%)	186 (0.77%)	15 (0.06%)
Sierra Leone	8,986 (100.00%)	7,766 (86.42%)	1,029 (11.45%)	167 (1.86%)	24 (0.27%)
South Africa	3,545 (100.00%)	3,332 (93.99%)	195 (5.50%)	18 (0.51%)	0 (0.00%)
Tanzania	9,788 (100.00%)	8,943 (91.37%)	698 (7.13%)	138 (1.41%)	9 (0.09%)
Uganda	15,276 (100.00%)	12,731 (83.34%)	2,174 (14.23%)	348 (2.28%)	23 (0.15%)
Zambia	9,730 (100.00%)	8,820 (90.65%)	741 (7.62%)	141 (1.45%)	28 (0.29%)
Zimbabwe	6,132 (100.00%)	5,775 (94.18%)	294 (4.79%)	53 (0.86%)	10 (0.16%)

Notes: This table reports the distribution of live births by country according to the number of days with an extreme precipitation event (EPE) in the 3-day window from the day of birth to two days prior. An EPE is defined as a daily rainfall realization over the 85th percentile of the local rainfall distribution. Percentages in parentheses are row percentages. Summary statistics are reported without Demographic and Health Surveys (DHS) sample weights.

Supplementary Fig. 1: Facility-based birth share and within-cluster standard deviation

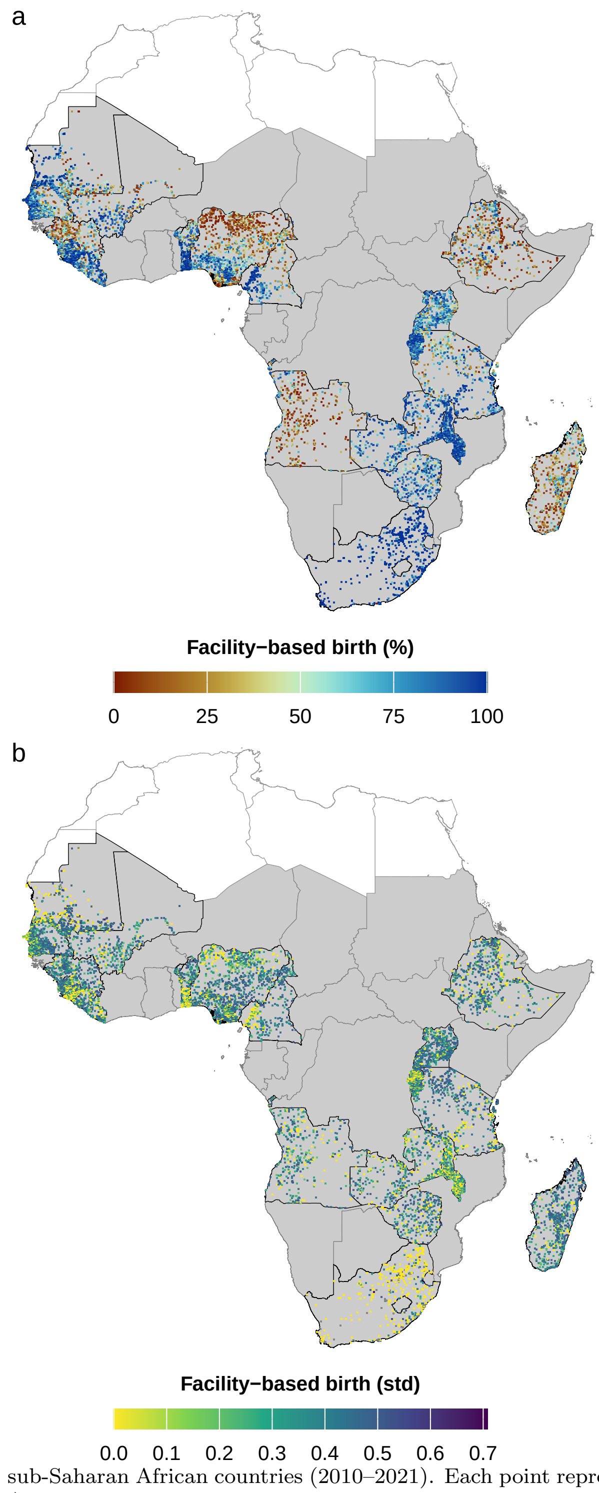

Notes: Facility-based births in 21 sub-Saharan African countries (2010–2021). Each point represents a Demographic and Health Surveys (DHS) cluster (n = 12,948). **a** Share of facility-based births in each cluster; colours indicate the percentage of live births occurring in health facilities. **b** Within-cluster variability in facility-based births, measured as the standard deviation (std) of the facility-based birth indicator across live births in each cluster; colours indicate the magnitude of variation. Sub-Saharan Africa is shown in grey and North Africa in white, with study countries outlined in black. Cluster-level shares and standard deviations are computed without DHS sampling weights. Administrative boundaries were derived from the Database of Global Administrative Areas (GADM)[1].

Supplementary Fig. 2: 85th-percentile thresholds for EPEs and within-cluster standard deviation

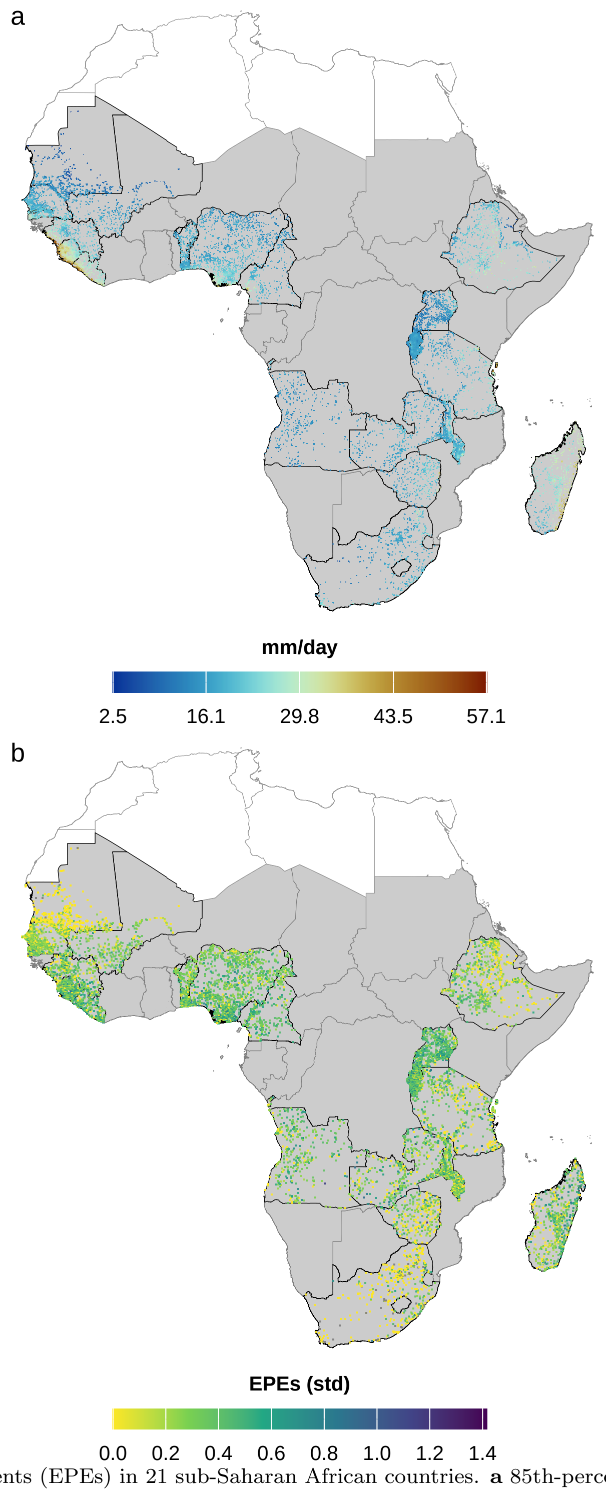

Notes: Extreme precipitation events (EPEs) in 21 sub-Saharan African countries. **a** 85th-percentile precipitation threshold (in mm/day) used to define an EPE at each 5 km × 5 km CHIRPS grid cell intersecting with at least one Demographic and Health Surveys (DHS) cluster (n = 12,948). **b** Within-cluster variability in EPEs, measured as the standard deviation of EPE days across live births in each DHS cluster; each point represents a DHS cluster and colours indicate the magnitude of variation. Sub-Saharan Africa (grey) and North Africa (white) are shown, with study countries outlined in black. Standard deviations in panel **b** are computed without DHS sampling weights. Administrative boundaries were derived from the Database of Global Administrative Areas (GADM)[1].

Supplementary Fig. 3: Distribution of 85th-percentile thresholds for EPEs

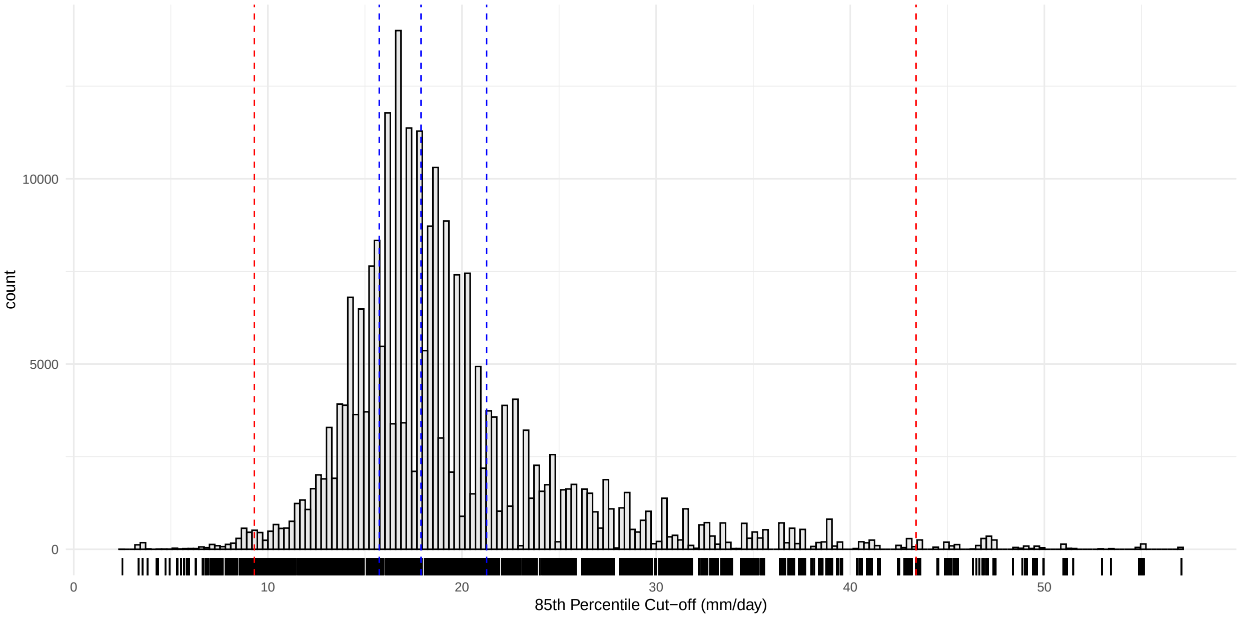

Notes: The figure shows the distribution of 85th-percentile threshold values across live births in the sample. Blue dashed lines indicate quartiles, and red dashed lines mark the 1st and 99th percentiles of the sample distribution. The distribution is computed without Demographic and Health Surveys (DHS) sample weights.

1.3 Extreme precipitation events definition

Supplementary Fig. 4: Extreme precipitation events definition

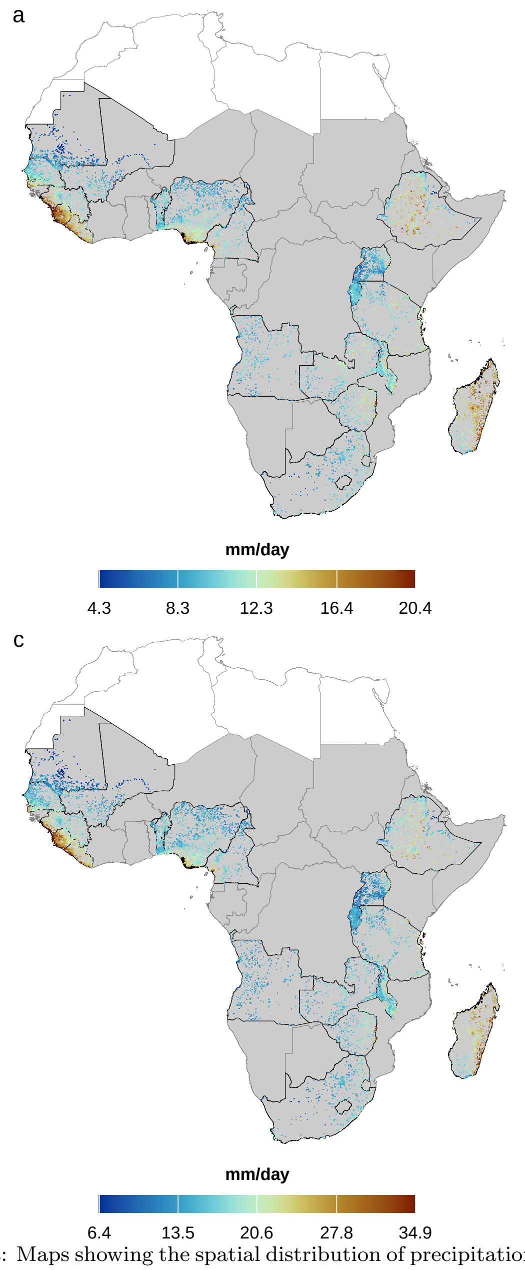

Notes: Maps showing the spatial distribution of precipitation threshold values used to define extreme precipitation events (EPEs) across different percentile cutoffs. **a** The 60th percentile precipitation threshold values. **b** The 70th percentile precipitation threshold values. **c** The 80th percentile precipitation threshold values. **d** The 90th percentile precipitation threshold values. Each grid cell corresponds to a 5 km $\times$ 5 km Climate Hazards Group InfraRed Precipitation with Station data (CHIRPS) pixel that intersects at least one Demographic and Health Surveys (DHS) cluster location (n = 12,948 clusters). Colours indicate the precipitation threshold (in mm/day) that defines an extreme event at each location across sub-Saharan Africa. For visual clarity, in each panel separately, colour scales are truncated to the 0.5th–99.5th percentile range of that panel's threshold distribution; values outside this range are mapped to the minimum or maximum colour. Administrative boundaries were derived from the Database of Global Administrative Areas (GADM)[1].

2 Extended time-window exposure

Supplementary Fig. 5: Effect of EPEs on facility-based birth across percentile thresholds and time windows (3–14 days)

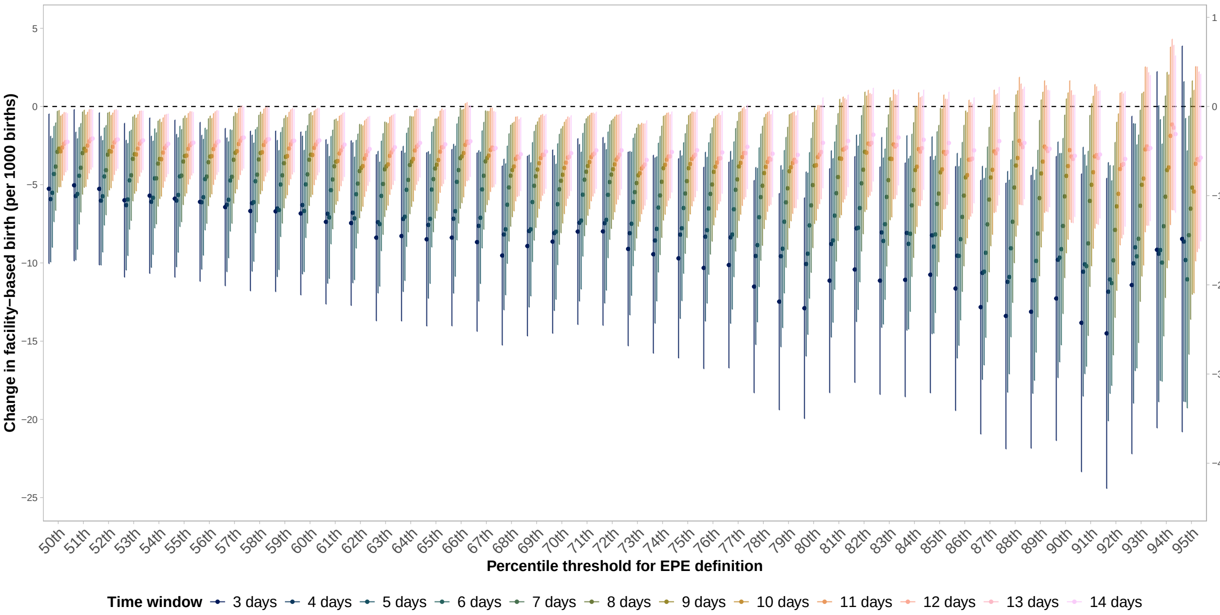

Notes: This figure extends the analysis presented in Fig. 1 of the main text by including exposure windows up to 14 days. Point estimates represent the change in facility-based birth rate (per 1000 births) for an additional day with extreme precipitation. An extreme precipitation event (EPE) is defined as a daily rainfall realization over a percentile threshold of the local rainfall distribution. All specifications include maternal and household covariates and fixed effects for Demographic and Health Surveys (DHS) clusters and country-day-of-birth. Standard errors are clustered at the DHS cluster level. Error bars indicate 95% confidence intervals around the point estimates.

3 Sensitivity analysis

3.1 Inclusion of live births with missing precipitation records

We identified 4,582 live births with missing precipitation records, all located in coastal areas where CHIRPS precipitation data pixel do not intersect with landmass. We included these records by matching them to the nearest pixel with available CHIRPS precipitation data. Although precipitation can vary greatly over short distances and potentially introduce inaccuracies [2], our main findings remained consistent when we included these matched records. Nevertheless, we excluded these matched records from the remainder of our analysis due to the potential inaccuracies.

Supplementary Table 6: EPEs and facility-based birth - including matched precipitation records

	Facility-based birth (per 1,000 births)			
	(1)	(2)	(3)	(4)
N of days over 85 percentiles	-17.342***	-10.761***	-11.079***	-10.614***
	(4.056)	(3.311)	(3.135)	(3.828)
DHS cluster FE		Yes	Yes	Yes
Day of birth FE			Yes	
Country-day of birth FE				Yes
Maternal and household controls	Yes	Yes	Yes	Yes
Sample mean	579.090	578.173	578.148	572.230
Observations	260,684	260,581	260,542	257,315
R^2	0.240	0.537	0.558	0.637

Notes: This table replicates Table 1 in the main manuscript using a sample in which live births with missing precipitation records were matched to the nearest grid cell with available precipitation data. Standard errors clustered at the Demographic and Health Surveys (DHS) cluster level are reported in parentheses. All tests are two-sided. No adjustment for multiple comparisons was made. EPEs, extreme precipitation events; Significance codes: ***P < 0.01, **P < 0.05, *P < 0.1.

3.2 Probit regression

Supplementary Table 7: EPEs and facility-based birth - LPM and probit

	LPM	Probit AME	Probit AME (bias-corrected)
	(1)	(2)	(3)
N of days over 85 percentiles	-10.753^{***}	-9.408^{***}	-9.423^{***}
	(3.852)	(0.003)	(0.003)
DHS cluster FE	Yes	Yes	Yes
Country-day of birth FE	Yes	Yes	Yes
Maternal and household controls	Yes	Yes	Yes
Sample mean	570.027	479.919	479.919
Num. obs.	252607	160765	160765

Notes: This table reports the effects of an extreme precipitation event (EPE) on the probability of facility-based birth (per 1,000 births) using a linear probability model (LPM) and probit specifications. Observations from any fixed-effect group (DHS cluster or country-day of birth) with no within-group variation in the binary outcome are dropped from the probit estimation, which explains the lower number of observations. Columns (2) and (3) present average marginal effects (AMEs) from the probit model; column (3) includes a bias correction derived by Fernández-Val and Weidner (2016)[3], implemented via the alpaca package, to address the incidental parameters problem identified by Neyman and Scott (1948)[4] in nonlinear fixed-effects models. Standard errors clustered at the DHS cluster level are reported in parentheses. All tests are two-sided. No adjustment for multiple comparisons was made. Significance codes: ***P $<$ 0.01, **P $<$ 0.05, *P $<$ 0.1.

3.3 Anticipation and spillover effects

To examine whether EPEs influence the timing of facility-based births beyond the immediate days surrounding delivery, we employed a Distributed Lag Linear Model (DLM). This approach allows us to test for three behavioral scenarios:

1. *Spillover effects*, in which EPEs that occurred well before labor might still influence delivery location decisions;
2. *Contemporaneous effects*, referring to EPEs occurring during labor or en route to a facility
3. *Anticipation effects*, where behavior may adjust in advance of forecasted EPEs (e.g., traveling to a facility early)

Specifically, we constructed a series of nine non-overlapping 3-day intervals covering the period from 14 days before to 12 days after the birth date. Within each interval, we computed the total number of days with EPE exposure, defined as precipitation exceeding the 85th percentile of the historical distribution for that DHS cluster. Each 3-day block thus represents a distinct lag or lead relative to the delivery day.

We modeled the probability of a facility-based birth using the following regression:

$$\mathrm{Y}_{ivct} = \sum_{k=-4}^{+4} \beta_k \cdot \mathrm{EPE}^{(3d)}_{vc,t+3k} + \gamma \mathbf{X}_i + \mu_v + \alpha_{ct} + \varepsilon_{ivct} \tag{1}$$

where $\mathrm{EPE}^{(3d)}_{vc,t+3k}$ is the number of EPE days in the 3-day interval ending at day $t{+}3k$ (i.e., $[t+3k-2\ ;\ t+3k]$) relative to the birth date t. $\mathbf{X}_i$ denotes maternal and household-level covariates, consistent with the main specification. μ_v and α_{ct} represent DHS cluster and country-by-day-of-birth fixed effects, respectively. ε_{ivct} is the idiosyncratic error term.

The results, illustrated in Supplementary Fig. 6 indicate that only the 3-day window ending on the day of birth (i.e., lag 0) is associated with a statistically significant decline in the likelihood of facility-based deliveries. No significant effects are observed in earlier or later intervals. These findings validate our primary specification's use of a 3-day exposure window and reinforce the plausibility of an acute, short-term effect of EPEs on delivery location decisions.

Supplementary Fig. 6: EPEs and facility-based birth - anticipation and spillover effects

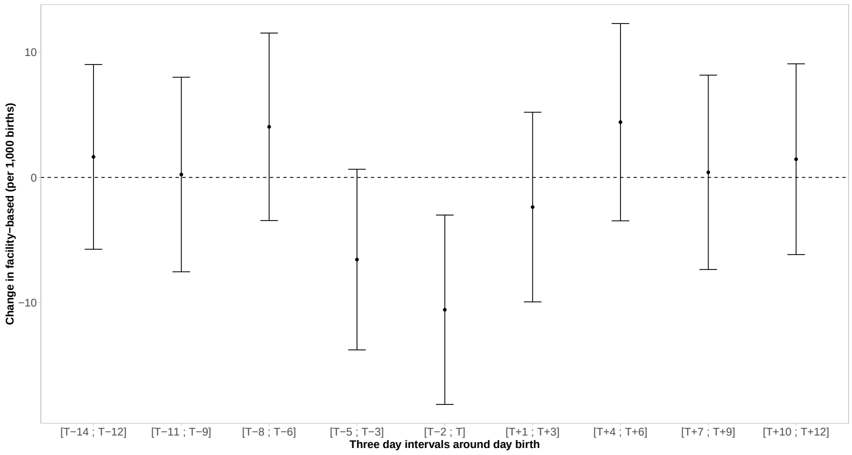

Notes: Point estimates represent the effect of an additional day of exposure to an extreme precipitation event (EPE) on the facility-based birth rate (per 1,000 births) in each non-overlapping 3-day interval from 14 days before to 12 days after birth. An EPE is defined as daily rainfall exceeding the 85th percentile of the local historical distribution. All specifications include maternal and household covariates and fixed effects for Demographic and Health Surveys (DHS) clusters and country-day-of-birth. Standard errors are clustered at the DHS cluster level. Error bars indicate 95% confidence intervals around the point estimates.

3.4 Placebo

Supplementary Fig. 7: EPEs and facility-based birth - placebo

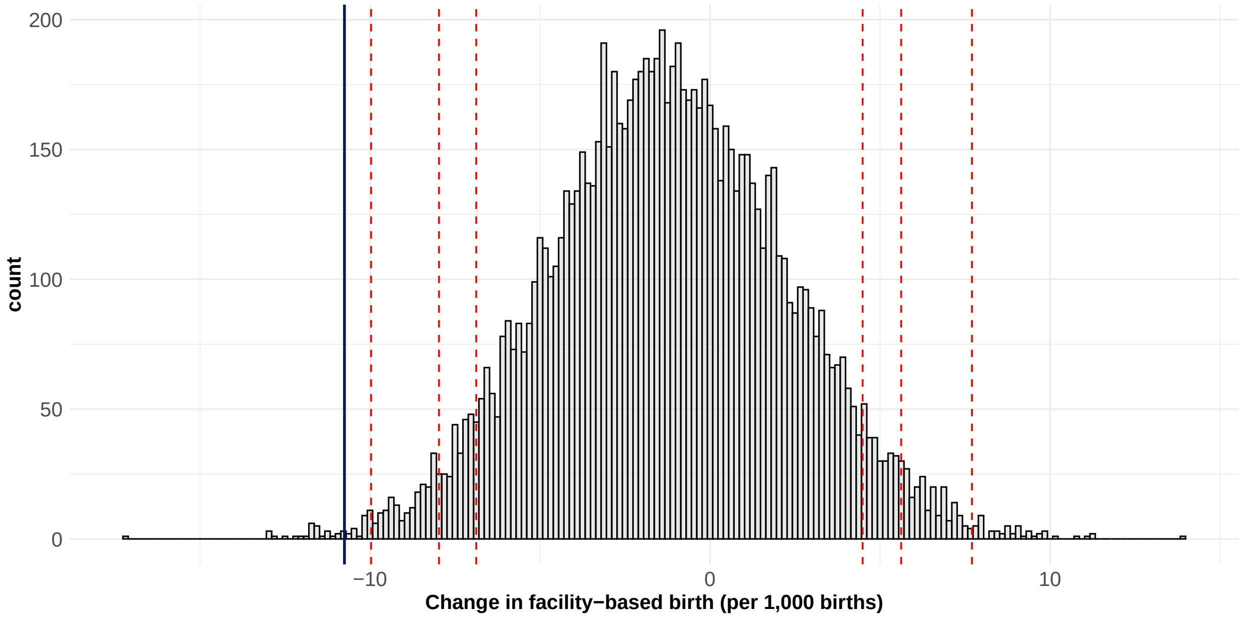

Notes: Distribution of placebo coefficients obtained from 10,000 random permutations of extreme precipitation event (EPE) timing within ±14 days of each birth date. The blue line indicates our baseline estimate (-10.753 facility-based births per 1,000 births), as estimated in equation (1). Each coefficient represents the change in facility-based births (per 1,000 births) associated with one additional day of EPE exposure in the placebo samples. Red dashed lines denote the 0.5%, 2.5%, 5%, 95%, 97.5%, and 99.5% percentiles of the placebo distribution, which help visualize the location of the true estimate relative to the distribution.

3.5 Robustness to recall and selection bias

Supplementary Table 8: EPEs and facility-based birth - recent birth

	Facility-based birth (per 1,000 births)	
	All live births	Last live birth
	(1)	(2)
N of days over 85 percentiles	-10.753***	-9.418*
	(3.852)	(4.947)
DHS cluster FE	Yes	Yes
Country-day of birth FE	Yes	Yes
Maternal and household controls	Yes	Yes
Sample mean	570.027	617.912
Observations	252,607	170,395
R^2	0.637	0.660

Notes: This table replicates Table 1 in the main manuscript using a sample restricted to recent births. EPEs denote extreme precipitation events, FE denotes fixed effects, and DHS refers to the Demographic and Health Surveys. Standard errors clustered at the DHS cluster level are reported in parentheses. All tests are two-sided. No adjustment for multiple comparisons was made. Significance codes: ***P < 0.01, **P < 0.05, *P < 0.1.

3.6 Robustness to different extreme precipitation indices

Supplementary Fig. 8: EPEs and facility-based birth - absolute-threshold precipitation indices

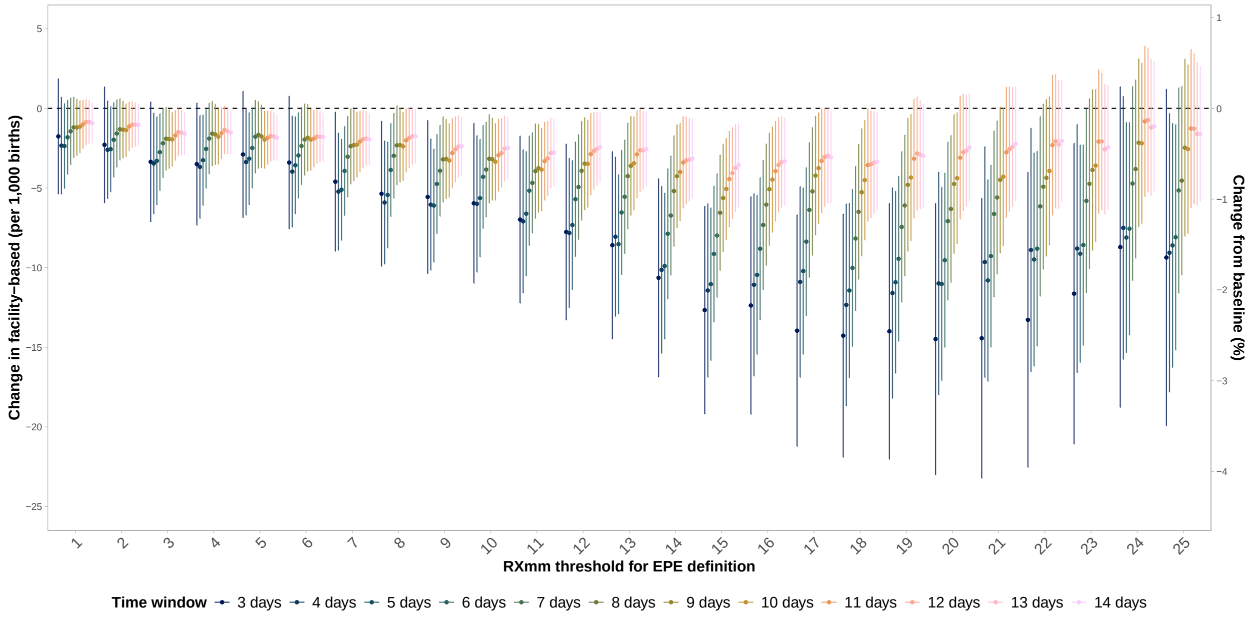

Notes: Point estimates represent the change in facility-based birth rate (per 1,000 births) for an additional day with extreme precipitation. An extreme precipitation event (EPE) is defined as a daily rainfall realization over an absolute threshold as indicated in the x-axis (from 1 mm/day to 25 mm/day). A time-window exposure ranging from 3 days to 14 days is considered for each absolute threshold. All specifications include maternal and household covariates and fixed effects for Demographic and Health Surveys (DHS) clusters and country-day-of-birth. Standard errors are clustered at the DHS cluster level. Error bars indicate 95% confidence intervals around the point estimates.

Supplementary Table 9: EPEs and facility-based birth - percentile-based precipitation exceedance indices and nonlinear relationship

	Facility-based birth (per 1,000 births)							
	R80pTOT		R85pTOT		R90pTOT		R95pTOT	
	(1)	(2)	(3)	(4)	(5)	(6)	(7)	(8)
Precipitation exceedance	-0.336***	-0.587***	-0.260**	-0.460***	-0.256**	-0.479***	-0.150	-0.341
	(0.117)	(0.170)	(0.116)	(0.170)	(0.121)	(0.181)	(0.135)	(0.207)
Precipitation exceedance^2		0.003***		0.002**		0.002**		0.002
		(0.001)		(0.001)		(0.001)		(0.001)
DHS cluster FE	Yes	Yes	Yes	Yes	Yes	Yes	Yes	Yes
Country-day of birth FE	Yes	Yes	Yes	Yes	Yes	Yes	Yes	Yes
Maternal and household controls	Yes	Yes	Yes	Yes	Yes	Yes	Yes	Yes
Sample mean	570.027	570.027	570.027	570.027	570.027	570.027	570.027	570.027
Observations	252,607	252,607	252,607	252,607	252,607	252,607	252,607	252,607
R^2	0.637	0.637	0.637	0.637	0.637	0.637	0.637	0.637

Notes: R80pTOT, R85pTOT, R90pTOT, and R95pTOT represent total daily precipitation (in mm) on days when rainfall exceeded the 80th, 85th, 90th, and 95th percentile thresholds of the local historical rainfall distribution, respectively. These indices are based on ETCCDI conventions and are calculated over the three-day exposure window from the day of birth to two days prior. Quadratic terms capture potential nonlinear effects. EPEs, extreme precipitation events; DHS, Demographic and Health Surveys. Clustered standard errors at the DHS cluster level are reported in parentheses. All tests are two-sided. No adjustment for multiple comparisons was made. Significance codes: ***P < 0.01, **P < 0.05, *P < 0.1.

Supplementary Table 10: EPEs and facility-based birth - absolute-threshold precipitation exceedance indices and nonlinear relationship

	Facility-based birth (per 1,000 births)							
	R5mmTOT		R10mmTOT		R15mmTOT		R20mmTOT	
	(1)	(2)	(3)	(4)	(5)	(6)	(7)	(8)
Precipitation exceedance	-0.300***	-0.463***	-0.307***	-0.498***	-0.369***	-0.631***	-0.320***	-0.605***
	(0.113)	(0.162)	(0.112)	(0.159)	(0.113)	(0.164)	(0.119)	(0.178)
Precipitation exceedance^2		0.002*		0.002**		0.003***		0.003***
		(0.001)		(0.001)		(0.001)		(0.001)
DHS cluster FE	Yes	Yes	Yes	Yes	Yes	Yes	Yes	Yes
Country-day of birth FE	Yes	Yes	Yes	Yes	Yes	Yes	Yes	Yes
Maternal and household controls	Yes	Yes	Yes	Yes	Yes	Yes	Yes	Yes
Sample mean	570.027	570.027	570.027	570.027	570.027	570.027	570.027	570.027
Observations	252,607	252,607	252,607	252,607	252,607	252,607	252,607	252,607
R^2	0.637	0.637	0.637	0.637	0.637	0.637	0.637	0.637

Notes: R5mmTOT, R10mmTOT, R15mmTOT, and R20mmTOT represent the total precipitation (in mm) accumulated on days where daily rainfall exceeded fixed absolute thresholds of 5 mm, 10 mm, 15 mm, and 20 mm, respectively, from the day of birth to two days prior. Quadratic terms capture potential nonlinear effects. EPEs, extreme precipitation events; DHS, Demographic and Health Surveys. Clustered standard errors at the DHS cluster level are reported in parentheses. All tests are two-sided. No adjustment for multiple comparisons was made. Significance codes: ***P < 0.01, **P < 0.05, *P < 0.1.

3.7 Robustness to different levels of fixed effects

Supplementary Table 11: EPEs and facility-based birth - alternative set of fixed effects

	Facility-based birth (per 1,000 births)				
	(1)	(2)	(3)	(4)	(5)
N of days over 85 percentiles	-10.753***	-9.158**	-4.169	-10.944***	-5.386
	(3.852)	(3.997)	(4.345)	(3.933)	(5.999)
DHS cluster FE	Yes				
ADM2 FE		Yes			
ADM2-birth month FE			Yes		
Cells FE				Yes	
Cell-birth month FE					Yes
Country-day of birth FE	Yes	Yes	Yes	Yes	Yes
Maternal and household controls	Yes	Yes	Yes	Yes	Yes
Sample mean	570.027	570.893	569.664	570.271	546.547
Observations	252,607	252,558	250,354	252,643	222,702
R^2	0.637	0.572	0.639	0.631	0.768

Notes: Specifications vary in geographic and temporal fixed effects: second-level administrative unit (ADM2), ADM2-by-birth-month, cell (5 km × 5 km spatial raster unit from the Climate Hazards Group InfraRed Precipitation with Station data (CHIRPS) dataset), and cell-by-birth-month fixed effects are used in place of Demographic and Health Surveys (DHS) cluster fixed effects in different columns. Country-day of birth fixed effects are included throughout. EPEs, extreme precipitation events. Standard errors clustered at the DHS cluster level are reported in parentheses. All tests are two-sided. No adjustment for multiple comparisons was made. Significance codes: ***P < 0.01, **P < 0.05, *P < 0.1.

4 Extended analysis

4.1 Sustained (accumulation-window) rainfall exposure model

Model definition

We extend our analysis by introducing a sustained (accumulation-window) rainfall exposure model to capture the effect of multi-day rainfall events on facility-based births. For each accumulation window (3–14 days), we compute rolling totals of daily rainfall and construct, for each DHS cluster, the historical distribution of these rolling sums. From these distributions, we derive location-specific percentile thresholds (50th–95th) and define exposure as a binary indicator equal to 1 when the rolling total exceeds its corresponding threshold and 0 otherwise. Only windows with total precipitation $\geq$1 mm are included when constructing these distributions.

This approach complements our acute (time-window) rainfall exposure model, which counts the number of EPE days in a short pre-birth window. The sustained model identifies prolonged multi-day episodes that may be less intense on any single day but are disruptive in aggregate, whereas the acute model isolates the incremental daily effect of intense rainfall.

Supplementary Fig. 9 compares rainfall thresholds under both models, using one randomly selected DHS cluster location per country to illustrate how the threshold defining an extreme event increases with the length of the accumulation window. Shorter windows (e.g. daily or 3-day totals) capture short-lived, flash-type events, while longer windows (7–14 days) highlight prolonged wet spells that can degrade road conditions and slow transport, even in the absence of overt flooding. As the accumulation period increases, the analysis shifts from capturing isolated daily extremes to capturing broader seasonal rainfall patterns that shape care access.

Supplementary Fig. 9: Precipitation thresholds for acute (daily) and sustained (3–14 day) rainfall exposure models

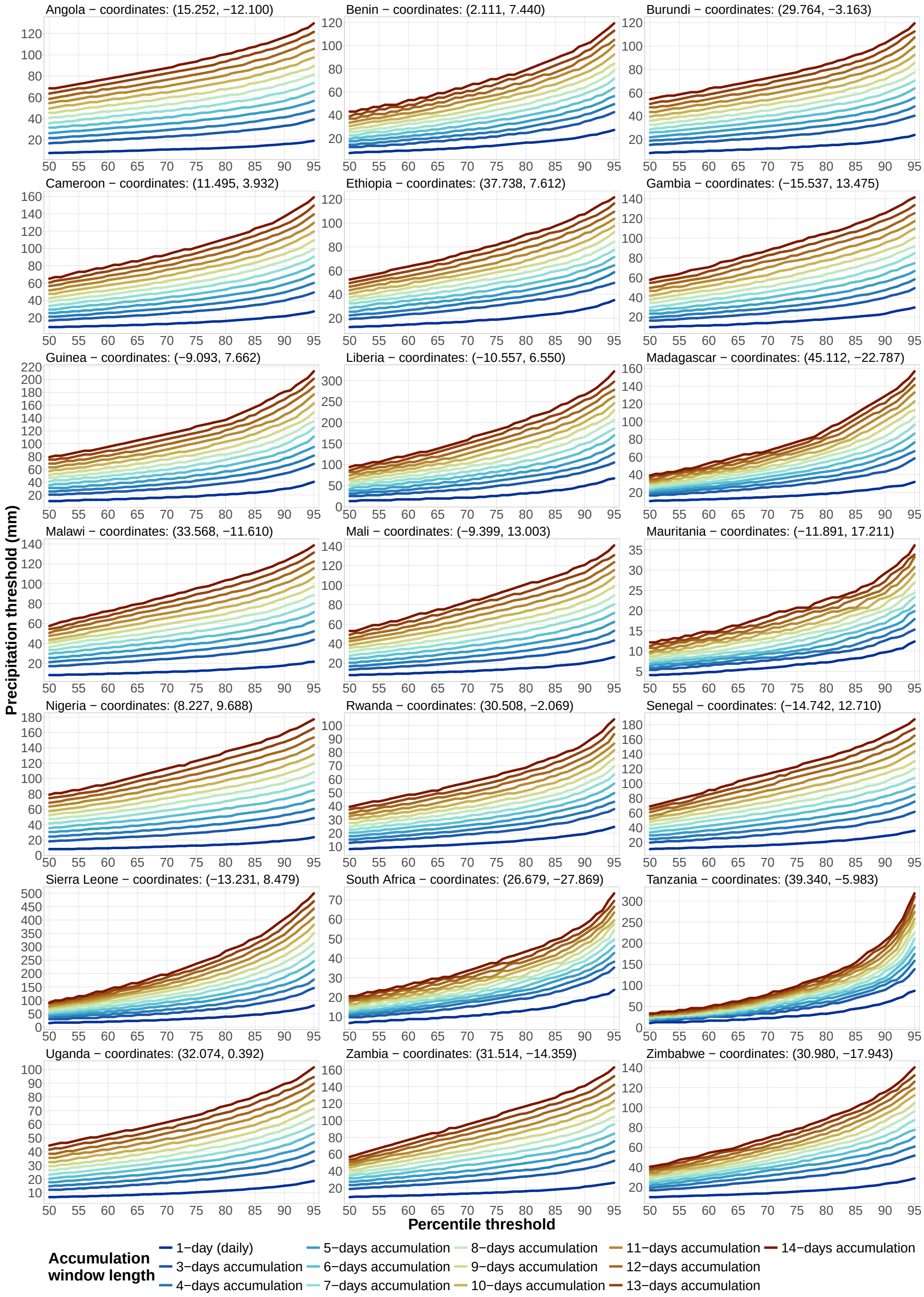

Notes: Thresholds correspond to the 50th–95th percentiles of precipitation, computed separately for each accumulation window (1-day and rolling 3 to 14 day sums) using CHIRPS daily data from 1981–2011. For all accumulation windows, only wet periods (total precipitation $\geq$ 1 mm) are retained before calculating percentiles. The 1-day thresholds correspond to the acute (time-window) exposure model, whereas the 3–14 day thresholds correspond to the sustained (accumulation-window) exposure model; the 1-day 85th-percentile threshold is the baseline EPE definition used in the main-text analyses (Table 1 and Fig. 1–3). One DHS cluster location was randomly selected per country to illustrate the relationship between accumulation length and threshold magnitude.

Model results

As described earlier in Supplementary Information Section 4.1, the sustained (accumulation-window) rainfall exposure model defines exposure as a binary indicator equal to 1 when cumulative rainfall over a given multi-day window exceeds a location-specific historical percentile threshold, and 0 otherwise. This contrasts with the acute (time-window) rainfall exposure model, which measures the incremental effect of each day of extreme precipitation. By design, the sustained model identifies disruptions that build over several consecutive days — including episodes that may not be extreme on any single day but are disruptive in aggregate — whereas the acute model isolates sudden, high-intensity daily shocks. Both models are estimated using the same linear probability framework and fixed effects as in the baseline regression model.

Supplementary Fig. 10 illustrates these complementary risk dimensions. The acute (time-window) rainfall exposure model estimates the average marginal effect per EPE day (0–N days), whereas the sustained (accumulation-window) rainfall exposure model uses a binary indicator (0/1) for whether cumulative rainfall exceeds a threshold, estimating the total impact of a multi-day event. Because of differences in variable scales and interpretations, coefficients are not directly comparable in magnitude. For instance, under the 85th percentile, each additional EPE day within a 3-day window reduces facility-based births by –10.753 per 1,000 live births (95% CI –18.304 to –3.202), while a 3-day cumulative rainfall event reduces facility-based births by –12.244 per 1,000 (95% CI –23.818 to –0.669). The larger coefficient of the accumulation-window exposure model does not imply a stronger effect but rather reflects that this metric captures the entire multi-day disruption, while the time-window model isolates the incremental linear effect per day. The sustained rainfall model further shows that moderate but sustained rainfall (50–65th percentile) can be as strongly disruptive, emphasizing the importance of accounting for chronic wet conditions.

Overall, both models reveal two complementary mechanisms: (i) acute daily shocks and (ii) sustained multi-day disruptions. The consistent negative effects across models reinforce the robustness of our findings: maternal healthcare access is compromised by both spike-like extreme events and persistent moderate-to-heavy rainfall episodes.

Supplementary Fig. 10: Estimated changes in facility-based births under acute (time-window) and sustained (accumulation-window) rainfall exposure models

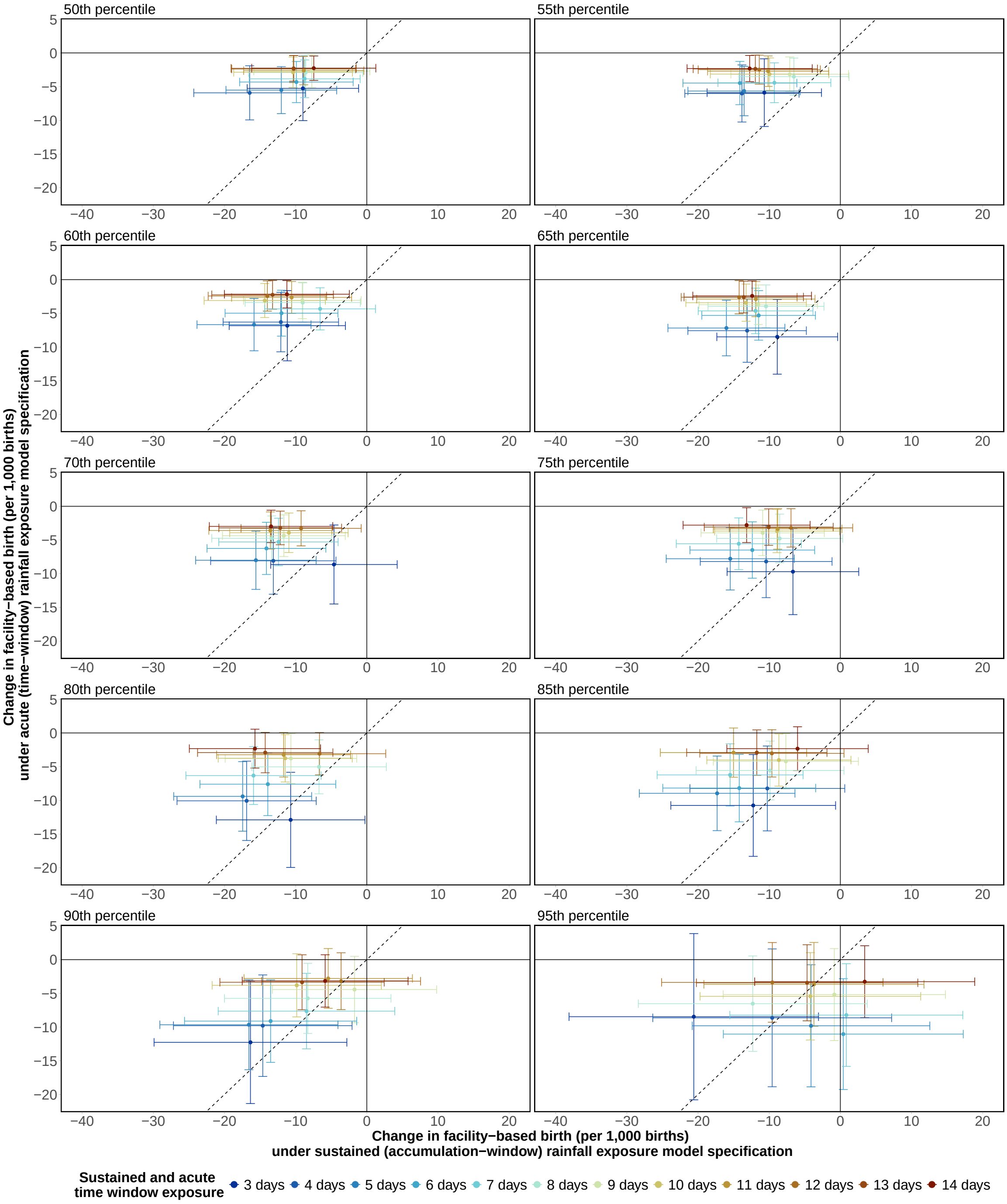

Notes: The figure shows estimated changes in facility-based births (per 1,000 live births) across percentile thresholds (50th–95th) under two exposure models: (i) the acute (time-window) rainfall exposure model, which counts the number of EPE days within a 3–14 day window (range: 0 to window length) and estimates the average marginal effect per day; and (ii) the sustained (accumulation-window) rainfall exposure model, which uses a binary indicator for whether cumulative rainfall over the same window exceeds a threshold, estimating the total event effect. Coefficients differ in scale and interpretation and are not directly comparable. The rainfall thresholds used in both models—across all combinations of percentiles and accumulation windows (1-day and rolling 3–14-day sums)—are shown in Supplementary Fig. 9, based on 10 randomly selected DHS cluster locations per country. Negative values indicate reduced facility-based births; estimates are not statistically significant if the 95% confidence interval crosses zero.

4.2 Effect of EPEs across facility types and on skilled birth

Supplementary Table 12: EPEs and facility-based birth - across facility level

	Facility-based birth (per 1,000 births)			
	Public Hospital VS Non-facility	Public Lower VS Non-facility	Nonpublic (all levels) VS Non-facility	Public Hospital VS Public Lower
	(1)	(2)	(3)	(4)
N of days over 85 percentiles	-11.755**	-5.773	-8.911**	-1.142
	(4.654)	(4.438)	(4.232)	(6.608)
DHS cluster FE	Yes	Yes	Yes	Yes
Country-day of birth FE	Yes	Yes	Yes	Yes
Maternal and household controls	Yes	Yes	Yes	Yes
Sample mean	649.083	601.028	680.219	418.435
Observations	128,296	179,803	97,143	140,738
R^2	0.751	0.654	0.756	0.687

Notes: The estimates represent the change in the probability of the binary birth-setting outcome indicated in each column heading (coded 1 for the first category and 0 for the second) for live births exposed to an additional day with an extreme precipitation event (EPE). The analysis focuses on events occurring within a three-day time window from the day of birth and the two preceding days. The exposure thus ranges from 0 (no EPE) to 3 days (EPEs occurring on all three days). An EPE is defined as a daily rainfall realization over the 85th percentile of the local rainfall distribution. Columns (1)–(4) present separate regression models for the birth-setting outcomes indicated in each column heading. All specifications include maternal and household covariates. FE denotes fixed effects, and DHS refers to the Demographic and Health Surveys. Standard errors are clustered at the DHS cluster level. All tests are two-sided. No adjustment for multiple comparisons was made. Significance codes: ***P < 0.01, **P < 0.05, *P < 0.1.

Supplementary Table 13: EPEs and skilled birth

	Skilled birth attendance (per 1,000 births)			
	Skilled VS Non-Skilled/No one	Skilled VS Non-Skilled	Skilled VS No one	Unskilled VS No one
	(1)	(2)	(3)	(4)
N of days over 85 percentiles	-10.224***	-11.962***	0.369	10.769
	(3.619)	(3.811)	(3.336)	(6.980)
DHS cluster FE	Yes	Yes	Yes	Yes
Country-day of birth FE	Yes	Yes	Yes	Yes
Maternal and household controls	Yes	Yes	Yes	Yes
Sample mean	545.082	588.903	882.755	837.134
Observations	252,607	239,773	170,386	81,184
R^2	0.661	0.660	0.722	0.472

Notes: The estimates represent the change in the probability of the binary skilled birth-attendance outcome indicated in each column heading (coded 1 for the first category and 0 for the second) for live births exposed to an additional day with an extreme precipitation event (EPE). The analysis focuses on events occurring within a three-day time window from the day of birth and the two preceding days. The exposure thus ranges from 0 (no EPE) to 3 days (EPEs occurring on all three days). An EPE is defined as a daily rainfall realization over the 85th percentile of the local rainfall distribution. Columns (1)–(4) present separate regression models for the birth-attendance outcomes indicated in each column heading. All specifications include maternal and household covariates. FE denotes fixed effects, and DHS refers to the Demographic and Health Surveys. Standard errors are clustered at the DHS cluster level. All tests are two-sided. No adjustment for multiple comparisons was made. Significance codes: ***P < 0.01, **P < 0.05, *P < 0.1.

5 Heterogeneity of EPE impacts

5.1 Heterogeneity in effect of EPEs: wealth

Supplementary Table 14: EPEs and facility-based birth - wealth

	Facility-based birth (per 1,000 births)
	(1)
N of days over 85 percentiles X Wealth Q1	−3.182
	(6.100)
N of days over 85 percentiles X Wealth Q2	−11.618*
	(6.558)
N of days over 85 percentiles X Wealth Q3	−17.212**
	(7.805)
N of days over 85 percentiles X Wealth Q4	−11.659
	(9.220)
N of days over 85 percentiles X Wealth Q5	−9.858
	(7.965)
Wealth Q2	33.411***
	(4.573)
Wealth Q3	63.854***
	(5.588)
Wealth Q4	104.645***
	(6.810)
Wealth Q5	146.570***
	(8.659)
P(Test: Wealth Q1 = Wealth Q2)	0.3208
P(Test: Wealth Q1 = Wealth Q3)	0.1366
P(Test: Wealth Q1 = Wealth Q4)	0.4260
P(Test: Wealth Q1 = Wealth Q5)	0.4862
P(Test: Wealth Q2 = Wealth Q3)	0.5639
P(Test: Wealth Q2 = Wealth Q4)	0.9970
P(Test: Wealth Q2 = Wealth Q5)	0.8591
P(Test: Wealth Q3 = Wealth Q4)	0.6362
P(Test: Wealth Q3 = Wealth Q5)	0.4918
P(Test: Wealth Q4 = Wealth Q5)	0.8780
DHS cluster FE	Yes
Country-day of birth FE	Yes
Maternal and household controls	Yes
Sample mean	570.027
Num. obs.	252607
R^2	0.637

Notes: The estimates represent the change in the probability of facility-based birth (per 1,000 births) for live births exposed to an additional day with an extreme precipitation event (EPE), allowing the effect to vary across household wealth quintiles. The analysis focuses on events occurring within a three-day time window from the day of birth and the two preceding days. The exposure thus ranges from 0 (no EPE) to 3 days (EPEs occurring on all three days). An EPE is defined as a daily rainfall realization over the 85th percentile of the local rainfall distribution. Interaction terms report the estimated effect for each wealth quintile. Q denotes quintile. Rows beginning with 'P' report two-sided Wald tests of equality between the relevant interaction terms. All specifications include maternal and household covariates. FE denotes fixed effects, and DHS refers to the Demographic and Health Surveys. Standard errors are clustered at the DHS cluster level. All tests are two-sided. No adjustment for multiple comparisons was made. Significance codes: ***P < 0.01, **P < 0.05, *P < 0.1.

5.2 Heterogeneity in effect of EPEs: travel time

Travel time is measured as a population-weighted indicator of travel time to the nearest health facility around each DHS cluster; the construction of this variable and the associated median and quartile categories is described in Supplementary Information Section 7.1.

Supplementary Table 15: EPEs and facility-based birth - travel time

	Facility-based birth (per 1,000 births)		
	(1)	(2)	(3)
N of days over 85 percentiles	−9.272		
	(6.681)		
N of days over 85 percentiles X log(Travel time)	−0.821		
	(2.689)		
N of days over 85 percentiles X Travel time (below median)		−4.482	
		(5.264)	
N of days over 85 percentiles X Travel time (above median)		−17.493***	
		(4.820)	
N of days over 85 percentiles X Travel time Q1			−3.438
			(7.516)
N of days over 85 percentiles X Travel time Q2			−6.222
			(6.732)
N of days over 85 percentiles X Travel time Q3			−20.788***
			(6.194)
N of days over 85 percentiles X Travel time Q4			−12.799*
			(6.741)
P(Test: Below median = Above median)		0.0465	
P(Test: Travel time Q1 = Travel time Q2)			0.7715
P(Test: Travel time Q1 = Travel time Q3)			0.0585
P(Test: Travel time Q1 = Travel time Q4)			0.3297
P(Test: Travel time Q2 = Travel time Q3)			0.0978
P(Test: Travel time Q2 = Travel time Q4)			0.4749
P(Test: Travel time Q3 = Travel time Q4)			0.3556
DHS cluster FE	Yes	Yes	Yes
Country-day of birth FE	Yes	Yes	Yes
Maternal and household controls	Yes	Yes	Yes
Sample mean	570.135	570.135	570.135
Num. obs.	252474	252474	252474
R^2	0.637	0.637	0.637

Notes: The estimates represent the change in the probability of facility-based birth (per 1,000 births) for live births exposed to an additional day with an extreme precipitation event (EPE), allowing the effect to vary by travel time to the nearest health facility. The analysis focuses on events occurring within a three-day time window from the day of birth and the two preceding days. The exposure thus ranges from 0 (no EPE) to 3 days (EPEs occurring on all three days). An EPE is defined as a daily rainfall realization over the 85th percentile of the local rainfall distribution. Interaction terms report the estimated effect by travel-time measure. Q denotes quartile. Rows beginning with 'P' report two-sided Wald tests of equality between the relevant interaction terms. All specifications include maternal and household covariates. FE denotes fixed effects, and DHS refers to the Demographic and Health Surveys. Standard errors are clustered at the DHS cluster level. All tests are two-sided. No adjustment for multiple comparisons was made. Significance codes: ***P < 0.01, **P < 0.05, *P < 0.1.

5.3 Heterogeneity in effect of EPEs: perceived access, motorized vehicle

Supplementary Table 16: EPEs and facility-based birth - perceived access and motorized vehicle

	Facility-based birth (per 1,000 births)		
	(1)	(2)	(3)
N of days over 85 percentiles X Distance is not a big problem	-9.664^{**}		
	(4.825)		
N of days over 85 percentiles X Distance is a big problem	-12.006^{**}		
	(5.418)		
N of days over 85 percentiles X Household dont own motorcycle/scooter		-10.168^{**}	
		(4.302)	
N of days over 85 percentiles X Household own motorcycle/scooter		-12.663^{*}	
		(7.060)	
N of days over 85 percentiles X Household dont own car/truck			-9.105^{**}
			(4.018)
N of days over 85 percentiles X Household own car/truck			-29.492^{***}
			(10.562)
Distance is a big problem	-19.538^{***}		
	(3.646)		
Household own motorcycle/scooter		10.942^{***}	
		(4.018)	
Household own car/truck			18.038^{***}
			(6.173)
P(Test: Probem = Not a problem)	0.7215		
P(Test: Not owner = Owner)		0.7500	
P(Test: Not owner = Owner)			0.0638
DHS cluster FE	Yes	Yes	Yes
Country-day of birth FE	Yes	Yes	Yes
Maternal and household controls	Yes	Yes	Yes
Sample mean	554.120	570.020	570.027
Num. obs.	245047	252606	252607
R^2	0.619	0.637	0.637

Notes: The estimates represent the change in the probability of facility-based birth (per 1,000 births) for live births exposed to an additional day with an extreme precipitation event (EPE), allowing the effect to vary by perceived distance barriers and household motorized vehicle ownership. The analysis focuses on events occurring within a three-day time window from the day of birth and the two preceding days. The exposure thus ranges from 0 (no EPE) to 3 days (EPEs occurring on all three days). An EPE is defined as a daily rainfall realization over the 85th percentile of the local rainfall distribution. Interaction terms report the estimated effect by perceived distance barriers and household motorized vehicle ownership. Rows beginning with 'P' report two-sided Wald tests of equality between the relevant interaction terms. All specifications include maternal and household covariates. FE denotes fixed effects, and DHS refers to the Demographic and Health Surveys. Standard errors are clustered at the DHS cluster level. All tests are two-sided. No adjustment for multiple comparisons was made. Significance codes: ***P < 0.01, **P < 0.05, *P < 0.1.

5.4 Heterogeneity in effect of EPEs: road length

Road length is measured as the total length of major roads within 5 km of each DHS cluster; the construction of this variable and the associated median and quartile categories is described in Supplementary Information Section 7.2.

Supplementary Table 17: EPEs and facility-based birth - road length

	Facility-based birth (per 1,000 births)		
	(1)	(2)	(3)
N of days over 85 percentiles	-25.835^{**}		
	(11.868)		
N of days over 85 percentiles X log(0.01+Road Length)	1.418		
	(1.087)		
N of days over 85 percentiles X Road Length (below median)		-16.755^{***}	
		(4.906)	
N of days over 85 percentiles X Road Length (above median)		-5.506	
		(5.145)	
N of days over 85 percentiles X Road length Q1			-14.287^{**}
			(6.767)
N of days over 85 percentiles X Road length Q2			-19.193^{***}
			(6.473)
N of days over 85 percentiles X Road length Q3			-6.681
			(7.200)
N of days over 85 percentiles X Road length Q4			-4.321
			(6.729)
P(Test: Below median = Above median)		0.0838	
P(Test: Road length Q1 = Road length Q2)			0.5805
P(Test: Road length Q1 = Road length Q3)			0.4179
P(Test: Road length Q1 = Road length Q4)			0.2672
P(Test: Road length Q2 = Road length Q3)			0.1780
P(Test: Road length Q2 = Road length Q4)			0.0988
P(Test: Road length Q3 = Road length Q4)			0.8019
DHS sample cluster FE	Yes	Yes	Yes
Country-day of birth FE	Yes	Yes	Yes
Maternal and household controls	Yes	Yes	Yes
Sample mean	570.027	570.027	570.027
Num. obs.	252607	252607	252607
R^2	0.637	0.637	0.637

Notes: The estimates represent the change in the probability of facility-based birth (per 1,000 births) for live births exposed to an additional day with an extreme precipitation event (EPE), allowing the effect to vary by road length around the DHS cluster. The analysis focuses on events occurring within a three-day time window from the day of birth and the two preceding days. The exposure thus ranges from 0 (no EPE) to 3 days (EPEs occurring on all three days). An EPE is defined as a daily rainfall realization over the 85th percentile of the local rainfall distribution. Interaction terms report the estimated effect by road-length measure. Q denotes quartile. Rows beginning with 'P' report two-sided Wald tests of equality between the relevant interaction terms. All specifications include maternal and household covariates. FE denotes fixed effects, and DHS refers to the Demographic and Health Surveys. Standard errors are clustered at the DHS cluster level. All tests are two-sided. No adjustment for multiple comparisons was made. Significance codes: ***P < 0.01, **P < 0.05, *P < 0.1.

5.5 Heterogeneity in effect of EPEs: climate zone

We explored whether EPE impacts varied by climate zone. Each DHS cluster was classified as tropical, arid, or temperate using the Köppen–Geiger system described in Supplementary Information Section 7.3.

We estimated interaction models between climate-zone indicators and two rainfall exposure definitions: (i) the acute (time-window) model, defined as the number of EPE days (>85th percentile) in the 3-day window preceding and including the day of birth; and (ii) the sustained (accumulation-window) model, a binary indicator for whether total rainfall over 5 days exceeded the local 85th percentile of historical 5-day totals.

All models included the same covariates and fixed effects as the baseline, with standard errors clustered at the DHS-cluster level. Baseline effects for climate-zone groups are omitted because they are absorbed by cluster fixed effects. For the sustained model, we retained the 85th percentile threshold from the baseline model and used the 5-day window, which produced the strongest effect at this threshold within the sustained specification (Supplementary Fig. 10).

Supplementary Table 18 show that in the acute model, tropical zones experienced a decline of -14.498 facility-based births per 1,000 live births (95% CI -23.374, -5.621; $p < 0.01$), while estimates for arid (0.856; 95% CI -16.015, 17.726; $p = 0.920$) and temperate zones (-1.701; 95% CI -17.504, 14.103; $p = 0.832$) were not statistically significant. The difference between tropical and arid zones was not statistically significant ($p = 0.098$), and the tropical–temperate difference was similarly not significant ($p = 0.154$). In the sustained model, tropical zones showed a larger decline of -21.613 per 1,000 (95% CI -34.363, -8.862; $p < 0.01$), compared with smaller and non-significant reductions in arid (-10.426; 95% CI -34.679, 13.827; $p = 0.399$) and temperate (-5.955; 95% CI -29.452, 17.542; $p = 0.619$). No pairwise differences between zones were statistically significant ($p > 0.10$).

Although differences were not consistently significant, the pattern of larger tropical-zone estimates is suggestive and warrants further investigation. The spatial distribution of DHS clusters across climate zones is shown in Supplementary Fig. 16.

Supplementary Table 18: EPEs and facility-based birth - climate zones

	Facility-based birth (per 1,000 births)	
	(1)	(2)
N of days over 85 percentiles X Tropical	-14.498^{***}	
	(4.529)	
N of days over 85 percentiles X Arid	0.856	
	(8.607)	
N of days over 85 percentiles X Temperate	-1.701	
	(8.063)	
EPE 5-days spell (85th percentiles) X Tropical		-21.613^{***}
		(6.505)
EPE 5-days spell (85th percentiles) X Arid		-10.426
		(12.373)
EPE 5-days spell (85th percentiles) X Temperate		-5.955
		(11.988)
P(Test: Tropical = Arid)	0.0984	0.4071
P(Test: Tropical = Temperate)	0.1544	0.2283
P(Test: Arid = Temperate)	0.8245	0.7888
DHS cluster FE	Yes	Yes
Country-day of birth FE	Yes	Yes
Maternal and household controls	Yes	Yes
Sample mean	570.027	570.027
Num. obs.	252607	252607
R^2	0.637	0.637

Notes: Column (1) reports results from the acute (time-window) rainfall exposure model, defined as the number of days with an extreme precipitation event (EPE) in the 3-day window preceding and including the day of birth. Column (2) reports results from the sustained (accumulation-window) rainfall exposure model, defined as a binary indicator equal to 1 if total rainfall over the 5-day window exceeded the local 85th percentile of historical 5-day totals. Rows beginning with P report two-sided Wald tests of equality between the relevant interaction terms. DHS, Demographic and Health Surveys. Standard errors are clustered at the DHS cluster level. All tests are two-sided. No adjustment for multiple comparisons was made. Significance codes: ***P < 0.01, **P < 0.05, *P < 0.1.

6 Non-facility births attributed to extreme precipitation events in 2015

To estimate the increase in non-facility births attributable to extreme precipitation events (EPEs) during 2015, we first acquired gridded maps of births that occurred in 2015 for each country from the WorldPop database [5], with a spatial resolution of 1 km x 1 km. We assumed a uniform daily distribution of births throughout the year as follows:

$$N_{it} = \frac{\text{Number of births in 2015}}{\text{365 days}} \tag{2}$$

where N_{it} represents the number of births on day t for each 1 km x 1 km pixel i.

Second, for each day t of the year 2015 and within each 5km x5 km grid of the CHIRPS precipitation dataset, we identified the number of EPEs that occurred from day t to day $t-2$. The parameter $\hat{\beta}$, estimated from our baseline regression model (table 1, column 4), was then multiplied to the EPE data for each pixel-day to quantify the shift in the probability of birth location decisions due to these events as follows:

$$\Delta\text{HBR}_{it} = \hat{\beta} \times \sum_{l=t-2}^{t} \text{EPE}_{il}, \tag{3}$$

where ΔHBR_{it} is the estimated change in the probability of non-facility birth on day t for each 5 km x 5 km pixel i owing to the sum of exposure to EPEs that occurred from birth date t to two days prior $t-2$.

Third, we resampled ΔHBR_{it} to align it with the spatial resolution of N_{it} using nearest neighbour interpolation. This enabled us to estimate the number of non-facility births attributable to EPE for each pixel-day as follows:

$$\text{HB}_{it} = N_{it} \times \Delta\text{HBR}_{it}, \tag{4}$$

Finally, we computed the total excess of non-facility births induced by all EPEs of the year 2015 for each pixel i as:

$$\text{HB}_{i} = \sum_{t=1}^{365} \text{HB}_{it}. \tag{5}$$

where t ranges from the first to the last day of 2015.

For better visualization of the influence of EPEs on non-facility births across broader geographic regions, we further aggregated the pixel-level data from 1 km to 20 km resolution and also expressed the in 1,000 live births.

Supplementary Fig. 11: Estimated number of non-facility births related to EPEs in 2015

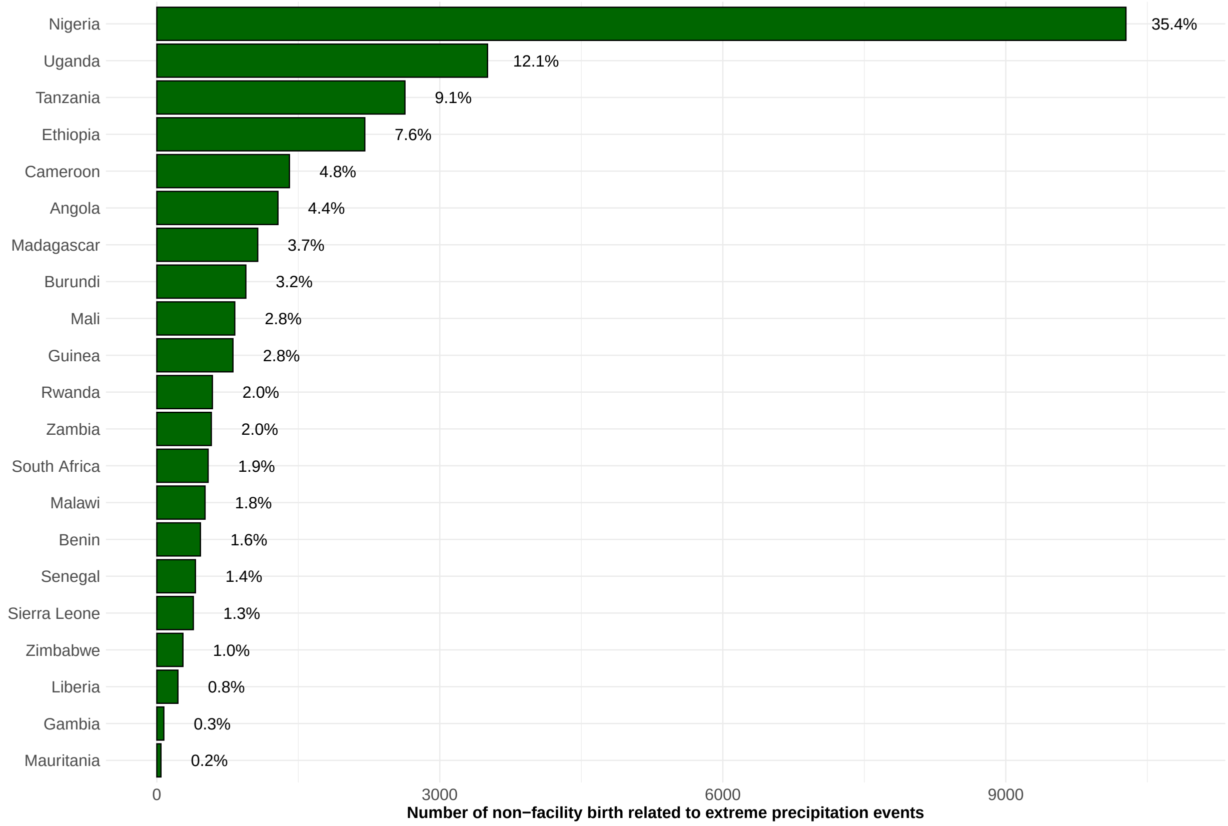

Notes: Estimated number of non-facility births in 2015 attributable to extreme precipitation events (EPEs), defined as daily rainfall realizations above the 85th percentile of the local historical rainfall distribution within a 3-day window from the day of birth to two days prior. Estimates combine 2015 WorldPop birth counts, 2015 daily EPE exposures, and the baseline regression coefficient (Table 1, Column 4), assuming births are uniformly distributed across the year. Percentages indicate the share of total non-facility births attributable to EPEs.

6.1 Cross-country heterogeneity in non-facility births related to EPEs

We explored the stability of excess of non-facility births using country-specific coefficients - obtained by estimating our baseline regression separately for each country (Supplementary Fig. 12) - instead of single main parameter $\hat{\beta}_1$. We then follow the same procedure described in Supplementary Information Section 6.

Supplementary Fig. 12: EPEs and facility-based birth - country-specific coefficients

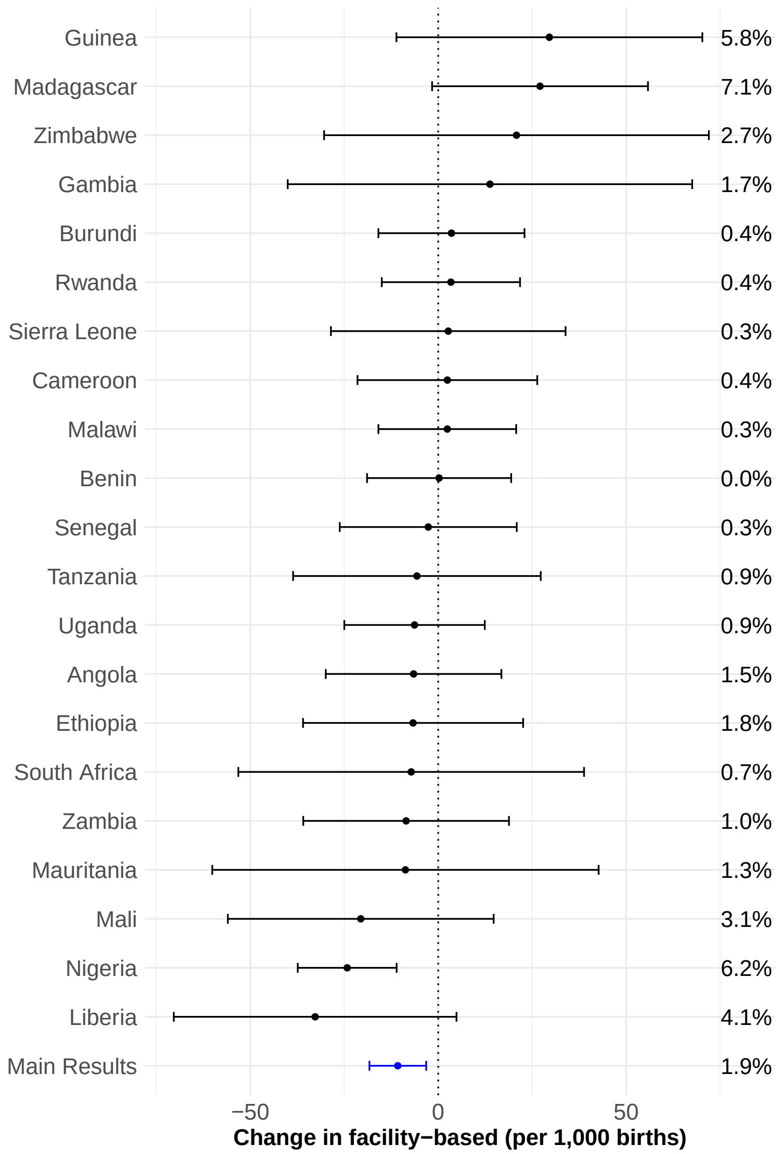

Notes: Point estimates show the change in facility-based birth rate (per 1,000 live births) for an additional day with an extreme precipitation event (EPE), defined as daily rainfall exceeding the 85th percentile of the local historical rainfall distribution, within the 3-day window spanning the day of birth and the two preceding days (0–3 days of exposure). Results are from the baseline regression model estimated separately for each country; percentages indicate change relative to the national mean facility-based birth rate. Models include maternal and household covariates, plus Demographic and Health Surveys (DHS) cluster and country-day-of-birth fixed effects (FE). Standard errors are clustered at the DHS cluster level. Error bars show 95% confidence intervals around the point estimates. The blue marker shows the baseline estimate from Table 1 using all countries combined in a single model.

Supplementary Fig. 13: Non-facility birth and extreme precipitation events in 2015 - country-specific coefficients

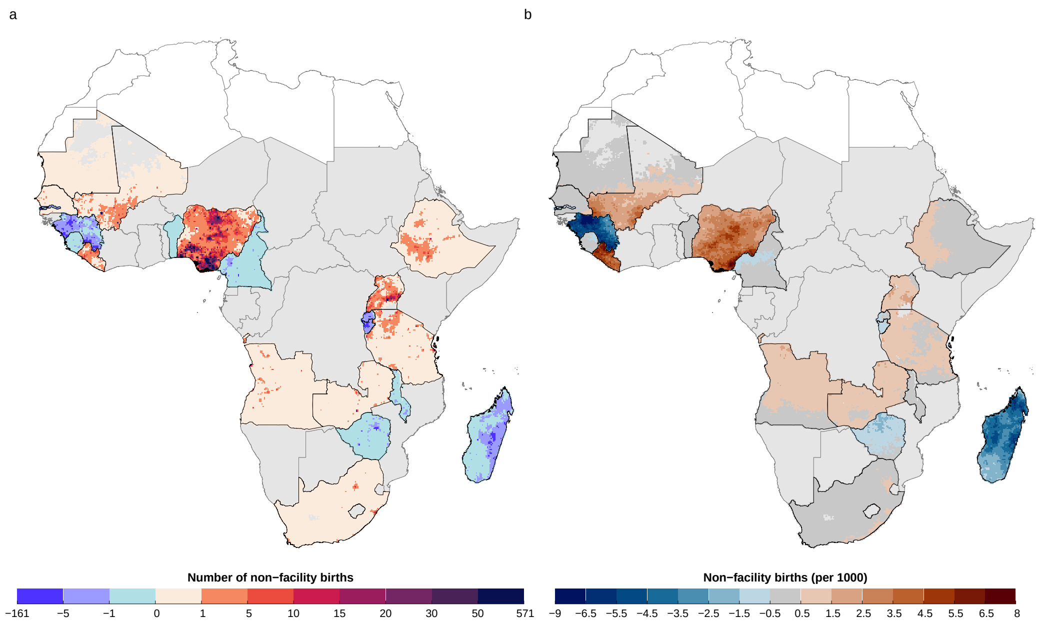

Notes: **a** Number of non-facility births attributable to extreme precipitation events (EPEs) in 2015 per 20 km x 20 km cell. **b** Number of non-facility births per 1000 live births attributable to EPEs in 2015 per 20 km x 20 km cell. Excess of non-facility births are computed using country-specific coefficients from Supplementary Fig. 12. Administrative boundaries were derived from the Database of Global Administrative Areas (GADM)[1].

7 Extra information and data

7.1 Travel time

In this section, we detail on the construction of the *travel time* variable, weighted using high-resolution population rasters, as well as the *travel time median dummy* and *travel time quartile bins* used in the regression analysis.

Data and sources

Travel time raster data

We use a travel time dataset of 100 m x 100 m resolution, generated by Hierink and colleagues [6]. This dataset is based on country-specific travel scenarios that make assumptions about travel modes (e.g., walking, motorized) and average speeds on different road type and off-road travel (based on different landcover category). The dataset combines high-resolution raster data with information on the distribution of health facilities from Maina and colleagues [7].

Population data

We use the constrained version of population data provided by WorldPop for the year 2020 [8]. The dataset provides high-resolution estimates of population counts, adjusted to match official United Nations population estimates and distributed across a 100-meter grid. WorldPop constrained the estimate of population distribution to building footprints or settlement feature data derived from satellite imagery.

Methodological details

We combine travel time information and population data with DHS cluster locations to reflect health service accessibility from the perspective of the population around the DHS clusters. By using population-weighted travel time calculations based on the WorldPop constrained population data, we ensured that the travel times are both geographically precise and demographically representative of local population. This approach acknowledges that not all areas within a DHS cluster buffer zone contribute equally to the overall travel time, with more populous areas exerting a greater influence on the weighted median calculations.

Travel time

The *travel time* variable is the median travel time to the nearest health facility. The median is calculated considering the population count around each DHS respondent location, reflecting health service accessibility for most people in the surrounding area. Areas with higher populations have a greater influence on the median calculation than less populated areas. The steps below outline this procedure.

1. Creation of spatial buffers around DHS cluster's locations. We considered a 2 kilometers for urban settings and 5 kilometers for rural ones to define the analysis area.
2. Application of the country-specific projection system to the DHS, travel time and population data to ensure geographic accuracy.
3. Extraction of travel time value and population count for all pixel falling within these buffers, along with the fraction of the pixel covered by the buffer for each pixel.
4. Computation of the median travel time by weighting each travel time value according to the population count of its area. This is achieved by multiplying the travel time of each cell by the population count in that cell and the fraction of that cell covered by the buffer before calculating the median of the buffer zone. This method ensures that areas with more people contribute more to the definition of the

median value, making it more representative of the surrounding population's overall accessibility. For buffers where population data indicates a population of 0 for all pixels within the buffer, we assigned a default population weight of 1 to all pixels.

The final *travel time* is the median travel time to the nearest health facility, adjusted for population distribution located within the DHS cluster buffer zones.

Travel time - median dummy

We also created a binary version of the *travel time* to distinguish between live births based on whether their *travel time* exceeds the national median value. This variable facilitates analysis by comparing live births with varying levels of access, defined by their relative position to the national median *travel time* of live births. We carried out the following steps:

1. Calculation of the national median value of *travel time* across live births within each country, establishing a benchmark for comparison.
2. Classification of live births into two groups: those with a value above and below the country-specific median.

Travel time - quartile bins

We also assign live births to quartiles based on their *travel time* within their respective countries. This categorization into quartiles allows for analysis across four distinct levels of health service access. We carried out the following steps:

1. Identification of 0%, 25%, 50%, 75%, and 100% percentile values of *travel time* from the distribution of live births in each country to define quartile boundaries.
2. Assignment of each live birth's *travel time* to one of the four quartile group based on these country-specific thresholds, thereby ranking each live birth's *travel time* within a quartile system.

Supplementary Fig. 14 and Supplementary Table 19 report the distribution of *travel time* across live births and country-specific quartile and median thresholds.

Supplementary Fig. 14: Travel time distribution

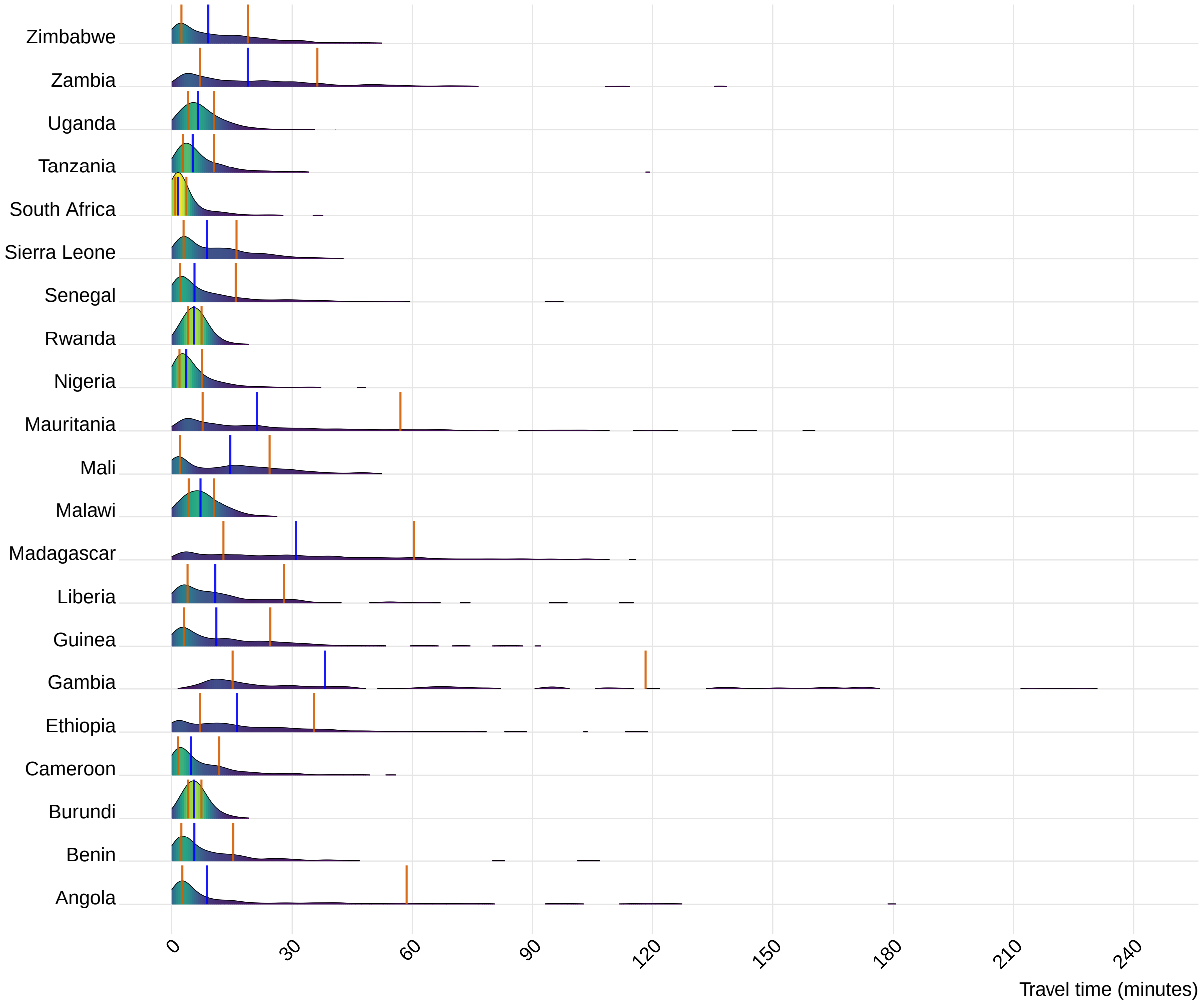

Notes: This figure shows the distribution of estimated travel time (in minutes) to the nearest health facility for each country, based on the unweighted Demographic and Health Surveys (DHS) sample of live births. Country-specific quartile groupings are derived from the distribution of travel times among live births within each country. Vertical lines indicate the 25th (orange), 50th (median; blue), and 75th (orange) percentiles used to define quartiles. For visualization purposes, the top 1% of travel time values are excluded. Corresponding quartile thresholds for each country are reported in Supplementary Table 19.

Supplementary Table 19: Travel time quartiles

Country	Q0	Q25	Q50	Q75	Q100
Angola	0.766	2.685	8.800	58.579	1,326.862
Benin	0.766	2.441	5.673	15.351	186.939
Burundi	0.847	4.154	5.614	7.437	19.030
Cameroon	0.636	1.644	4.809	11.864	149.906
Ethiopia	0.221	7.076	16.264	35.574	423.932
Gambia	4.594	15.188	38.279	118.244	307.410
Guinea	0.964	3.136	11.142	24.564	343.165
Liberia	0.799	3.986	10.880	27.951	626.515
Madagascar	1.303	12.903	30.990	60.454	650.953
Malawi	1.079	4.275	7.205	10.514	42.786
Mali	0.556	2.142	14.614	24.381	378.388
Mauritania	1.235	7.736	21.272	57.039	2,629.616
Nigeria	0.170	1.978	3.671	7.597	1,018.248
Rwanda	0.936	4.071	5.643	7.470	53.152
Senegal	0.509	2.152	5.711	15.965	236.536
Sierra Leone	0.466	3.000	8.833	16.160	91.438
South Africa	0.374	0.949	1.669	3.697	150.925
Tanzania	0.658	2.835	5.266	10.526	298.381
Uganda	0.733	4.115	6.621	10.585	217.249
Zambia	1.064	7.091	18.959	36.385	584.514
Zimbabwe	0.666	2.464	9.141	19.061	160.989

Notes: This table reports the country-specific percentile values (Q0, Q25, Q50, Q75, Q100) of estimated travel time (in minutes) to the nearest health facility, calculated from the unweighted Demographic and Health Surveys (DHS) sample of live births. These percentiles are used to define within-country quartile groupings, where quartile 1 corresponds to households closest to a facility and quartile 4 to those furthest away.

7.2 Road length

In this section, we provide details on the construction of the *road length* variable, as well as the *road length median dummy* and *road length quartile bins* used in the regression analysis.

Data and sources

Road network data from OpenStreetMap

The road network dataset in vector format was extracted from OpenStreetMap (OSM) on January 31, 2024 using the `osmextract` package in R. We filtered for all line shapefiles that are roads by using the OSM tag "highways" and selecting the following road categories: "motorway", "motorway_link", "trunk", "trunk_link", "primary", "primary_link", "secondary", "secondary_link", "tertiary", "tertiary_link", and "unclassified". We considered these types of highway as they are important/major roads and therefore less likely to be systematically underrepresented in areas with limited mapping coverage.

Methodological details

Road length calculation

We computed the total road length available around each DHS cluster's location to proxy for the quantity of possible routes. The road length is computed as follow:

1. Creation of spatial buffers around DHS cluster's location. We considered a 5 kilometers buffer for both urban and rural settings.
2. Clipping of roads located inside the buffer of any DHS cluster.
3. Calculation of the road length of each road segment.
4. Calculation of the total length of roads falling within the buffer of each DHS cluster.

Road length - median dummy

We also created a binary *road length* variable to distinguish whether access to roads is above or below the national median road length of the DHS sample of live births. This classification facilitates comparisons within and across countries, highlighting disparities in route options. We carried out the following steps:

1. Calculation of the national median value of *road length* across live births within each country, establishing a benchmark for comparison.
2. Classification of live births into two groups: those with a value above and below the country-specific median. This binary variable thus indicates relative accessibility to possible route options, thus providing proxy of infrastructure adequacy relative to national standards.

Road length - quartile bins

We also assign live births to quartiles based on the distribution *road length* in their respective countries. This categorization into quartiles allows for analysis across four distinct levels of access top route options. We carried out the following steps:

1. Identification of 0%, 25%, 50%, 75%, and 100% percentile values of *road length* from the distribution of live births in each country to define quartile boundaries.
2. Assignment of each live birth's *road length* to one of the four quartile groups based on these country-specific thresholds, thereby ranking each live birth's *road length* within a quartile system.

Supplementary Fig. 15 and Supplementary Table 20 report the distribution *road length* across live births and country-specific quartile and median thresholds.

Supplementary Fig. 15: Road length distribution

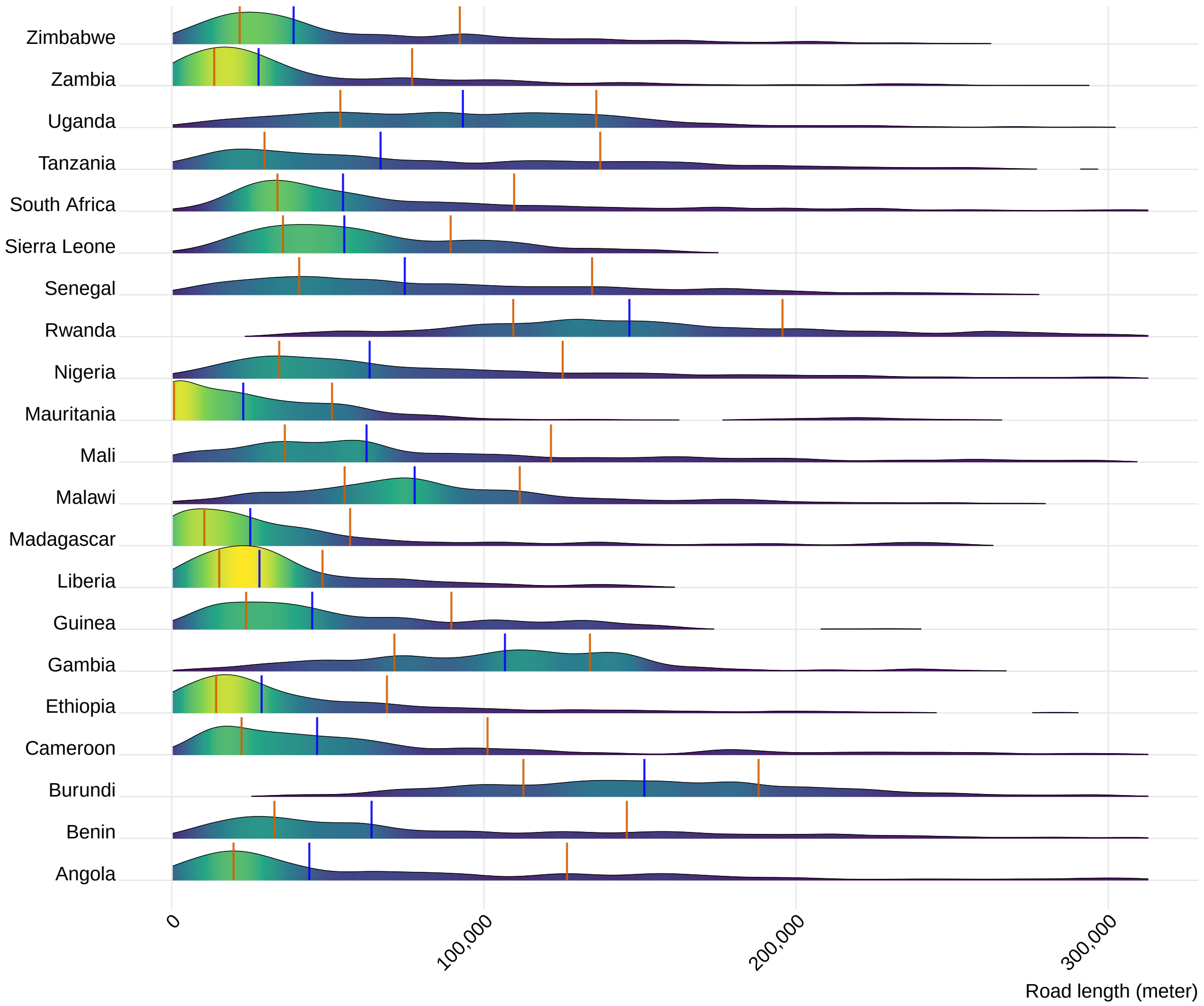

Notes: This figure shows the distribution of total major road length (in meters) within a 5 km radius of each Demographic and Health Surveys (DHS) cluster, for each country. Distributions are based on the unweighted DHS sample of live births. Country-specific quartile groupings are derived from the distribution of road length values among live births within each country. Vertical lines indicate the 25th (orange), 50th (median; blue), and 75th (orange) percentiles used to define quartiles. For visualization purposes, the top 1% of road length values are excluded. Corresponding quartile thresholds for each country are reported in Supplementary Table 20.

Supplementary Table 20: Road length quartiles

Country	Q0	Q25	Q50	Q75	Q100
Angola	0.000	19,806.320	44,076.58	126,613.24	348,632.9
Benin	0.000	32,901.525	64,018.23	145,785.63	332,616.6
Burundi	30,146.383	112,661.064	151,407.71	187,980.69	313,139.1
Cameroon	0.000	22,369.221	46,576.65	101,172.20	313,530.0
Ethiopia	0.000	14,238.947	28,800.16	68,942.24	571,430.9
Gambia	7,728.844	71,327.640	106,745.05	133,970.58	264,522.9
Guinea	0.000	23,850.897	45,006.74	89,575.39	237,476.4
Liberia	0.000	15,229.738	28,105.11	48,307.44	153,212.5
Madagascar	0.000	10,469.522	25,164.23	57,152.36	301,702.7
Malawi	0.000	55,394.378	77,812.74	111,479.72	282,053.5
Mali	0.000	36,230.562	62,407.74	121,482.46	302,053.4
Mauritania	0.000	744.629	22,920.05	51,360.39	265,726.9
Nigeria	0.000	34,437.568	63,398.88	125,234.50	429,989.8
Rwanda	33,150.923	109,403.963	146,607.20	195,646.65	422,032.1
Senegal	0.000	40,837.742	74,659.75	134,659.68	371,190.2
Sierra Leone	0.000	35,643.887	55,292.09	89,355.94	177,139.5
South Africa	0.000	33,867.046	54,871.64	109,685.33	386,239.7
Tanzania	0.000	29,725.811	66,897.80	137,258.21	464,351.2
Uganda	0.000	54,014.080	93,263.75	136,011.87	408,053.3
Zambia	0.000	13,630.418	27,818.05	76,999.06	455,252.7
Zimbabwe	0.000	21,783.860	39,049.73	92,285.60	270,654.0

Notes: This table reports the country-specific percentile values (Q0, Q25, Q50, Q75, Q100) of total major road length (in meters) within a 5 km radius of each Demographic and Health Surveys (DHS) cluster, based on the unweighted DHS sample of live births. These percentiles are used to define within-country quartile groupings, where quartile 1 corresponds to households located in areas with the lowest total major road length, and quartile 4 to those in areas with the highest.

7.3 Climate zone classification of DHS clusters

We assigned each DHS cluster to a Köppen–Geiger climate zone using the global classification of period 1991-2020 from Beck et al. (2023)[9]. To address missing values in coastal locations where cluster coordinates did not intersect with the raster, we used the 0.1° (11 km) resolution version, which is also provided by Beck et al.(2023) and derived from the original 0.01° product by majority resampling.

The original 30 Köppen–Geiger climate types were aggregated into three major zones represented in our Sub-Saharan Africa sample: Tropical, Arid, and Temperate. Cold and Polar zones were absent from our study area. Climate zones were assigned by extracting the classification value at each DHS cluster's displaced coordinates.

Supplementary Fig. 16 shows the distribution of DHS clusters across the three climate zones. This classification is used in our climate-zone heterogeneity analysis (Supplementary Information Section 5.5, Supplementary Table 18).

Supplementary Fig. 16: Climate zone classification of DHS clusters

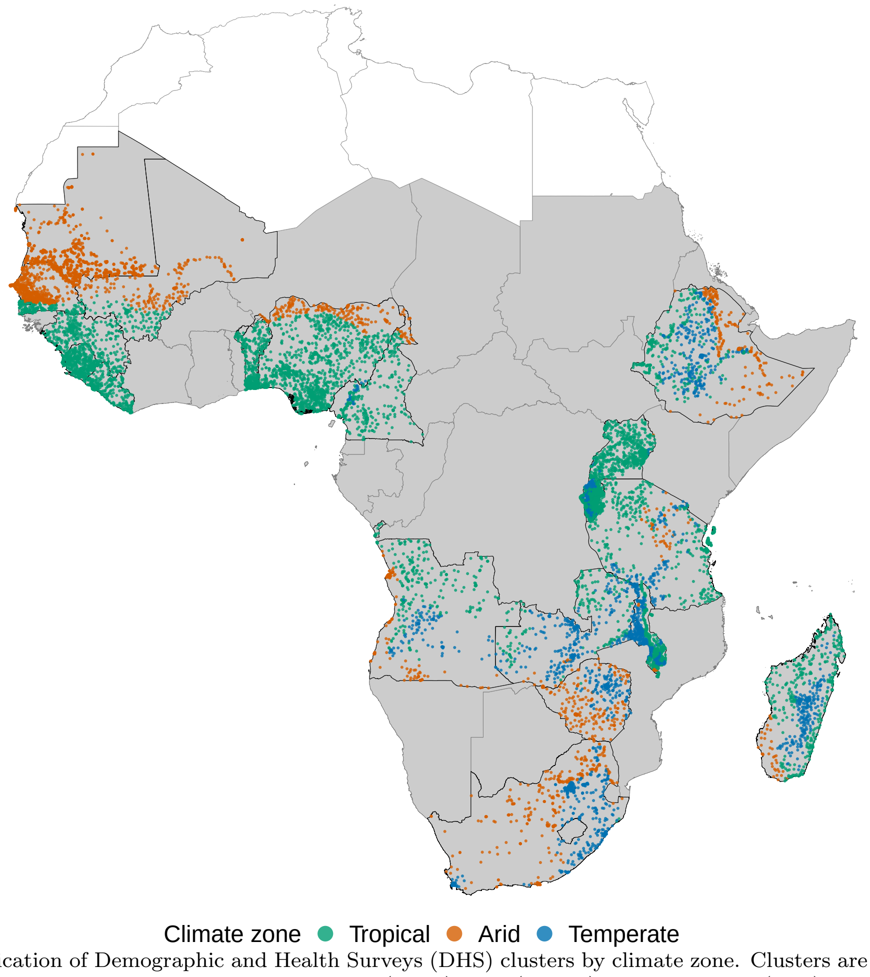

Notes: Classification of Demographic and Health Surveys (DHS) clusters by climate zone. Clusters are assigned to one of three Köppen–Geiger climate zones—tropical (green), arid (orange), and temperate (blue)—using the 0.1° (∼11 km) resolution dataset from Beck et al. (2023). This classification is used in our climate-zone heterogeneity analysis (Supplementary Information Section 5.5, Supplementary Table 18). Administrative boundaries were derived from the Database of Global Administrative Areas (GADM)[1].

References

1. Global Administrative Areas. *GADM Database of Global Administrative Areas version 4.1.0* https://gadm.org.
2. *Guidelines on the definition and characterization of extreme weather and climate events* in collab. with World Meteorological Organization (WMO). ISBN: 9789263113108 Num Pages: 27. Geneva, 2023.
3. Fernández-Val, I. & Weidner, M. Individual and time effects in nonlinear panel models with large N, T. *Journal of Econometrics* **192,** 291–312 (2016).
4. Neyman, J. & Scott, E. L. Consistent Estimates Based on Partially Consistent Observations. *Econometrica* **16,** 1–32 (1948).
5. James, W. *et al.* Gridded birth and pregnancy datasets for Africa, Latin America and the Caribbean. *Scientific Data* **5,** 180090 (2018).
6. Hierink, F. *et al.* Differences between gridded population data impact measures of geographic access to healthcare in sub-Saharan Africa. *Communications Medicine* **2,** 117 (2022).
7. Maina, J. *et al.* A spatial database of health facilities managed by the public health sector in sub Saharan Africa. *Scientific Data* **6,** 134 (2019).
8. Bondarenko, M., Kerr, D., Sorichetta, A. & Tatem, A. *Census/projection-disaggregated gridded population datasets for 189 countries in 2020 using Built-Settlement Growth Model (BSGM) outputs* in collab. with Bondarenko, M., Kerr, D., Sorichetta, A. & Tatem, A. 2020.
9. Beck, H. E. *et al.* High-resolution (1 km) Köppen-Geiger maps for 1901–2099 based on constrained CMIP6 projections. *Scientific Data* **10,** 724 (2023).
