# Supplementary material for: Impact of extreme precipitation events on facility-based births in 21 sub-Saharan African countries
Source: Nat Commun. 2026 May 4;17:6196. doi: 10.1038/s41467-026-72547-w (PMC13369172; doi:10.1038/s41467-026-72547-w)
Supplement: Supplementary file 2 — Reporting Summary [file 41467_2026_72547_MOESM2_ESM.pdf]

nature portfolio

Corresponding author(s): Oumar Aly Ba

Last updated by author(s): 13 January 2025

Reporting Summary

Nature Portfolio wishes to improve the reproducibility of the work that we publish. This form provides structure for consistency and transparency in reporting. For further information on Nature Portfolio policies, see our Editorial Policies and the Editorial Policy Checklist.

Statistics

For all statistical analyses, confirm that the following items are present in the figure legend, table legend, main text, or Methods section.

n/a	Confirmed	
☐	☒	The exact sample size (n) for each experimental group/condition, given as a discrete number and unit of measurement
☐	☒	A statement on whether measurements were taken from distinct samples or whether the same sample was measured repeatedly
☐	☒	The statistical test(s) used AND whether they are one- or two-sided<br>*Only common tests should be described solely by name; describe more complex techniques in the Methods section.*
☐	☒	A description of all covariates tested
☐	☒	A description of any assumptions or corrections, such as tests of normality and adjustment for multiple comparisons
☐	☒	A full description of the statistical parameters including central tendency (e.g. means) or other basic estimates (e.g. regression coefficient) AND variation (e.g. standard deviation) or associated estimates of uncertainty (e.g. confidence intervals)
☐	☒	For null hypothesis testing, the test statistic (e.g. F, t, r) with confidence intervals, effect sizes, degrees of freedom and P value noted<br>*Give P values as exact values whenever suitable.*
☒	☐	For Bayesian analysis, information on the choice of priors and Markov chain Monte Carlo settings
☒	☐	For hierarchical and complex designs, identification of the appropriate level for tests and full reporting of outcomes
☐	☒	Estimates of effect sizes (e.g. Cohen's d, Pearson's r), indicating how they were calculated

Our web collection on statistics for biologists contains articles on many of the points above.

Software and code

Policy information about availability of computer code

Data collection	We utilize publicly available household surveys from the Demographic and Health Surveys and publicly available spatial and satellite data (CHIRPS for daily rainfall and OpenStreetMap for road network). Travel time data were obtained from a previous publication cited in the paper. These data were processed in R.
Data analysis	All statistical analyses were conducted in R (version 4.2.2). A full list of R packages and their versions is provided in the README file accompanying the code repository. The code used for the statistical analyses and for generating the figures and tables is publicly available in the following GitHub repository: https://github.com/oumaralyba/EPEs_and_FBs and archived in Zenodo at https://doi.org/10.5281/zenodo.19257584.

For manuscripts utilizing custom algorithms or software that are central to the research but not yet described in published literature, software must be made available to editors and reviewers. We strongly encourage code deposition in a community repository (e.g. GitHub). See the Nature Portfolio guidelines for submitting code & software for further information.

Data

Policy information about availability of data

All manuscripts must include a data availability statement. This statement should provide the following information, where applicable:

- Accession codes, unique identifiers, or web links for publicly available datasets
- A description of any restrictions on data availability
- For clinical datasets or third party data, please ensure that the statement adheres to our policy

We are not permitted to redistribute the household survey data from the Demographic and Health Surveys (DHS) Program. These data can be accessed directly from the DHS website upon registration and approval. The travel-time data used in this study were obtained from Fleur Hierink, corresponding author of the previous publication from which these data originate, and are therefore not controlled by us for public redistribution; requests for access should be directed to the original data provider. CHIRPS precipitation data, OpenStreetMap road network data, WorldPop population data and administrative boundaries data (GADM) are publicly available from their respective websites. All replication code used for data analysis and figure generation is publicly available in a GitHub repository, together with instructions for accessing the input data.

Research involving human participants, their data, or biological material

Policy information about studies with human participants or human data. See also policy information about sex, gender (identity/presentation), and sexual orientation and race, ethnicity and racism.

Reporting on sex and gender	The study did not differentiate or analyze data based on sex or gender. Our primary focus was on assessing the impact of Extreme Precipitation Events (EPEs) on facility-based births, without making distinctions based on the sex or gender of the live births. As such, no sex- or gender-based analyses were performed. Although the DHS data predominantly include information on female respondents due to the nature of surveys, data on male respondents is also collected but was not relevant to the outcome measures of this study.
Reporting on race, ethnicity, or other socially relevant groupings	The study does not use any socially relevant groupings as proxies for any socioeconomic or other socially constructed variables.
Population characteristics	See above.
Recruitment	Participants for the DHS data were recruited through a stratified sampling design that ensures representation across different geographic and socioeconomic strata within each country. This sampling method aims to ensure that the surveys are nationally representative of the demographic and health characteristics of the population. Further details on the sampling process are elaborated in the next sections.
Ethics oversight	- Institute of Global Health & Environmental Sciences, University of Geneva<br>- The study was conducted using secondary data from the DHS program, which are available on request, and thus no ethical approval was needed. The DHS received government permission and followed ethical practices including informed consent and assurance of confidentiality. Details of the ethical review process of DHS are available on the program's website https://dhsprogram.com/Methodology/Protecting-the-Privacy-of-DHS-Survey-Respondents.cfm

Note that full information on the approval of the study protocol must also be provided in the manuscript.

Field-specific reporting

Please select the one below that is the best fit for your research. If you are not sure, read the appropriate sections before making your selection.

☐ Life sciences ☒ Behavioural & social sciences ☐ Ecological, evolutionary & environmental sciences

For a reference copy of the document with all sections, see nature.com/documents/nr-reporting-summary-flat.pdf

Behavioural & social sciences study design

All studies must disclose on these points even when the disclosure is negative.

Study description	Data are quantitative observational, derived from household surveys conducted across 21 sub-Saharan African countries. The study investigated the impact of Extreme Precipitation Events (EPEs) on facility-based births.
Research sample	The research sample consists of live births to mothers aged 15–49 across 21 sub-Saharan African countries, as measured by the Demographic and Health Surveys (DHS). The sample is representative of the populations of these countries.
Sampling strategy	DHS employs stratified random sampling by first selecting enumeration areas proportional to population size, then randomly sampling 10-20 mothers of childbearing age within each area. This sampling method ensures national representation in each country. Please refer to: https://dhsprogram.com/What-We-Do/Survey-Types/DHS-Methodology.cfm#CP_JUMP_16156
Data collection	Data were collected through household surveys administered by the Demographic and Health Surveys (DHS). The surveys followed the sampling protocol previously described and were conducted in respondents' households. DHS-trained enumerators performed structured interviews to measure a range of health behaviors and outcomes. This included recording the date and setting of all live

births reported by the interviewed mothers in the last five years prior to the survey date.

For more detailed information on survey methodologies, please visit the DHS website: https://dhsprogram.com/What-We-Do/Survey-Types/DHS.cfm

Timing

The DHS data we use were collected between 2015 and 2021. The exact data collection period varied across countries as per their national DHS schedules.

Data exclusions

We use all geo-referenced sub-Saharan African surveys conducted by the DHS from phase 7 onward, which incorporate a crucial variable for our analysis—namely, the day, month, and year of birth. Only surveys that were available at the time of data extraction and initial analysis in May 2023 were used.

We exclude 4,582 live births with missing CHIRPS precipitation records, primarily from coastal regions where CHIRPS spatial data did not intersect with landmasses where DHS data were geo-referenced. These exclusions were not based on pre-established criteria. We excluded these 4,582 live births as precipitation can vary greatly over short distances and time windows and thereby introduces inaccuracies. The robustness of the results was tested by including these records by matching them to the nearest location with available CHIRPS precipitation datasets as described in the paper.

Non-participation

The response rates of the DHS Surveys can be found on: https://dhsprogram.com/What-We-Do/Survey-Types/DHS-Methodology.cfm

Randomization

As described in the paper, the timing of birth dates and exposure to Extreme Precipitation Events (EPEs) are considered quasi-random, conditional on our comprehensive set of fixed effects. This quasi-randomness arises because both the timing of birth and EPE occurrence within short time windows are influenced by factors beyond the control of the DHS respondents.

Our analysis controlled for various maternal and household characteristics such as the mother's age, education, and household wealth. We also accounted for time-invariant factors at the DHS cluster level using DHS cluster fixed effects and addressed unobserved country-level shocks and seasonal variations using country-day-of-birth fixed effects.

Reporting for specific materials, systems and methods

We require information from authors about some types of materials, experimental systems and methods used in many studies. Here, indicate whether each material, system or method listed is relevant to your study. If you are not sure if a list item applies to your research, read the appropriate section before selecting a response.

Materials & experimental systems

n/a	Involved in the study
☒	☐ Antibodies
☒	☐ Eukaryotic cell lines
☒	☐ Palaeontology and archaeology
☒	☐ Animals and other organisms
☒	☐ Clinical data
☒	☐ Dual use research of concern
☒	☐ Plants

Methods

n/a	Involved in the study
☒	☐ ChIP-seq
☒	☐ Flow cytometry
☒	☐ MRI-based neuroimaging

Plants

Seed stocks

Report on the source of all seed stocks or other plant material used. If applicable, state the seed stock centre and catalogue number. If plant specimens were collected from the field, describe the collection location, date and sampling procedures.

Novel plant genotypes

Describe the methods by which all novel plant genotypes were produced. This includes those generated by transgenic approaches, gene editing, chemical/radiation-based mutagenesis and hybridization. For transgenic lines, describe the transformation method, the number of independent lines analyzed and the generation upon which experiments were performed. For gene-edited lines, describe the editor used, the endogenous sequence targeted for editing, the targeting guide RNA sequence (if applicable) and how the editor was applied.

Authentication

Describe any authentication procedures for each seed stock used or novel genotype generated. Describe any experiments used to assess the effect of a mutation and, where applicable, how potential secondary effects (e.g. second site T-DNA insertions, mosiacism, off-target gene editing) were examined.
