# Supplementary material for: Impact of extreme precipitation events on facility-based births in 21 sub-Saharan African countries
Source: Nat Commun. 2026 May 4;17:6196. doi: 10.1038/s41467-026-72547-w (PMC13369172; doi:10.1038/s41467-026-72547-w)
Supplement: Supplementary file 3 — Transparent Peer Review File [file 41467_2026_72547_MOESM3_ESM.pdf]

nature portfolio

Peer Review File

Impact of extreme precipitation events on facility-based births in 21 sub-Saharan African countries

Corresponding Author: Mr Oumar Aly Ba

This manuscript has been previously reviewed at another journal. This document only contains information relating to versions considered at Nature Communications.

This file contains all reviewer reports in order by version, followed by all author rebuttals in order by version.

Version 0:

Reviewer comments:

Reviewer #1

(Remarks to the Author)
I reviewed this paper back in February 2025 for [redacted]. The authors have taken into consideration my comments from that first review and have revised the paper accordingly. I have no additional comments.

(Remarks on code availability)
The codes seem to provide sufficient details for reproducing the analysis.

Reviewer #2

(Remarks to the Author)
The authors have done an excellent job addressing my questions as well as those raised by other reviewers. I have no further specific questions, only a few minor comments:

•The authors provided a detailed explanation regarding the quality of the exposure assessment. I agree that using a 5×5 km resolution is appropriate, especially considering the geolocation of live births. However, I would caution against describing it as “high quality” in the abstract. Rainfall can be highly localized.
•The authors have elaborated on the rationale behind Figure 1. While I appreciate the effort, I remain unconvinced due to the density of information presented. Some lag periods could potentially be removed to improve clarity. Additionally, the current color scheme may be difficult for color-blind readers to interpret. I suggest using different shapes (e.g., circles, triangles, squares) to distinguish between lines and improve accessibility.

(Remarks on code availability)

Reviewer #3

(Remarks to the Author)
The authors present an interesting study on the effect of extreme precipitation events on access to birth facilities in sub-Saharan Africa. The topic is timely and relevant and the research design is well thought out. The authors report multiple sensitivity analyses demonstrating that the research question has been considered from multiple angles. Overall it’s a well-designed and written study, although I have some comments.
The primary concern is that the type of birth is not adequately considered. The main mention of this seems to be in methods, “This time window accounts for ... scheduled inductions and caesarean sections..” It’s reasonable to assume some difference between natural birth and scheduled procedures, which may lead to more planning ahead (including for extreme weather). If this data is available, it should be accounted for in the analysis, otherwise elaborated on in the discussion.
The second key concern is the choice and reporting of the statistical significance thresholds (e.g. “marginally significant at p=0.0585 and p=0.0978”). P-values > 0.05 are conventionally not statistically significant (the “marginally significant”

expression is more often used for a value like 0.049, or p less than 0.05 but not less than a more stringent adjusted threshold). Similarly, the asterisk for 0.1 in the various tables can be misleading and should be removed.
Minor comments:
-In results, under sensitivity analysis, describe more clearly what is meant by ‘most recent live birth’.
-In the same section, remove ‘at conventional thresholds’ (in “..statistically significant at conventional thresholds”).
-In Heterogeneity of EPE impacts, “each other’s” should be “other”.
-The use of (1), (2), (3).. in tables is somewhat confusing, clarify what the numbers refer to (presumably the different models).
-In Figure 1, it’s difficult to see the different colors and lines, consider limiting the x-axis to the chosen threshold +/-5% for better visualisation and moving the full figure to supplementary.
-For Figure 2 and the related results section, clarify the use of a leave-one-out type of approach with multiple models vs interaction terms within the one multivariable model.
-In Figure 3, “1’000” should be “1,000” or “1000.”
-Page numbers can be removed from supplementary references, table or figure number is informative enough.
-The authors may wish to consider ordering supplementary tables in the order they appear in the main text to make it easier to follow through.

(Remarks on code availability)

Open Access This Peer Review File is licensed under a Creative Commons Attribution 4.0 International License, which permits use, sharing, adaptation, distribution and reproduction in any medium or format, as long as you give appropriate credit to the original author(s) and the source, provide a link to the Creative Commons license, and indicate if changes were made.
In cases where reviewers are anonymous, credit should be given to 'Anonymous Referee' and the source.
The images or other third party material in this Peer Review File are included in the article’s Creative Commons license, unless indicated otherwise in a credit line to the material. If material is not included in the article’s Creative Commons license and your intended use is not permitted by statutory regulation or exceeds the permitted use, you will need to obtain permission directly from the copyright holder.
To view a copy of this license, visit https://creativecommons.org/licenses/by/4.0/

Author Responses to Initial Comments:

Reviewer comments in black
Our replies in green
New text in the manuscript in red

Additional note (not prompted by reviewer comments): During final checks, we identified a coding oversight affecting the computation of the confidence interval for the 2015 attribution analysis: the zonal-statistics step was inadvertently computed over the full sub-Saharan Africa birth raster rather than being restricted to the 21 study countries. After correcting this, the estimated number of additional non-facility births attributable to EPEs in 2015 is 29,084 (95% CI 8,660 to 49,508), replacing the previous estimate of 29,165 (95% CI 13,405 to 76,634). We have updated these numbers in the manuscript. This correction does not affect any regression estimates or the qualitative conclusions.

REVIEWER COMMENTS

Reviewer #1 (Remarks to the Author):

I reviewed this paper back in February 2025 for [redacted]. The authors have taken into consideration my comments from that first review and have revised the paper accordingly. I have no additional comments.

We thank the reviewer for their positive assessment and are pleased that our revisions have addressed their concerns.

Reviewer #1 (Remarks on code availability):

The codes seem to provide sufficient details for reproducing the analysis.

We thank the reviewer for their evaluation of the code.

Reviewer #2 (Remarks to the Author):

The authors have done an excellent job addressing my questions as well as those raised by other reviewers. I have no further specific questions, only a few minor comments:

•The authors provided a detailed explanation regarding the quality of the exposure assessment. I agree that using a 5×5 km resolution is appropriate, especially considering the geolocation of live births. However, I would caution against describing it as "high quality" in the abstract. Rainfall can be highly localized.

We thank the reviewer for this positive comment. We have removed the term "high-resolution precipitation data" from the abstract and now describe the exposure data more neutrally as:

> **Abstract – Lines 18-42:**
> "**Abstract**
> Climate change impacts on health outcomes are increasingly recognized, yet the effects of Extreme Precipitation Events (EPEs) on geographical access to and timely use of health facilities for childbirth remain underexplored. We investigated how EPEs influence giving birth in a facility in 21 sub-Saharan African (SSA) countries by combining Demographic and Health Survey data (2015-2021) with gridded daily precipitation data at 5×5 km resolution. Using a linear probability model with a three-day exposure window before each date of birth, we analyzed 256,101 live births from 12,948 locations, defining EPEs as daily rainfall exceeding the 85th percentile of local historical distribution. We found that each additional day of EPE exposure in the three-day window significantly reduced facility-based births by -10.753 per 1,000 live births (95% CI -18.304 to -3.202), with exposure ranging from 0 to 3 days, representing a 1.886% decrease from

baseline. This reduction remained consistent across varying EPE definitions (50th percentile – 95th percentile) and was less pronounced with longer exposure windows, suggesting events closer to birth date have greater impact. Sustained moderate to heavy rainfall over multiple days also lowers facility use, indicating that barriers can arise both suddenly and cumulatively. The effect is likely more pronounced among middle-income households (-17.212 per 1,000 live births, 95% CI -32.510 to -1.913), car-owning households (-29.492 per 1,000 live births, 95% CI -50.195 to -8.787) and in locations with greater geographical barriers. We estimate approximately 29,084 additional non-facility births (95% CI 8,660 to 49,508) were attributable to EPEs in 2015. Our findings provide evidence that EPE exposure increases non-facility births that lack the safety net of skilled attendance and emergency obstetric resources. This emphasizes the critical need to incorporate climate resilience into healthcare and road infrastructure planning across SSA to ensure continuous access to facility-based care during extreme weather events and safeguard maternal and newborn health."

•The authors have elaborated on the rationale behind Figure 1. While I appreciate the effort, I remain unconvinced due to the density of information presented. Some lag periods could potentially be removed to improve clarity. Additionally, the current color scheme may be difficult for color-blind readers to interpret. I suggest using different shapes (e.g., circles, triangles, squares) to distinguish between lines and improve accessibility.

We thank the reviewer for this helpful suggestion. We revised Figures 1 and 2 to improve clarity and accessibility. Figure 1 is now limited to pre-birth exposure windows from 3 to 7 days, while the full set of results has been moved to a new Supplementary Figure 5. Further details are provided in our response to Reviewer #3.

We also updated colors and visibility across Supplementary Figures to improve accessibility (Supplementary Figs. 1, 2, 3, 4, 5, 7, 8, 9, 10, 13, 14, 15, and 16).

Reviewer #3 (Remarks to the Author):

The authors present an interesting study on the effect of extreme precipitation events on access to birth facilities in sub-Saharan Africa. The topic is timely and relevant and the research design is well thought out. The authors report multiple sensitivity analyses demonstrating that the research question has been considered from multiple angles. Overall it's a well-designed and written study, although I have some comments.

The primary concern is that the type of birth is not adequately considered. The main mention of this seems to be in methods, "This time window accounts for ... scheduled inductions and caesarean sections.." It's reasonable to assume some difference between natural birth and scheduled procedures, which may lead to more planning ahead (including for extreme weather). If this data is available, it should be accounted for in the analysis, otherwise elaborated on in the discussion.

We thank the reviewer for this insightful comment. We agree that, in principle, scheduled deliveries (e.g., elective caesarean sections or inductions) could allow greater scope for anticipatory behaviors—such as traveling earlier or rescheduling—relative to "natural" deliveries.

However, we face a data limitation: the DHS does not provide a harmonized variable across countries and survey rounds that distinguishes **scheduled/elective** versus **"natural"** deliveries. While DHS consistently records whether a birth was by caesarean section, additional information on the *timing of the caesarean decision* (e.g., before vs after labor started) is only collected for caesarean births and is not available for non-caesarean births by construction. We therefore cannot consistently account for "scheduled" versus "natural" delivery type in our analysis.

To address the reviewer's concern regarding "planning ahead," we point to our distributed lag linear model (DLM), which estimates effects in nine non-overlapping 3-day intervals spanning 14 days before to 12 days after birth. We find that only the interval ending on the day of birth ([t−2, t]) is statistically significant, with no statistically significant effects in earlier or later intervals (Supplementary Fig. 6). This

pattern is not consistent with systematic anticipatory adjustments (e.g., routinely shifting delivery location several days in advance) driving the main result.

In response to the reviewer's suggestion, we revised the Methods description of the 3-day exposure window to focus on the most defensible rationale—alignment between the recorded day of birth and the peri-partum period where labor and travel likely occur—and to avoid implying that we directly model postponement of scheduled procedures.

> **Method: Extreme precipitation events – Lines 931-943:**
> "We spatially link the DHS data with the CHIRPS precipitation data by using the coordinates of the DHS clusters. We consider a three-day exposure window, spanning from two days before birth to the day of birth (inclusive), since various factors can shift the recorded day of birth and the subsequent time window in which pregnant women are presumably "*en route*" to a health facility. This time window accounts for births that happen shortly after midnight, prolonged labor durations shifting the day of birth, ~~the potential postponement of scheduled inductions and caesarean sections due to overwhelmed healthcare systems~~, and longer travel times to health facilities. Additionally, EPEs can significantly alter local infrastructure and transportation costs, affecting travel conditions for extended days. Thus, the three-day exposure window allows us to capture local time-varying travel conditions caused by EPEs while pinpointing the period during which women likely began labor and initiated travel to access obstetric care."

The second key concern is the choice and reporting of the statistical significance thresholds (e.g. "marginally significant at p=0.0585 and p=0.0978"). P-values > 0.05 are conventionally not statistically significant (the "marginally significant" expression is more often used for a value like 0.049, or p less than 0.05 but not less than a more stringent adjusted threshold). Similarly, the asterisk for 0.1 in the various tables can be misleading and should be removed.

We thank the reviewer for this comment. We have removed "marginally significant" and similar phrasing throughout the manuscript and Supplementary Information, and we now describe them as not statistically significant, focusing on effect sizes.

We revised the following parts of the manuscript and Supplementary Information accordingly:

> **Results: Heterogeneity of EPE impacts– Lines 486-552:**
> "Women living in clusters with longer travel times to health facilities had more pronounced effects of EPEs, although the interactions do not significantly differ from each other (quartile 1 versus quartile 3: p=0.0585; quartile 2 versus quartile 3: p=0.0978; Supplementary Table 15) ~~(difference between quartile 1 versus quartile 3, and quartile 2 versus quartile 3 are only marginally significant at p=0.0585 and p= 0.0978 respectively)~~. Women in areas within the third travel time quartile (e.g. the third group with the longest national travel times) had a significant decrease of -20.788 facility-based births per 1,000 live births (95% CI -32.929 to -8.645). However, when considering the median travel time for interaction instead of quartiles, interaction terms were significantly different ~~at conventional level~~ with greater effect for women in areas above the national median travel time (p=0.0465, Supplementary Table 15)."

> **Results: Heterogeneity of EPE impacts– Lines 555-560:**
> "Car-owning households displayed a more pronounced response to EPEs, with a decrease of -29.492 per 1,000 live births (95% CI -50.195 to -8.787, -5.173% decrease from the sample mean) ~~, a difference that is marginally significant compared with non-car-owning households (p = 0.0638). This effect entirely offset the benefits associated with car ownership (18.038 per 1000 live births, 95% CI 5.937 to 30.138).~~ Although the estimated difference in EPE response relative to non-car-owning households was not statistically significant (p=0.0638), the point estimates suggest that EPE-related disruptions may offset the baseline advantage associated with car ownership (18.038 per 1,000 live births, 95% CI 5.937 to 30.138)."

> **Results: Non-facility births attributed to EPEs in 2015– Lines 577-586:**

"Finally, we explored the stability of the attribution of additional non-facility births using country-specific coefficients (Supplementary Fig. 12-13). Liberia, Nigeria, and Mali showed the greatest increase in the likelihood of non-facility births with country-specific coefficients implying a change of 4.1%, 6.2% and 3.1% relative to their national average facility-based births. In contrast, Madagascar— the only country frequently affected by cyclones—had a negative number of additional non-facility births with a coefficient marginally significant (27.082 per 1000 live births, 95% CI -1.635 to 55.799, p=0.0645). This is indicative of greater mitigating factors or a distinct change in health-seeking behaviors prompted by cyclone's heavy rainfall. In contrast, Madagascar—the only country in our study frequently exposed to cyclones—shows a positive country-specific coefficient (27.082 per 1,000 live births, 95% CI –1.635 to 55.799, p=0.0645), which implies an increase in facility-based births attributable to EPEs in the attribution exercise, although the estimate is imprecise and not statistically distinguishable from zero. This pattern may reflect context-specific preparedness or behavioral responses associated with cyclone-related rainfall."

Discussion – Lines 686-708:
"Our results suggest potential vulnerability among car-owning households: their health-seeking behaviors deviated more from baseline than those of non-car-owning households, although the difference between groups was not statistically significant (p = 0.064) and should be interpreted cautiously. While imprecise, this pattern is consistent with the possibility that car ownership does not necessarily ensure greater mobility during EPEs, because geographic access depends on day-to-day road passability and a well-functioning road network. Under EPEs, car-owning households may experience increased mobility costs—in time, risk, or expense—that erode their usual mobility advantage. This vulnerability may be specific to EPEs: during more modest or seasonal rainfall, car-owning households may retain their baseline advantage likely retain their baseline advantage, while increased mobility constraints remain concentrated among households without motorized transport. Future research could further explore how moderate rainfall events interact with transport ownership and road conditions, particularly in settings with fragile infrastructure."

Supplementary Information Section 5.5:
"The difference between tropical and arid zones was not statistically significant (p=0.098), and the tropical--temperate difference was similarly not significant (p=0.154)."

Regarding the (p<0.1) asterisk, we acknowledge the reviewer's concern. This notation is commonly used, and the table footnotes state the exact meaning of each symbol, which we believe limits any risk of misinterpretation. To align with the reviewer's point, we have ensured that the manuscript text does not describe 0.1>p>0.05 estimates as marginally significant, while retaining the table notation as defined. If preferred by the editorial team, we can remove the p<0.1 symbol and rely solely on 95% confidence intervals and exact p-values.

Minor comments:
-In results, under sensitivity analysis, describe more clearly what is meant by 'most recent live birth'.

We thank the reviewer for highlighting. In this sensitivity analysis, "most recent live birth" refers to the most recent live birth reported by each woman in the DHS birth history within the five years preceding the survey; all earlier live births to the same woman during this period were excluded. For example, if a woman reported two live births in the five years before the survey, occurring in 2017 and 2019, only the 2019 birth was retained in the analysis.

To clarify this in the manuscript, we have revised the relevant sentence in the Results section as follows:

Results: Sensitivity analysis – Lines 163-164:
"Fifth, we restricted our sample to the most recent live birth reported by each woman in the 5 years preceding the survey."

-In the same section, remove 'at conventional thresholds' (in "..statistically significant at conventional thresholds").

We thank the reviewer for this comment. We revised the three following parts of the paper accordingly:

Results: Sensitivity analysis – Lines 443-446:
"Finally, our results remained qualitatively similar across various fixed effect specifications, although some estimates were less precise and not statistically distinguishable from zero ~~were not statistically significant at conventional thresholds~~ (Supplementary Table 11)."

Results: Heterogeneity of EPE impacts– Lines 486-552:
"Women living in clusters with longer travel times to health facilities had more pronounced effects of EPEs, although the interactions do not significantly differ from each other (quartile 1 versus quartile 3: p=0.0585; quartile 2 versus quartile 3: p=0.0978; Supplementary Table 15) ~~(difference between quartile 1 versus quartile 3, and quartile 2 versus quartile 3 are only marginally significant at p=0.0585 and p= 0.0978 respectively)~~. Women in areas within the third travel time quartile (e.g. the third group with the longest national travel times) had a significant decrease of -20.788 facility-based births per 1,000 live births (95% CI -32.929 to -8.645). However, when considering the median travel time for interaction instead of quartiles, interaction terms were significantly different ~~at conventional level~~ with greater effect for women in areas above the national median travel time (p=0.0465, Supplementary Table 15)."

Discussion – Lines 725-735:
"Adaptation and preparedness strategies can influence the incentive for opting for facility-based births. Local time-varying factors, such as anticipatory behaviors prompted by local news or governmental interventions during EPEs, could modify expected health seeking behavior. Notably, Madagascar presents a unique case in our country-specific analysis: the coefficient is positive (27.082 per 1,000 live births, 95% CI –1.635 to 55.799, p=0.0645; Supplementary Fig. 12). While this estimate is not statistically significant and should be interpreted cautiously, it could reflect cyclone-related EPEs prompting different modifications in health-seeking behaviors. As the only country in our study frequently exposed to cyclones, Madagascar may have developed effective warning strategies[42]. ~~Notably, Madagascar presents a unique case in our study as the only country to exhibit a positive coefficient with significance close to the conventional level (Supplementary Fig. 12). This suggests that EPEs related to cyclones may prompt different modification in health-seeking behaviors. Madagascar may have developed effective warning strategies due to its frequent exposure to cyclones[42].~~ Indeed, countries exposed to greater tropical cyclone climates display relatively smaller losses when exposed to an actual tropical cyclone event, indicating that adaptation to this climatological risk occurs[43]."

-In Heterogeneity of EPE impacts, "each other's" should be "other".

We thank the reviewer for this comment. The sentence now reads:

Results: Heterogeneity of EPE impacts– Lines 486-489:
"Women living in clusters with longer travel times to health facilities had more pronounced effects of EPEs, although the interactions do not significantly differ from each other (quartile 1 versus quartile 3: p=0.0585; quartile 2 versus quartile 3: p=0.0978; Supplementary Table 15) ~~(difference between quartile 1 versus quartile 3, and quartile 2 versus quartile 3 are only marginally significant at p=0.0585 and p= 0.0978 respectively)~~."

-The use of (1), (2), (3).. in tables is somewhat confusing, clarify what the numbers refer to (presumably the different models).

We thank the reviewer for highlighting this source of confusion. The numbers indeed refer to different model specifications. To clarify this, we have added a sentence in the footnote of Table 1 explaining the meaning of the numbered columns:

Table 1 footnotes:

The estimates represent the change in the probability of facility-based birth (per 1000 births) for live births exposed to an additional day with extreme precipitation event (EPE), compared with live births born in the same area with no or fewer extreme precipitation days. The analysis focuses on events occurring within a three-day time window from the day of birth and the two preceding days. The exposure thus ranges from 0 (no EPE) to 3 days (EPEs occurring on all three days). An extreme precipitation event is defined as a daily rainfall realization over the 85th percentile of the local rainfall distribution. Columns (1)-(4) present estimates from progressively adjusted model specifications, with column (4) corresponding to the fully adjusted main model discussed in the text. All specifications include maternal and household covariates. FE stands for fixed effects. SEs are clustered at the survey cluster level. Signif.. Codes: ***: 0.01, **: 0.05, *: 0.1

We have also revised the results section to refer explicitly to this main model/specification. The relevant passage now reads:

Results: Impact of EPEs on facility-based births– Lines 134-137:

"We found that EPEs significantly reduced the probability of giving birth in a facility (Table 1). In our main model (column (4) of Table 1), an additional day of extreme precipitation in the three-day window leading up to and including the day of birth was associated with a decrease of -10.753 facility-based births per 1,000 live births (95% CI -18.304 to -3.202)."

-In Figure 1, it's difficult to see the different colors and lines, consider limiting the x-axis to the chosen threshold +/-5% for better visualisation and moving the full figure to supplementary.

We thank the reviewer for this recommendation. We revised Fig. 1 by limiting the pre-birth exposure windows from 3 days to 7 days. We then added a new Supplementary Fig. 5 in the Supplementary Information that shows the full set of results across all pre-birth exposure windows.

We also revised Fig.1 and the new Supplementary Fig. 5 to improve their clarity and accessibility. We did similary across the Supplementary Figures (Supplementary Figs. 1, 2, 3, 4, 5, 7, 8, 9, 10, 13, 14, 15, and 16).

We revised these section accordingly:

Results- lines 142-145:

"When considering longer time window exposures, the effect consistently diminished across all definitions of EPEs, indicating that events occurring closer to the day of birth have a greater impact than those further back in time (Fig. 1 and Supplementary Fig. 5)."

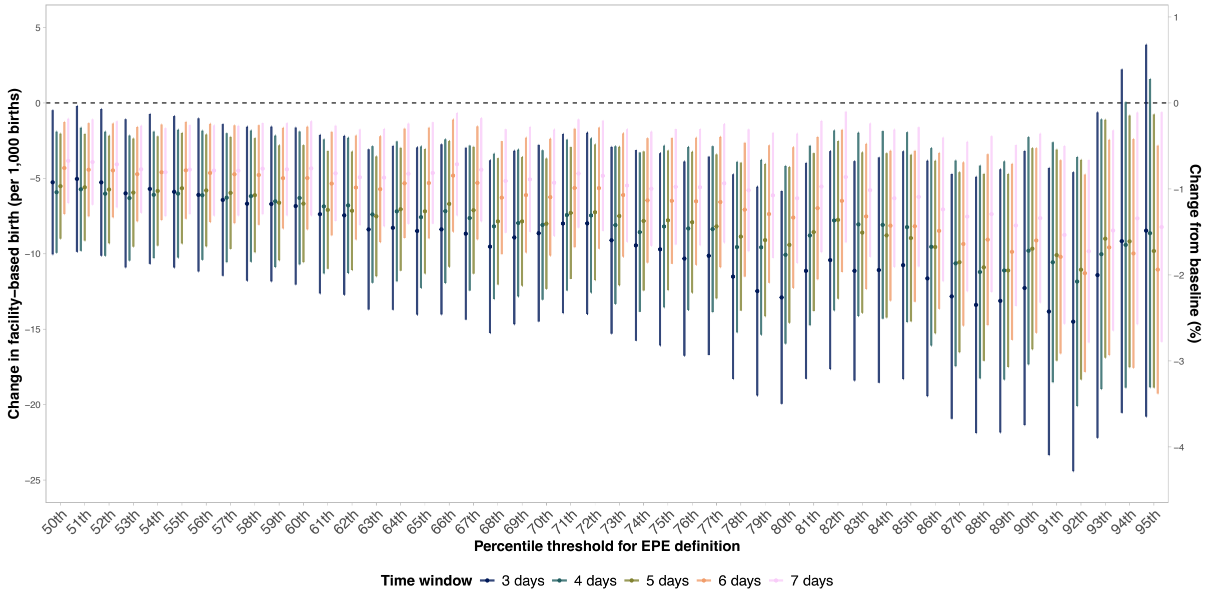

The estimates represent the change in facility-based birth rate (per 1,000 births) for an additional day with extreme precipitation. An extreme precipitation event is defined as a daily rainfall realization over a percentile threshold of the local rainfall distribution. All specifications include maternal and household covariates as well as DHS cluster and country-day-of-birth fixed effects. SEs are clustered at the survey cluster level. Error bars indicate 95% CIs. For results covering extended exposure windows up to 14 days, see Supplementary Fig. 5.

Fig. 1: Effect of EPEs on facility-based birth across percentile thresholds and time windows (3–7 days)

2 Extended time-window exposure

Supplementary Fig. 5: Effect of EPEs on facility-based birth across percentile thresholds and extended time windows (3–14 days)

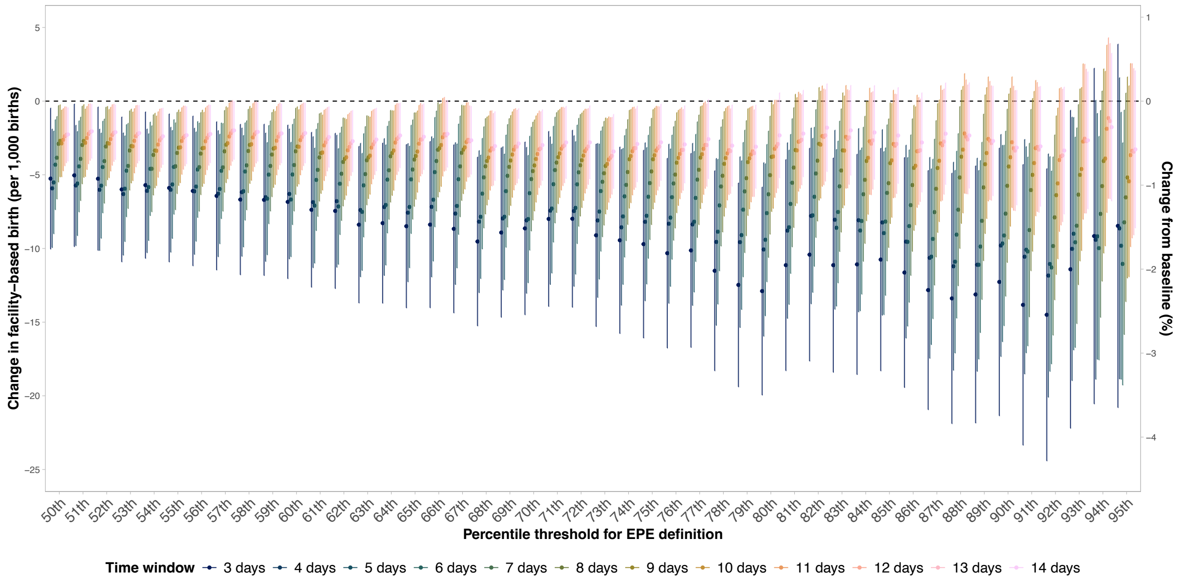

Notes: This figure extends the analysis presented in Fig. 1 of the main text by including exposure windows up to 14 days. The estimates represent the change in facility-based birth rate (per 1,000 births) for an additional day with extreme precipitation. An extreme precipitation event is defined as a daily rainfall realization over a percentile threshold of the local rainfall distribution. All specifications include maternal and household covariates as well as DHS cluster and country-day-of-birth fixed effects. SEs are clustered at the survey cluster level. Error bars indicate 95% CIs.

-For Figure 2 and the related results section, clarify the use of a leave-one-out type of approach with multiple models vs interaction terms within the one multivariable model.

We thank the reviewer for this comment. The heterogeneity analyses underlying Fig. 2 are based on interaction terms, not a leave-one-out approach. For each potential effect modifier (household wealth quintile, travel time to the nearest facility, perceived distance barrier, car ownership, and road length), we estimate a separate multivariable interaction model on the full sample that includes: (i) indicators for all categories of the modifier, and (ii) EPE exposure interacted with these category indicators, along with the full set of maternal and household covariates, DHS-cluster fixed effects, and country-day-of-birth fixed effects. We estimate separate models by modifier (rather than a single model including all modifiers and interactions) to maintain interpretability and avoid an overly parameterized specification.

The subgroup-specific point estimates in Fig. 2 are therefore category-specific marginal effects of an additional EPE day derived from these interaction models (i.e., they are not obtained by re-estimating the model separately within each subgroup, nor by omitting categories in a leave-one-out fashion).

We have clarified this in the footnotes of Fig. 2:

> **Fig. 2 footnotes:**
> "The estimates represent the change in the probability of facility-based birth (per 1,000 births) for live births exposed to an additional day with extreme precipitation event (EPE). The labels represent the change from the average prevalence of facility-based birth in the entire sample. The analysis focuses on events occurring within a three-day time window from the day of birth and the two preceding days. The exposure thus ranges from 0 (no EPE) to 3 days (EPEs occurring on all three days). An extreme precipitation event is defined as a daily rainfall realization over the 85th percentile of the local rainfall distribution. Each block of estimates (wealth, travel time, perceived access, car ownership, road length) is obtained from a separate multivariable model for the full sample, in which the EPE term is interacted with the corresponding group

variable. All specifications include maternal and household covariates as well as DHS cluster and Country-day-of-birth fixed effects. SEs are clustered at the survey cluster level. Error bars indicate 95% CIs. The corresponding regression tables are presented in Supplementary Table 9-12."

We also revised the heterogeneity section of the methods section accordingly and enhanced the flow of the writing :

Methods: Heterogeneity of EPE impacts- Lines 1074 - 1095
"We ~~also~~ test for heterogeneity in the estimated average treatment effect to assess whether certain groups or places might be more vulnerable to EPEs. We do so by interacting the EPE exposure measure with relevant covariates. We examine effect heterogeneity by household wealth quintile, travel time to the nearest health facility and perceived access. Perceived access information is provided by the DHS survey and informs on the reason why the mother has serious problems in accessing health care. Travel time—used as a proxy for geographical accessibility to health services—was modeled by Hierink et al. [66] using data on the locations of public and private not-for-profit health facilities collected between 2012 and 2018. To minimize attenuation bias resulting from the random displacement of DHS cluster's coordinates, we calculated the population-weighted median travel time within each DHS buffer, spanning 2 km and 5 km for urban and rural clusters (Supplementary Information Section 7.1). We also investigated the moderating role of household car ownership and the total length of major roads within a consistent 5 kilometers buffer around each DHS cluster (Supplementary Information Section 7.2). Major road length serves as a proxy for the availability of major road options, which may influence the degree to which EPEs disrupt access to health facilities. This fixed spatial window ensured comparability across cluster types and prevented mechanical inflation of road length measures in rural areas due to larger buffer sizes. Road network data were downloaded from OpenStreetMap on 31 January 2024. Finally, we ~~also~~ explored heterogeneity in effects by climate zone. DHS clusters were classified as tropical, arid, or temperate using Köppen–Geiger climate zones of period 1991-2020 (Supplementary Information Section 7.3)."

-In Figure 3, "1'000" should be "1,000" or "1000."

We thank the reviewer for this helpful comment. We have revised Fig. 3a and Fig. 7b so that the axis labels now use "1,000" instead of "1'000." We have also harmonized the numerical notation throughout the manuscript and Supplementary Information, using a comma to denote thousands and a dot to denote decimals.

-Page numbers can be removed from supplementary references, table or figure number is informative enough.

We thank the reviewer for this helpful suggestion. We have removed supplementary page numbers from the main text and now refer only to the relevant supplementary figure or table (e.g., "Supplementary Fig. 14" and "Supplementary Table 15"). We have also harmonized the notation in both the main manuscript and the Supplementary Information to better conform with Nature Communications formatting guidelines.

-The authors may wish to consider ordering supplementary tables in the order they appear in the main text to make it easier to follow through.

We thank the reviewer for this constructive suggestion. In the revised Supplementary Information, we have reorganised and renumbered the supplementary tables and figures so that, as far as possible, their numbering follows the order in which they are first cited in the main text. Population-descriptive material now appears at the beginning of Section 1 ("Data and descriptive statistics"; Supplementary Tables 1–5 and Supplementary Figs. 1–3), followed by sections on extended time-window exposure, sensitivity analyses, extended analyses, heterogeneity analyses, and the attribution of non-facility births (see updated Supplementary Table of Contents below). Remaining contextual material on travel time, road length, and climate zones has been grouped in the final section ("Extra information and data"), which is only referenced in later parts of the Methods section.

In addition, we rephrased some sections throughout the manuscript and corrected minor writing and typographical errors to improve clarity and readability. We hope that this reorganisation and editing make it easier for readers to navigate between the main text and the Supplementary Information.

Contents

1 Data and descriptive statistics **1**
- 1.1 DHS survey - waves . . . 1
- 1.2 Summary statistics . . . 2
- 1.3 Extreme precipitation events definition . . . 9

2 Extended time-window exposure **10**

3 Sensitivity analysis **11**
- 3.1 Inclusion of live births with missing precipitation records . . . 11
- 3.2 Probit regression . . . 11
- 3.3 Anticipation and spillover effects . . . 12
- 3.4 Placebo . . . 13
- 3.5 Robustness to recall and selection bias . . . 14
- 3.6 Robustness to different extreme precipitation indices . . . 14
- 3.7 Robustness to different levels of fixed effects . . . 16

4 Extended analysis **17**
- 4.1 Sustained (accumulation-window) rainfall exposure model . . . 17
- 4.2 Effect of EPEs across facility types and on skilled birth . . . 21

5 Heterogeneity of EPE impacts **22**
- 5.1 Heterogeneity in effect of EPEs: wealth . . . 22
- 5.2 Heterogeneity in effect of EPEs: travel time . . . 23
- 5.3 Heterogeneity in effect of EPEs: perceived access, motorized vehicle . . . 24
- 5.4 Heterogeneity in effect of EPEs: road length . . . 25
- 5.5 Heterogeneity in effect of EPEs: climate zone . . . 26

6 Non-facility births attributed to extreme precipitation events in 2015 **27**
- 6.1 Cross-country heterogeneity in non-facility births related to EPEs . . . 29

7 Extra information and data **31**
- 7.1 Travel time . . . 31
- 7.2 Road length . . . 35
- 7.3 Climate zone classification of DHS clusters . . . 38
